# The effects of neuron morphology on graph theoretic measures of network connectivity: the analysis of a two-level statistical model

**DOI:** 10.3389/fnana.2015.00076

**Published:** 2015-06-10

**Authors:** Jugoslava Aćimović, Tuomo Mäki-Marttunen, Marja-Leena Linne

**Affiliations:** ^1^Computational Neuroscience Group, Department of Signal Processing, Tampere University of TechnologyTampere, Finland; ^2^Psychosis Research Centre, Institute of Clinical Medicine, University of OsloOslo, Norway

**Keywords:** network connectivity, neuron morphology, theoretical model, neurite density field, graph theory, motifs

## Abstract

We developed a two-level statistical model that addresses the question of how properties of neurite morphology shape the large-scale network connectivity. We adopted a low-dimensional statistical description of neurites. From the neurite model description we derived the expected number of synapses, node degree, and the effective radius, the maximal distance between two neurons expected to form at least one synapse. We related these quantities to the network connectivity described using standard measures from graph theory, such as motif counts, clustering coefficient, minimal path length, and small-world coefficient. These measures are used in a neuroscience context to study phenomena from synaptic connectivity in the small neuronal networks to large scale functional connectivity in the cortex. For these measures we provide analytical solutions that clearly relate different model properties. Neurites that sparsely cover space lead to a small effective radius. If the effective radius is small compared to the overall neuron size the obtained networks share similarities with the uniform random networks as each neuron connects to a small number of distant neurons. Large neurites with densely packed branches lead to a large effective radius. If this effective radius is large compared to the neuron size, the obtained networks have many local connections. In between these extremes, the networks maximize the variability of connection repertoires. The presented approach connects the properties of neuron morphology with large scale network properties without requiring heavy simulations with many model parameters. The two-steps procedure provides an easier interpretation of the role of each modeled parameter. The model is flexible and each of its components can be further expanded. We identified a range of model parameters that maximizes variability in network connectivity, the property that might affect network capacity to exhibit different dynamical regimes.

## 1. Introduction

We analyze how the low-resolution properties of single neuron morphology constrain the connectivity within a large population of neurons. We develop a two-level framework that includes details of single cell morphology while allowing the analysis of large populations of neurons as well as the derivation of compact analytical expressions for most of the considered aspects of morphology and connectivity. The presented framework can further be extended to take into account additional aspects of neuronal morphology and additional properties of connectivity.

In this work, single neurons and neurites are modeled statistically. Each axon and each dendrite is represented by a single neurite field, the probability distribution that describes the density of the neurite branches within a limited area of the neurite. This way each neuron consists of one neurite field for the dendrite, one for the axon, and the parameter that determines the average distance between the dendrite and axon centers. The adopted neurite field model is discussed in the literature. The studies in Snider et al. (2010) and Teeter and Stevens (2011) propose a universal method to describe different neuronal types based on the description of neurite fields of dendrites. A study in Cuntz (2012) demonstrates how realistic neuronal morphologies arise when dendrite segments, distributed according to Snider et al. (2010), get connected using the optimal wiring principle. In van Pelt and van Ooyen (2013) the realism of the obtained synaptic distributions and connectivity probabilities was tested for neurons modeled using density fields.

The use of graph theoretic measures to quantify neuronal connectivity is a methodology adopted from the classical studies of network theory. In various studies and different contexts, it has been demonstrated how such measures can distinguish between functionally different network types. The methodology has been applied to very different networks, from computer networks to social networks, and from gene regulatory networks to neuroanatomy (Boccaletti et al., 2006). Theoretical studies, on the other hand, focus on analysis of generic networks of coupled oscillators demonstrating how statistical properties of network connectivity change the overall dynamics of the complex system. A particularly interesting question in such studies is the search for connectivity that optimizes some aspects of network functionality. Some commonly addressed concepts include small-world networks that minimize the average distance between network nodes while maximizing the cooperation across the node neighborhood. Another concept is the scale-free network that installs system dynamics on the edge between order and disorder, thus maximizing the repertoire of dynamical regimes that a system can exhibit as well as the information diversity in the system (Boccaletti et al., 2006; Mäki-Marttunen et al., 2011). Small-world networks were first introduced in Watts and Strogatz (1998), and then addressed in other studies, also in the neuroscience context (Boccaletti et al., 2006; Herzog et al., 2007; Kriener et al., 2009; Voges et al., 2010; Sporns, 2011; McAssey et al., 2014). They were often examined in the context of the large-scale recordings of whole-brain activity, or the anatomical large-scale connectivity between brain regions (Sporns, 2011). For the smaller-scale networks of individual neurons it is relatively difficult to estimate the small-world property as it requires tracking the synaptic connectivity between neurons in large populations (particularly in order to estimate path lengths). Most of the studies present in the literature examine theoretical concepts through mathematical models, or analyze functional connectivity estimated from recordings. In our previous study, we examined a large repertoire of connectivity measures aiming to find a consistent descriptor of connectivity that has implications on network dynamics (Mäki-Marttunen et al., 2013). Two measures were distinguished, the clustering coefficient for networks with binary distribution of node degrees, and maximal eigenvalue for networks with more variability in the in-degree distribution.

In this study, we primarily focus on the estimation of motif counts (Milo et al., 2002). Motifs represent minimal networks with structured connectivity and are as such suitable for experimental studies. In three previous studies, the non-random distribution of motifs was demonstrated in small networks of pyramidal cells (Song et al., 2005; Perin et al., 2011), and also in networks of interneurons (Rieubland et al., 2014). The implications of these non-random features of connectivity are yet to be explained. Using a theoretical model we derived closed-form expressions for motif counts that do not depend on the network size, but only on the average density of neurons. In addition, the clustering coefficient, that was already found to significantly affect the network activity (Mäki-Marttunen et al., 2013), can be straightforwardly computed from motif counts, as demonstrated in what follows.

A relatively large part of the paper is dedicated to analytical approach to solving the considered two-level model as well as the obtained closed-form solutions. Understanding different levels of organization in neuronal systems and the interaction between those levels is a frequently discussed issue in computational neuroscience literature (Frégnac et al., 2007; Deco et al., 2008). Even the detailed single-level models can become computationally exhaustive and complex, and combining them into multilevel models leads to an explosion in complexity that can obscure the interactions between particular model components. A suggested alternative is the mean-field approximation of each level before linking it to higher-levels of organization (Deco et al., 2008; Sompolinsky, 2014). The presented study complies with this methodology. We first analyze the level of neurons in order to derive simple properties relevant for the network level in the model. In this way, the dimensionality of that level is compressed, which provides the possibility of deriving simpler expressions for the second level characteristics.

Several approximations were adopted when constructing the model of this paper. The neurite structure is described statistically and the fine details of neurite structure are lost. The fine patterns of synaptic distribution are also averaged out. The organization of neurons in the space is chosen to be simple and corresponds to cell cultures more so than to the cortical tissue. Finally, the activity-dependent synaptic reorganization is not considered in this study. Synapses are formed solely based on geometry, and the obtained connectivity corresponds more to potential connectivity as defined in Stepanyants and Chklovskii (2005). In the discussion, we will address some relevant properties of neuronal systems that are not part of the model, and propose a way to incorporate them in the presented framework.

The main result of this study are the analytical expressions for several frequently addressed network measures, including motif counts, clustering coefficient, and path length between network nodes. We particularly addressed motif counts, as they represent the smallest possible networks with structured connectivity. As they capture only the local properties of connectivity, they can be measured experimentally, as demonstrated in Song et al. (2005), Perin et al. (2011), and Rieubland et al. (2014). In addition, the clustering coefficient can be straightforwardly computed from motif counts. From the clustering coefficient and path length, we computed small-world coefficients using two definitions from the literature (Watts and Strogatz, 1998; Telesford et al., 2011). The addressed connectivity measures depend on several model parameters. Some of the parameters contribute as multiplicative constants, while others show non-linear relations to the considered measures. The most interesting parameter is the ratio between the *effective radius* of a neurite and the distance between the axon and dendrite centers of the same neuron. The effective radius is the maximal distance that permits a connection between two neurons. Depending on this ratio, a network can have a connectivity similar to uniform random, or similar to locally coupled network. The most interesting situations are in between these two extremes, where the network increases variability in its connectivity repertoire.

## 2. Methods

To address the principal goal of this study, in other words, to analyze how neuronal morphology can affects connectivity in large networks, we constructed a two-level model. The first level specifies the anatomic properties of each neurite statistically, by defining a probability distribution of neurite branches. The probability distribution is non-zero only within a limited area, the support of neurite distribution. This low-resolution description of neurites was already analyzed in several studies (Snider et al., 2010; Teeter and Stevens, 2011; van Pelt and van Ooyen, 2013). It depends on a small number of parameters, four for the two-dimensional neurites, and is suitable for the analysis of large-scale network connectivity. The second level defines the properties of the neuronal population. In order to emphasize neuron morphology we selected the simplest network model, a two-dimensional virtually infinite-size network with a uniform distribution of neurons. Every pair of sufficiently close axon-dendrite branches forms synapses, the number of synapses is proportional to the axon-dendrite overlap (Peters' rule, Peters and Feldman, 1976; Peters et al., 1991). The obtained synapses correspond to potential connectivity as defined in Stepanyants and Chklovskii (2005). Activity dependent synapse formation and pruning was not considered in this study, although it has been shown to play an important role in remodeling synaptic patterns. Including the activity-dependent mechanisms would require a dynamical model with a more complex synapse formation rule, eventually also described statistically. Activity-induced modifications of neurite distribution might also be considered. In this study, we wanted to analyze a simpler model where the role of morphology was emphasized, as it is the most stable among several properties that shape the connectivity in large networks. The concepts presented here can be combined with models of other relevant mechanisms, including the models of network activity, e.g., the one described in Mäki-Marttunen et al. (2013).

The first part of Methods Section gives a detailed description of the analyzed model. The second part presents the analysis of neurite distribution and shows how its properties determine first-order connectivity statistics under the adopted synapse connectivity rule. In the third part, we present closed-form analytical expressions for the two network measures and an iterative method to obtain another measure frequently addressed in the literature (Sporns, 2011).

### 2.1. Model description

**Figure 1:**
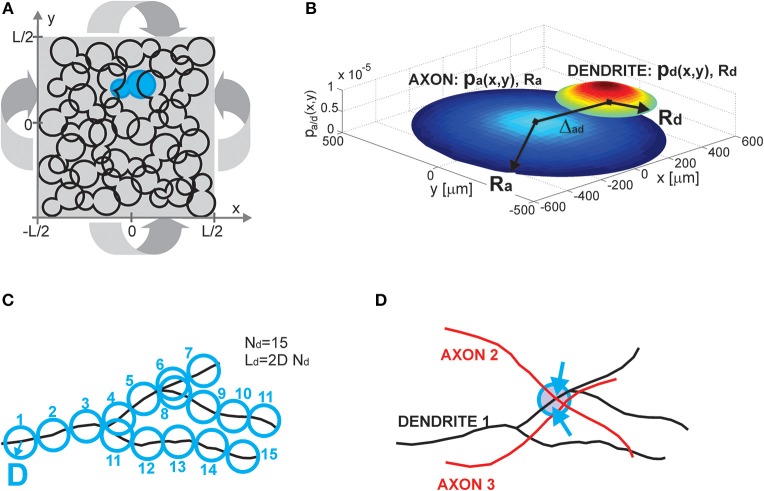
**(A) Population of neurons**. Each neuron is illustrated as an 8-shaped surface (to make it more visible, one such surface is colored blue). The population of neurons is homogenous, all of the neurons have identical properties and they are randomly oriented. The population lays in the planar space of the size *L* × *L*. The dimension *L* is chosen to be much bigger than the size of the neurons. To avoid boundary conditions, the plane is projected on a torus (indicated by four arrows). **(B) Neuron and neurite models**. The axon (a) and dendrite (d) are modeled as density distributions *p_a/d_(x,y)* on a limited circular support with radii *R_a/d_*. The axon and dendrite centers are at a distance Δ*_ad_*. The example in **(B)** shows the neurites modeled as truncated Gaussians with the parameters: (axon) *R*_*a*_ = 500 μ*m*, σ_*a*_ = 0.9 *R*_*a*_, (dendrite) *R*_*d*_ = 200 μ*m*, σ_*d*_ = 0.7 *R*_*d*_, and the distance between neurite centers Δ_*ad*_ = 400 μ*m*. The *x* and *y* axes are in [μ*m*], the z axis shows the value of density distribution for the given coordinates (*x*, *y*). **(C) Neurite segments and density fields**. Each neurite is divided into segments of length 2*D*, and a circle of radius *D* can be circumscribed around the middle of the segment. For a dendrite with *N*_*d*_ segments, the total dendrite length is *L*_*d*_ = 2*DN_d_*. Neurite distribution describes the probability of finding individual segments within the neurite support. It is derived by superimposing many neurites of the same type. **(D) Potential synapse formation rule**. An axon-dendrite pair can form a synapse if an axon segment crosses the near neighborhood of a dendrite segment, the near neighborhood is a circle of radius *D* circumscribed around the dendrite segment (blue circle in the figure). The dendrite segment can form at most one synapse with the considered axon, but it can at the same time form a synapse with every other axon that crosses its near neighborhood (a dendrite segment with two synapses shown in the figure, the two arrows indicate synapse positions).

The model consists of several components, including a neuronal population description, single neuron and single neurite description, and the rule for establishing contacts between neurons (i.e., potential synapses). All these components are illustrated in Figure 1.

#### 2.1.1. Population of neurons (Figure 1A)

Neurons are distributed randomly in the two-dimensional space of the size *L* × *L*, where *L* is chosen to be much bigger than the neuron size, thus making the space around each neuron virtually infinite. The population of neurons is homogeneous, all of the neurons have identical properties and they are randomly oriented in space. The neurons are uniformly distributed in space with the density equal to $\frac{1}{l^{2}}$, i.e., a square of the size *l* × *l* contains on average one neuron, which gives a total of 
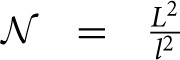
 neurons. To avoid boundary conditions, the edges of the surface are wrapped to form a torus and provide virtually infinite space (which is illustrated in Figure 1A). The model corresponds to the arrangement of neurons in dissociated neuronal cultures. A model of the cortical tissue, on the other hand, requires a non-uniform arrangement of neurons that should follow the distribution of the considered cell types across layers. In addition, the non-random orientation of neurons could be imposed.

#### 2.1.2. Neuron and neurite models (Figure 1B)

All of the neurons in the model are identical and consist of two neurite fields, one for the (basal) dendrite and one for the axon. The dendrite is centered in the soma and the axon center is at a distance Δ*_ad_* from the soma. For the uniform distribution of somata and the random orientation of axons, the distribution of axon centers becomes equal to the one of somata. The neurites are modeled statistically, as a distribution of neurite segments on a finite area, the distribution support. In this study, we considered circular supports with a radius *R*_*a*_ for axons and *R*_*d*_ for dendrites, where *R*_*a*_ ≥ *R*_*d*_. We analyzed cases with uniform and truncated Gaussian distributions of neurites, described by density functions *p*_*a*_(*x, y*) for axons and *p*_*d*_(*x, y*) for dendrites. The expression for the uniform distribution is given by Equation (1) and for the truncated Gaussian by Equation (2), with parameters (*x*_*a/d*_, *y*_*a/d*_)—the coordinates of the axon and dendrite centers, σ_*d*_, σ_*a*_—the variances along both axes.

$$C_{a/d}=1-\exp\left(-\frac{R_{a/d}^{2}}{2\sigma_{a/d}^{2}}\right)$$

are the normalization coefficients that compensate for the cut off part of Gaussians. The presented results can be extended to more general forms of density distributions and elliptic distribution supports.

(1) $$p_{a/d}(x,y)=\{\begin{array}{cc} \frac{1}{R_{a/d}^{2}\pi }, & {\left(x-x_{a/d}\right)}^{2}+{\left(y-y_{a/d}\right)}^{2}\leq R_{a/d}^{2}\\ 0, & \text{else} \end{array}$$

(2) $$p_{a/d}(x,y)=\{\begin{array}{c} \frac{1}{2\pi \sigma_{a/d}^{2}\;C_{a/d}}\exp\left(-\frac{{\left(x-x_{a/d}\right)}^{2}+{\left(y-y_{a/d}\right)}^{2}}{2\sigma_{a/d}^{2}}\right),\\ 0,\\ {\left(x-x_{a/d}\right)}^{2}+{\left(y-y_{a/d}\right)}^{2}\leq R_{a/d}^{2}\\ \text{else} \end{array}$$

#### 2.1.3. Neurite segments and density fields (Figure 1C)

We introduce the maximal number of neurite segments, *N*_*a*_ for axons and *N*_*d*_ for dendrites, for two reasons. First, this concept allows us to compute the expected number of synapses between an axon-dendrite pair, which is an important first step in the derivation of the considered connectivity measures. Second, it connects the individual neurites with the statistical description of neurite fields, which is illustrated in Figure 1C. Each neurite is discretized into segments of length 2*D*. In what follows we will call *D* the unit length of a neurite, so each neurite segment is two units long. If the total length of a neurite is *L*_*a/d*_, then *L*_*a/d*_ = 2*DN_a/d_*. The neurite field describes the probability of finding every neurite segment inside the neurite support, and it can be obtained by superimposing many neurites. We assume that the dendrite center coincides with the soma center as we represent all dendrite branches with the same density field.

#### 2.1.4. Potential synapse formation rule (Figure 1D)

We adopted a simple rule that forms synapses between a pair of neurons independently from other neurons in the population, the number of obtained synapses is proportional to the overlap between the two neurites (Peters' rule, Peters and Feldman, 1976; Peters et al., 1991). Consider a dendrite-axon pair, for each dendrite segment we examine its near neighborhood, a ball of radius *D* centered in the segment center (delineated with a blue circle in Figure 1D). If there is any axon segment present in this ball, the potential synapse between these segments is established. If there is more than one axon segment, only one, randomly selected, of them will form a potential synapse with the dendrite segment. Consequently, every dendrite segment can form at most one potential synapse with the considered axon, but it can simultaneously form potential synapses with other axons that cross its near neighborhood. In the example in Figure 1D, the near neighborhood of a dendrite segment is crossed by two axons and two potential synapses are formed (the blue arrows indicate positions of the potential synapses). This is a rather mild constraint on the number of synapses and in a large population of neurons the number of synapses per neurite can become unrealistically high. Still, it is a reasonable assumption when analyzing potential connectivity, as we are interested in estimating the number of all possible contact places, which is much bigger than the number of actually formed synapses. Alternative rules that take into account all the available segments from all the proximal axons can also be defined.

### 2.2. The methodology used to analyze neurites: connectivity between axon-dendrite pairs

#### 2.2.1. Expected number of synapses per neurite

From the neurite description and the adopted synapse formation rule we derived the expression for the expected number of synapses per neurite (*S*, Equation 3). The details of the derivation of the expression are given in the Supplementary Material 1. The same expression was already proposed in the literature to estimate the number of synapses from neurite density fields (Peters et al., 1991; Liley and Wright, 1994; van Pelt and van Ooyen, 2013). In van Pelt and van Ooyen (2013), an equivalent equation was derived using less strict assumptions about the distribution of axonal field than the one adopted in our study.

(3) $$\bar{S}=N_{a}N_{d}D^{2}\pi \int \int_{\Omega_{a}\cap \Omega_{d}}p_{a}(x,y)\;p_{d}(x,y)\;dx\;dy$$

Replacing the expressions for neurite field distributions into this equation gives the final formula for the expected number of synapses

(4) $$\bar{S}=\frac{N_{a}N_{d}D^{2}}{R^{2}\pi }\cdot \phi (\rho ,\eta ,M)=\frac{4N_{a}N_{d}D^{2}}{\Delta^{2}\pi }\rho^{2}\phi (\rho ,\eta ,M).$$

Here, $R=\frac{R_{a}+R_{d}}{2}$ is the average neurite radius, Δ is the distance between the considered axon-dendrite pair of two proximal neurons, $\rho =\frac{\Delta }{2R}=\frac{\Delta }{R_{a}+R_{d}}$ is the normalized distance between the axon-dendrite pair, $\eta =\frac{R_{a}-R_{d}}{R_{a}+R_{d}}$ is the asymmetry index that accounts for the different size of the axons and dendrites, and *M* is the set of parameters that determine the distribution of neurite segments. *M* is an empty set for a uniform distribution and *M* = {σ, *k*_σ_} for the considered case of truncated Gaussian distribution. Here, $\sigma =\frac{\sigma_{d}}{2R}$ is the normalized dendrite distribution variance, and $k_{\sigma }=\frac{\sigma_{d}}{\sigma_{a}}$ is the ratio between the dendrite and axon variances. In what follows, the function ϕ(ρ, η, *M*) will be called distance-dependent expected number of synapses as it describes the dependency between the expected number of synapses and the axon-dendrite distance. This function can be evaluated analytically for the uniform distribution and numerically for the truncated Gaussians, all relevant derivations are given in Supplementary Material 1 and the function is further discussed in Results Section. The only requirement for this function is to be reversible, at least partially. Similarly, the function ρ^2^ϕ(ρ, η, *M*) will be called size-dependent expected number of synapses as it describes the dependency on the average neurite size.

#### 2.2.2. Computation of node degree and effective radius from neurite field distributions (Figure 2A)

**Figure 2:**
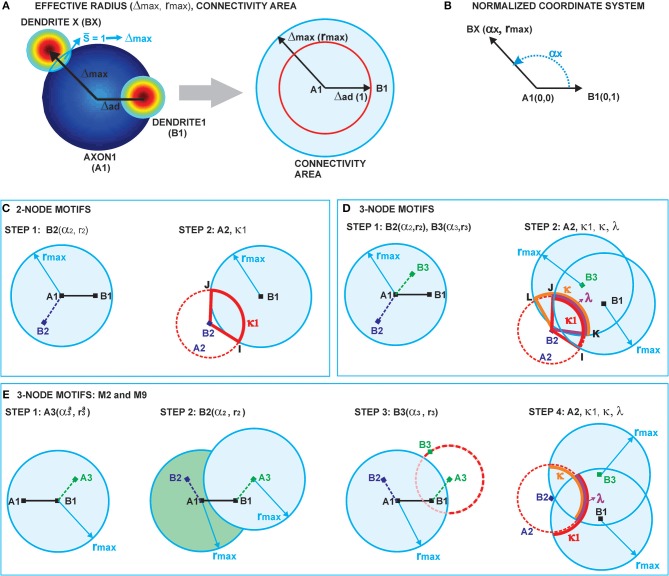
**(A) Definition of the effective radius and the connectivity area**. The effective radius is the distance between an axon center A1 and a dendrite center BX that satisfies the condition *S*(Δ) = 1. Every point within the connectivity area of A1 is at a distance smaller than Δ_*max*_ from A1. **(B) Normalized coordinate system**. The polar coordinate system is fixed to the representative neuron *N*_1_ defined by its axon center A1 and dendrite center B1. The coordinate center is in the axon center A1, and the coordinate axis goes from A1 to the dendrite center B1. The angular coordinate is measured counterclockwise from the coordinate axis. All radial coordinates are normalized, i.e., divided by Δ_*ad*_, so that B1 has coordinates (0, 1) and BX coordinates (α_*X*_, *r*_*max*_), where $r_{max}=\frac{\Delta_{max}}{\Delta_{ad}}$. **(C) 2-Node motif counts**. The panel illustrates two steps in the computation of the expected numbers of 2-node motifs. In the first step, the position of the dendrite center B2 is chosen within the connectivity area of axon A1. In the second step, axon A2 is chosen on the circle of radius 1 around B2 (the red dashed line). The function κ_1_ gives the probability that A2 falls within the connectivity area of B1, κ_1_ is determined by the angle between points B2, I and J. **(D) 3-Node motif counts**. In the first step, the positions of two dendrite centers, B2 and B3, are chosen within the connectivity area of axon A1. The second step defines the position of axon center A2, placed on the circle of radius 1 around B2 (the dashed red line). Intersections of this circle with the connectivity areas of dendrites B1 and B3 define functions κ_1_ (the red line), κ (the orange line), and λ (the purple line), which are determined by the angles ∠*B*2*IJ*, ∠*B*2*KL*, and ∠*B*2*JK*, respectively. The expected number of motifs for all three-node motifs can be computed considering different positions of A2 with respect to the connectivity areas of B1 and B3, and as a combination of functions κ_1_, κ, and λ. **(E) M2 and M9 counts:** Computation of the expected numbers of M2 and M9 requires additional steps. In the first step, the axon center A3 is chosen within the connectivity area of B1. In the second step, the dendrite center B2 is chosen in the connectivity area of A1 but outside the connectivity area of A3 (dark green area). In the third step, the dendrite center B3 is chosen on the circle of radius 1 around A3 (the dashed red line), but outside of the connectivity area of A1 (unshaded part of the dashed red line). The fourth step is identical as the second step in **(D)**.

Two neurons are expected to connect if their axon-dendrite pair has *S* ≥ 1. The expected number of synapses depends on the model parameters (*N*_*a*_, *N*_*d*_, *D*, Δ, *R*) and the normalized parameters ρ, η, and *M*. First we fix all the parameters except Δ (and ρ), and then we find the maximal axon-to-dendrite distance Δ_*max*_ (and ρ_*max*_) which satisfies the condition *S* ≥ 1. This maximal distance is called **the effective radius** of a neurite and its computation is illustrated in Figure 2A. The circle centered in the neurite with the radius equal to the effective radius is called **the connectivity area**. The effective radius integrates the properties of both, the axon and the dendrite, and is consequently equal for both types of neurites. Once it is computed, it simplifies the analysis of network connectivity. Every neuron can be represented as two circles of radius Δ_*max*_ with the distance between the circle centers being Δ*_ad_*. Different network connectivity measures are computed from the intersection of pairs of circles for several neurons.

(5) $$\begin{array}{l} \bar{S}\geq 1\Rightarrow \frac{N_{a}N_{d}D^{2}}{R^{2}\pi }\phi (\rho ,\eta ,M)\geq 1\\ \;\Rightarrow \rho \leq \phi^{-1}\left(\frac{R^{2}\pi }{N_{a}N_{d}D^{2}},\eta ,M\right)\\ \;\Rightarrow \Delta \leq 2R\cdot \phi^{-1}\left(\frac{R^{2}\pi }{N_{a}N_{d}D^{2}},\eta ,M\right)\\ \;\Rightarrow \Delta_{max}=2R\cdot \phi^{-1}\left(\frac{R^{2}\pi }{N_{a}N_{d}D^{2}},\eta ,M\right) \end{array}$$

The function ϕ(·) has to be invertible with respect to the first argument. Here, ϕ^−1^(*x*, η, *M*) means the inverse of ϕ with respect to argument *x* and with η and *M* considered as constants. In case of uniform distribution, the function ϕ is monotonic without discontinuities only for η ≤ ρ < 1. The analysis of this case, shown in Results Section, confirms that the general conclusions still apply.

Finally, the node degree, equal for all the neurons, can be computed as a function of the effective radius. The average number of output connections for a neuron is equal to the average number of dendrite centers within the connectivity area of its axon

(6) $$\begin{array}{l} n_{degree}=\frac{\Delta_{max}^{2}\pi }{l^{2}}=\frac{4R^{2}\pi }{l^{2}}\psi \left(\frac{R^{2}\pi }{2N_{a}N_{d}D^{2}},\eta ,M\right),\\ \text{where }\psi (x,\eta ,M)={\left(\phi {\left(x,\eta ,M\right)}^{-1}\right)}^{2}. \end{array}$$

#### 2.2.3. Constraints on model parameters

So far, no constraints on model parameters were imposed, but obviously a random choice in the 8-dimensional space {*D*, *N*_*a*_, *N*_*d*_, *R*, η, ρ, σ, *k*_σ_} can lead to unrealistic morphologies. In this work, we will not search for biologically realistic parameters using reconstructed neurons or detailed simulations of neurites, e.g., using NETMORPH toolbox (Koene et al., 2009). This will be addressed in our future work. Here, we only give a set of weak conditions necessary for having feasible morphologies.

**Condition 1: Upper bound for the number of neurite segments. Figure 1C** illustrates the discretization of neurites into segments of length 2*D*. A circle of radius *D* is circumscribed around each such segment. As shown in Figure 1C, these circles overlap only immediately after their branching points. As we assume that *D* is small compared to the average segment between two branching points, we can also assume a small number of overlapping circles compared to the total number of circles covering a neurite. If, in addition, we assume that the number of neurite segments should not be too dense, and that the neurites tend to avoid self-intersections, we derive the following upper bound for the number of neurite segments:

$$N_{d}\leq \frac{R_{d}^{2}\pi }{D^{2}\pi },\;\;N_{a}\leq \frac{R_{a}^{2}\pi }{D^{2}\pi }.$$

Right sides of the equations give the approximate number of circles of radius *D* inside the neurite of radius *R*_*d/a*_. For the truncated Gaussian we have an additional relation:

$$N_{a}\leq N_{a}\cdot f\left(\frac{R_{a}}{\sqrt{2}\sigma_{a}}\right)\leq \frac{R_{a}^{2}\pi }{D^{2}\pi },\;\;N_{d}\leq N_{d}\cdot f\left(\frac{R_{d}}{\sqrt{2}\sigma_{d}}\right)\leq \frac{R_{d}^{2}\pi }{D^{2}\pi }.$$

If we replace the parameters (*R*_*a*_, *R*_*d*_, σ_*a*_, σ_*d*_) with the normalized parameters (*R*, η, σ, *k*_σ_) the relation becomes:

(7) $$\begin{array}{l} N_{a}\leq N_{a}\cdot f\left(\frac{(1+\eta )k_{\sigma }}{2\sqrt{2}\sigma }\right)\leq {\left(\frac{(1+\eta )\;R}{D}\right)}^{2},\\ N_{d}\leq N_{d}\cdot f\left(\frac{1-\eta }{2\sqrt{2}\sigma }\right)\leq {\left(\frac{(1-\eta )R}{D}\right)}^{2}. \end{array}$$

The function $f(x)=\frac{x^{2}}{1-\exp(-x^{2})}$ is derived in the Supplementary Material (see Supplementary Material 1, derivation of Equation 4) for the upper bound of *N*_*a*_. The relation for *N*_*d*_ follows from the same analysis when switching the roles of dendrites and axons.

**Condition 2: Weak lower bound for the number of neurite segments**. Each neurite should have at least one connected straight fiber. If the neurite radius is *R*_*d/a*_, the fiber length should be at least 2*R_d/a_*. Clearly, a better approximation for a single fiber would be elliptic support with a longer diagonal equal to *R_a/d_* and a shorter one much smaller than *R_a/d_*. But, if we only consider the circular support of neurites, as it is done in this study, the single fiber of length 2*R_d/a_* is approximated with a circle of the radius *R*_*d/a*_. Therefore, we have

(8) $$N_{a}\geq \frac{2R_{a}}{2D},\;\;N_{d}\geq \frac{2R_{d}}{2D}.$$

**Condition 3: Connected network**. In order to have a connected network the following relation between the model parameters has to hold:

(9) $$n_{degree}\geq 1\Rightarrow \psi \left(\frac{R^{2}\pi }{2N_{a}N_{d}D^{2}},\eta ,M\right)\geq \frac{l^{2}}{2R^{2}\pi }.$$

**Condition 4: The inverse of function** ϕ. The model parameters should be in the range of values where the inverse of ϕ exists:

(10) $$0\leq \frac{R^{2}\pi }{2N_{a}N_{d}D^{2}}\leq \phi_{max}(\eta ,M).$$

**Condition 5: Upper bound for the expected number of synapses**. As each dendrite segment accommodates at most one synapse with a proximal axon, the upper bound of *S* can be estimated as the total number of circles of the radius *D* that can be placed inside the axon-dendrite intersection area:

$$\bar{S}\leq \frac{\left\|\Omega_{a}\cap \Omega_{d}\right\|}{D^{2}\pi }.$$

In cases when the number of neurite segments is much smaller than the neurite radius this upper bound allows more than one synapse per neurite segment, so a more strict constraint should be imposed:

(11) $$\bar{S}\leq \min\{N_{a},N_{d}\}\Rightarrow \phi (\rho ,\eta ,M)\leq \frac{R^{2}\pi }{2D^{2}}\cdot \max\left\{\frac{1}{N_{a}},\frac{1}{N_{d}}\right\}.$$

### 2.3. The methodology used to analyze networks: statistical measures of network connectivity

We analyze network connectivity by computing standard statistical measures, such as motifs, clustering coefficient, harmonic path length, and two versions of small-world coefficient. Most of the section is dedicated to motifs, and the expression for clustering coefficient directly follows from it. The harmonic path length is computed using an iterative procedure. Small-world coefficients are adopted from the literature (Watts and Strogatz, 1998; Telesford et al., 2011) and will only be described in brief. We compute the connectivity measures for one fixed cell, the neuron *N*_1_, which is the representative of all the neurons in the homogeneous population. We consider all the other neurons (*N*_2_, *N*_3_, …, *N*_*k*_) that can form different connectivity patterns with *N*_1_.

#### 2.3.1. Coordinate system and normalization (Figure 2B)

The polar coordinate system is fixed to the neuron *N*_1_, with the axon center A1 and the dendrite center B1. The center of the coordinate system is in A1 and the coordinate axis follows the direction from A1 to B1. The angular coordinate is measured counterclockwise with respect to the coordinate axis and takes values α ϵ [− π, π]. The radial coordinates are normalized, i.e., divided by Δ*_ad_*, so that B1 has the coordinates (0, 1), and a dendrite center BX on the edge of connectivity area has the coordinates $\left(\alpha_{X},r_{max}=\frac{\Delta_{max}}{\Delta_{ad}}\right)$^1^. Figure 2B illustrates the described coordinate system^2^.

#### 2.3.2. Notation

The symbol 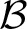
_*R*_*x*__(*X*) is used to denote “a ball” or “a circular neighborhood.” The subscript indicates the normalized radius, and the center of the ball is given between brackets. If the center X has the coordinates (α_*X*_, *r*_*X*_), the ball 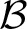
_*R*_*x*__ (X(α_*x*_, *R*_*x*_)) is a set of all points X(α, *r*) such that

$$||A1X||=\sqrt{r^{2}+r_{x}^{2}-2r\;r_{x}\cos(\alpha -\alpha_{x})}\leq R_{x}\cdot$$

This notation is also used to mark the connectivity area of a neurite, for example 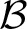
_*r*_*max*__(*A*) is the connectivity area of an axon centered in A. If we replace the inequality in the expression above with an equality the expression corresponds to the edge of the ball, the circle 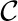
_*R*_*x*__(*X*).

#### 2.3.3. Expected number of two-node motifs (Figure 2C)

Figure 2C illustrates the two-step method for computation of two-node motifs. We consider two connected two-node motifs, i.e., whether two neurons have a unidirectional (*N*_1_ → *N*_2_) or a bidirectional (*N*_1_ ↔ *N*_2_) connection. For the bidirectional motif we will use the notation *M*1 − 2, and for the unidirectional the notation *M*2 − 2. In the first step (the left side of Figure 2C), the position of the dendrite center B2 is chosen inside the connectivity area of axon A1 which, according to the definition of the connectivity area, results in the connection *N*_1_ → *N*_2_. From the model definition, the axon-dendrite distance in a neuron is fixed to Δ*_ad_* (1 in the normalized coordinate system) and the orientation of the neuron is random in the 2D space. Therefore, for the fixed B2 the axon center A2 can take any position on the circle of radius 1 centered in B2, *C*_1_(*B*2), with equal probability. This circle is shown as a red dashed line on the right side of Figure 2C. Given the set of possible positions of A2, we can compute the probability that A2 falls inside the connectivity area of B1, which would give a bidirectional connection between the two neurons. This probability is proportional to the part of the circle *C*_1_(*B*2) that falls inside the connectivity area around B1 (highlighted in Figure 2C), and is also described by the function κ_1_. If A2 is outside the connectivity area of B1, the resulting motif will be the unidirectional connection *N*_1_ → *N*_2_.

From this analysis we can estimate the probability that neuron *N*_2_ forms a unidirectional or a bidirectional motif with the neuron *N*_1_. To compute the expected number of two-node motifs for *N*_1_ we should consider all the possible positions of B2 (and consequently A2) within the connectivity area of A1, which is done by integrating over all the coordinates *B*2(α_2_, *r*_2_) inside the ball 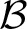
_*r*_*max*__(*A*1). In addition, the expression obtained for the motif *M*2 − 2 is multiplied by two as we should consider two directions of the connection, *N*_1_ → *N*_2_ and *N*_2_ → *N*_1_. The obtained expected numbers of motifs are given by the following expressions:

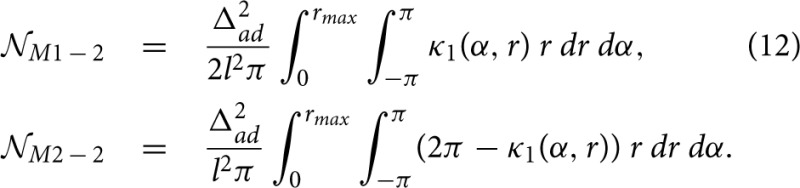



If the effective radius is larger than the axon-dendrite distance in a neuron (Δ_*max*_ > Δ_*ad*_) the dendrite center B1 falls inside the connectivity area of its axon A1. In the considered model, the dendrite centers coincide with the somata and, in general case, they should not be dimensionless. We neglect the finite size of the somata assuming it to be much smaller than the size of the neurite field and the connectivity area. If the somata are not negligible, a correction needs to be applied in order to exclude possibility that some dendrite center overlaps with B1. The correction coefficients for all 2-node and 3-node motifs are given in Supplementary Material 2.

#### 2.3.4. The definition of κ_1_ and κ

The function κ_1_ describes the probability that A2 falls inside the connectivity area of B1, 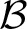
_*r*_*max*__(*B*1), and is proportional to the intersection between this connectivity area and the circle *C*_1_(*B*2). The intersection is determined by the angle ∠*B*2*IJ* shown in Figure 2C, this angle is entirely determined by the coordinates of the dendrite centers *B*1(0, 1) and *B*2(α_2_, *r*_2_). Similarly, we can define a more general function κ if we replace B1 with some other dendrite center *B*3(α_3_, *r*_3_) with arbitrarily chosen coordinates. This way we have κ_1_(α_2_, *r*_2_) = κ(α_2_, *r*_2_, 0, 1)^3^. The function κ is shown by the orange line in Figure 2D, and it is equal to the angle ∠*B*2*KL* shown in the same panel

$$\begin{array}{l} \kappa^{\prime }(\alpha_{2},r_{2},\alpha_{3},r_{3})=2\arccos\left(\frac{1-r_{max}^{2}+d_{23}^{2}}{2d_{23}}\right),\\ d_{23}=\|B2B3\|=\sqrt{r_{2}^{2}+r_{3}^{2}-2r_{2}r_{3}\cos(\alpha_{2}-\alpha_{3})}. \end{array}$$

One special case has to be considered when defining κ′. If the distance between the dendrite centers is smaller or equal to Δ_*max*_ − Δ_*ad*_, i.e., if the circle 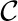
_1_(*B*2) entirely belongs to the connectivity area of the other dendrite, the function κ′(·) becomes complex as its argument becomes larger than 1. However, the intersection angle in this case is 2π. This special case is taken into account in the final definition of κ(·):

(13) $$\begin{array}{l} \kappa (\alpha_{2},r_{2},\alpha_{3},r_{3})=\{\begin{array}{ll} \kappa^{\prime }(\alpha_{2},r_{2},\alpha_{3},r_{3}), & |r_{max}-1|<\|B2B3\|<r_{max}+1\\ 2\pi , & \|B2B3\|\leq r_{max}-1,\;r_{max}\geq 1\\ 0, & \|B2B3\|\geq r_{max}+1\\ 0, & \|B2B3\|\leq 1-r_{max},\;r_{max}<1 \end{array} \end{array}$$

#### 2.3.5. Three-node connectivity patterns (Figure 2D)

Figure 2D describes the two-step procedure needed to evaluate the expected number of the majority of three-node motifs. In the first step, two dendrite centers B2 and B3 are placed inside the connectivity area of the axon A1, which ensures the connections from *N*_1_ to *N*_2_ and *N*_3_. In the second step, the position of the axon center A2 is chosen on the circle 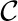
_1_(*B*2) around the dendrite center B2. The intersections of this circle with the connectivity areas around B1 and B3 determine possible connectivity patterns between the three neurons, and the lengths of these intersections are proportional to the probabilities of the connectivity patterns.

The intersection 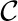
_1_(*B*2) ∩ 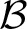
_*r*_*max*__(*B*1) defines the function κ_1_, as in the case of 2-node motifs, which corresponds to the angle ∠*B*2*IJ* in Figure 2D and is colored red. The intersection 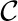
_1_(*B*2) ∩ 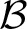
_*r*_*max*__(*B*3) defines the function κ, a generalization of κ_1_, which is shown in orange in Figure 2D and corresponds to the angle ∠*B*2*KL*. If the circle and both connectivity areas intersect, the function λ is non-zero. This is shown in purple in Figure 2D and corresponds to the angle ∠*B*2*KJ*.

If A2 falls inside the connectivity area around B1, but outside of the connectivity area around B3, the neuron *N*_2_ will have a bidirectional connection with *N*_1_ but no connection *toward* *N*_3_ (although, it is possible that it receives a connection from *N*_3_). The probability for this is proportional to the function (κ_1_ − λ). If A2 falls inside the connectivity area of B3, but outside the one of B1, the neuron *N*_2_ receives a unidirectional connection from *N*_1_, and also forms the connection with *N*_3_ (which might be unidirectional or bidirectional, depending on the position of axon A3). Finally, if A2 falls within the intersection between two connectivity areas, neuron *N*_1_ has a bidirectional connection with *N*_2_ and at least a unidirectional connection to *N*_3_.

The same analysis is repeated for the intersections between the circle 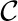
_1_(*B*3), which defines the possible positions of the axon center A3, and the connectivity areas around B1 and B2. This gives the probabilities for the remaining connections. Finally, the following probabilities correspond to the connectivity patterns between the three neurons:

(14) $$N_{2}\to N_{1},N_{3}:\;\frac{1}{2\pi }\lambda (\alpha_{2},r_{2},\alpha_{3},r_{3}),$$

N2→N1, N2→N3:                12π(κ1(α2,r2)−λ(α2,r2,α3,r3)),N2→N3, N2→N1:                12π(κ(α2,r2,α3,r3)                                                            −λ(α2,r2,α3,r3)),N2→N1,N3:                              12π(2π−κ(α2,r2,α3,r3)                                                            −κ1(α2,r2)+λ(α2,r2,α3,r3)),N3→N1,N2:                             12πλ(α3,r3,α2,r2),N3→N1, N3→N2:               12π(κ1(α3,r3)−λ(α3,r3,α2,r2)),N3→N2, N3→N1:               12π(κ(α3,r3,α2,r2)                                                           −λ(α3,r3,α2,r2)),N3→N1,N2:                             12π(2π−κ(α3,r3,α2,r2)                                                           −κ1(α3,r3)+λ(α3,r3,α2,r2)).

The expressions on the right are divided by 2π, as the full circle corresponds to the probability 1.

#### 2.3.6. Definition of λ

The first step is to find the angular coordinates of the intersection points between the circle *C*_1_(*B*2) and the edges of the two connectivity areas, 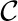
_*r*_*max*__(*B*1) and 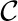
_*r*_*max*__(*B*3). These points are indicated as I, J, K, and L in Figure 2D. The same is done for the intersections between *C*_1_(*B*3) and the edges of connectivity areas around B1 and B2. The following list summarizes these angles:

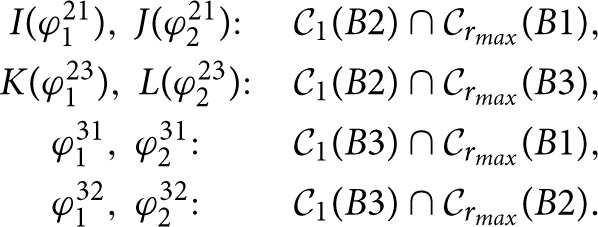



The angles ϕ^21^_1,2_ and ϕ^31^_1,2_ always exist as the corresponding intersections exist for every B2 and B3 inside the connectivity area of A1. The intersections ϕ^23^_1,2_, ϕ^32^_1,2_ exist when *r*_*max*_ ≥ 1, but for *r*_*max*_ < 1 an additional condition for the coordinates of B2 and B3 has to be imposed.

The function λ depends on the length of the arc between these angles, which is independent of the choice of the reference coordinate system. The simplest equations are obtained if we translate the coordinate system from A1 to B2, then rotate it to have the coordinate axis in the direction from B2 to B1. The new coordinate center is B2, while B1 maintains the zero angular coordinate. The first translation requires the following coordinate transform

$$\tilde{r}\cos(\tilde{\alpha })=r\cos(\alpha )-r_{2}\cos(\alpha_{2}),\;\;\tilde{r}\sin(\tilde{\alpha })=r\sin(\alpha )-r_{2}\sin(\alpha_{2}).$$

The second rotation is done by subtracting the angular coordinate of B1 in the translated system, equal to τ(0, 1, α_2_, *r*_2_), from all other angles. The relations between the original coordinates and the coordinates in the translated-then-rotated system are:

$$\begin{array}{l} \;\tilde{r}=\sqrt{r^{2}+r_{2}^{2}-2r\;r_{2}\;\cos(\alpha -\alpha_{2})},\\ \;\tilde{\alpha }=\tau (\alpha ,r,\alpha_{2},r_{2})-\tau (0,1,\alpha_{2},r_{2}),\\ \tau (\alpha ,r,\alpha_{2},r_{2})=\arctan\left(\frac{r\sin(\alpha )-r_{2}\sin(\alpha_{2})}{r\cos(\alpha )-r_{2}\cos(\alpha_{2})}\right). \end{array}$$

Function τ updates the angular coordinates after the translation of the coordinate system to (α_2_, *r*_2_). In the new coordinate system the intersecting angles between *C*_1_(*B*2) and *B*_*r*_*max*__(*B*3) are given as

$$\begin{array}{l} {\tilde{\varphi }}_{1,2}^{23}={\tilde{\alpha }}_{3}\mp \arccos\left(\frac{1-r_{max}^{2}+{\tilde{r}}_{3}^{2}}{2{\tilde{r}}_{3}}\right)\\ \;=\tau (\alpha_{3},r_{3},\alpha_{2},r_{2})\mp \frac{1}{2}\kappa (\alpha_{3},r_{3},\alpha_{2},r_{2}). \end{array}$$

All the relevant intersection angles are:

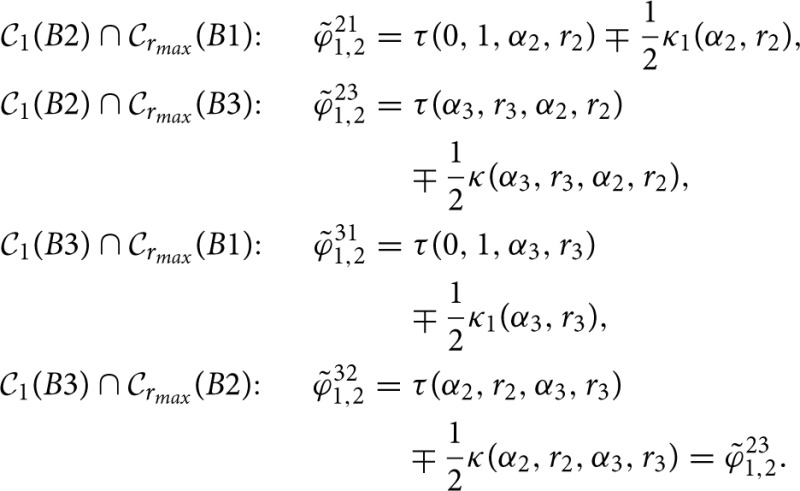



Obtaining the length of the intersection arc from these angles requires considering each possible mutual position of the three angles. This problem was solved using the following procedure. The four angles were sorted from smallest to largest into a vector of angles $\tilde{\varphi }$(α_2_, *r*_2_, α_3_, *r*_3_). The sorted angles parcel the circle *C*_1_(*B*2) into four arcs. For each arc we evaluated the distance between its middle point and the two centers B1 and B3. If both distances are smaller than *r*_*max*_, it indicates that the whole segment belongs to the intersection area *B*_*r*_*max*__(*B*1) ∩ *B*_*r*_*max*__(*B*3). All the segments that passed this test were summed up to obtain the function λ′(α_2_, *r*_2_, α_3_, *r*_3_). This function is non-zero when all three circles intersect. If dendrites B1 and B3 do not overlap, the function is zero. The function can be expressed as

$$\begin{array}{l} \lambda^{\prime }=\sum_{Cond.}\left|\phi_{i}-\phi_{j}\right|\cdot h(r_{max}-d_{i,j}^{1})\cdot h\left(r_{max}-d_{i,j}^{3}\right)\\ \;Cond.:i=1..4,\;j=\text{mod}(i,4)+1,\\ \;d_{i,j}^{k}=\sqrt{\left(1+{\tilde{r}}_{k}^{2}-2{\tilde{r}}_{k}\cos(\phi_{i}+\phi_{j}/2)\right)},\;k=1,3. \end{array}$$

The function *h*(·) is the Heaviside function, equal to one if the argument is positive and equal to zero otherwise. The variables *d*^1^_*i, j*_ and *d*^3^_*i, j*_ are distances from the middle points of the four arcs to the dendrite centers B1 and B3, respectively. The variables **ϕ_*i*_** are the sorted angles from the vector $\tilde{\varphi }$.

If 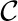
_1_(*B*2) does not intersect with dendrite B1 or B3, the function λ′ is not defined, and the extension of the definition given by Equation (15) is needed. The first case in the list corresponds to the situation when all three circles intersect and the length of the intersection angle is between 0 and 2π. When ∥*B*2*B*3∥ ≤ *r*_*max*_ − 1 the circle 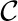
_1_(*B*2) is inside 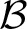
_*r*_*max*__(*B*3) and λ = 2π. On the contrary, when ∥B2B3∥ ≤ 1 − *r*_*max*_, the area 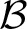
_*r*_*max*__(*B*3) is inside 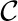
_1_(*B*2) and the function is zero. It is also zero when ∥*B*2*B*3 ∥ ≥ 1 + *r*_*max*_, i.e., when the circle and the area are missing each other.

(15) $$\lambda (\alpha_{2},r_{2},\alpha_{3},r_{3})=\{\begin{array}{ll} \lambda^{\prime }(\alpha_{2},r_{2},\alpha_{3},r_{3}), & \begin{array}{l} \|B1B2\|>r_{max}-1\\ \text{\&}\;|r_{max}-1|<\|B2B3\|<1+r_{max} \end{array}\\ \kappa_{1}(\alpha_{2},r_{2}), & \|B1B2\|>r_{max}-1\;\text{\&}\;\|B2B3\|\leq r_{max}\text{​}-\text{​}1\\ \kappa (\alpha_{2},r_{2},\alpha_{3},r_{3}), & \begin{array}{l} \|B1B2\|\leq r_{max}-1\\ \&\;|r_{max}-1|<\|B2B3\|<r_{max}+1 \end{array}\\ 2\pi , & \|B1B2\|\leq r_{max}\text{​}-1\;\&\;\|B2B3\|\leq r_{max}\text{​}-1\\ 0, & \|B2B3\|\leq 1-r_{max}\\ 0, & \|B2B3\|\geq r_{max}+1 \end{array}$$

#### 2.3.7. Minimal set of connectivity patterns needed to describe three-node motifs, the definition of central node in a connectivity pattern

**Figure 3:**
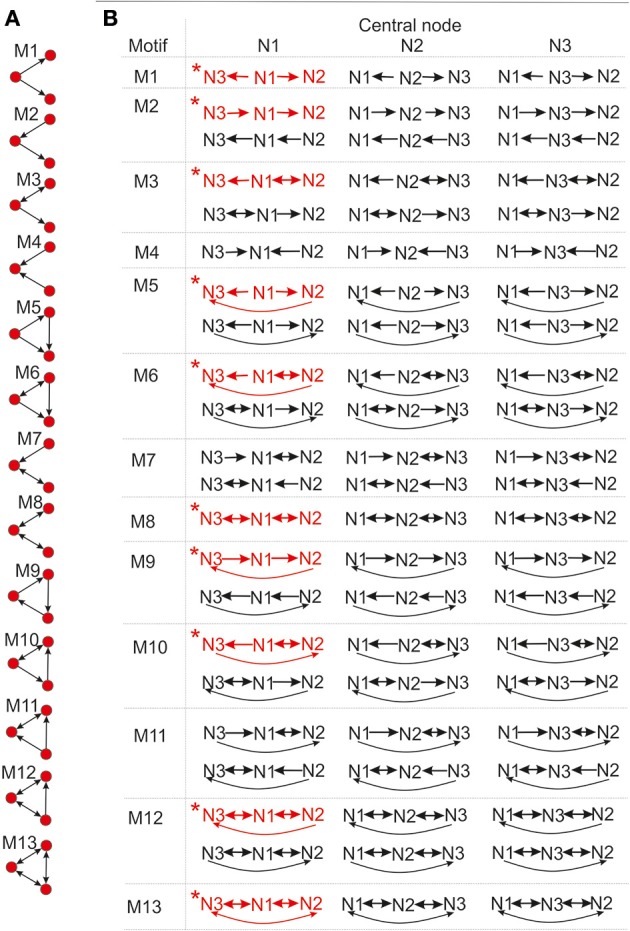
**The schematic representation of all possible 3-node motifs. (A)** The standard representation of motifs (Milo et al., 2002). **(B)** All the possible connectivity patterns between the three (fixed) nodes. Columns 1,2,3 in the table correspond to the nodes *N*_1_,*N*_2_,*N*_3_ being the central node in the connectivity pattern. The complete list of 3-node connectivity patterns can be reduced to the 10 representative patterns (highlighted in red), and only these 10 patterns are considered in computations of the expected motif counts.

To compute the expected numbers of three-node motifs one has to analyze all the possible connectivity patterns between the three neurons *N*_1_, *N*_2_, and *N*_3_, each represented by two circular connectivity areas, one for the dendrite and one the for axon. Figure 3A shows the standard schematic representation of the 3-node motifs (Milo et al., 2002), and Figure 3B shows all the possible connectivity patterns between *N*_1_, *N*_2_, and *N*_3_ that correspond to each of the motifs^4^. We will demonstrate how this full list of patterns can be reduced to 10 representative ones, sufficient to compute the expected counts for all the motifs. These 10 patterns are shown in red in the table and are also marked with the star symbol. The choice of the patterns is somewhat arbitrary and an alternative set can also be adopted, which should not affect the obtained expected numbers of motifs. Reduction to the minimal set of patterns also ensures that each pattern is counted only once.

First, we need to introduce the notion of **central node** n the motif, suppose it is *N*_1_. If *N*_1_ is central to the motifs M1, M3, M5, M6, M8, M10, M12, and M13, both dendrite centers B2 and B3 belong to the connectivity area of axon A1, i.e., *N*_3_ ← *N*_1_ → *N*_2_ has to be included in the connectivity pattern. If *N*_1_ is central to the motifs M4, M7, and M11, the situation is inverse, both axon centers A2 and A3 have to belong to the connectivity area of dendrite B1, i.e., *N*_3_ → *N*_1_ ← *N*_2_ has to be included in the pattern. If *N*_1_ is central to the motifs M2 and M9, the neuron *N*_1_ is on the path from *N*_3_ to *N*_2_, i.e., *N*3 → *N*1 → *N*2 has to be part of the pattern.

The definition of the central node for the three groups of motifs is chosen to emphasize the similarities between the connectivity patterns and to enable selection of the minimal set of patterns. For example, the central node for M11 can be defined the same way as for M1, but the adopted definition emphasizes the similarity between M11 and M6. Following the definition of the central node, all of the patterns are divided into three sets, shown as three columns in Figure 3B. Column *i* contains connectivity patterns where neuron *N*_*i*_ represents the central node. Since all of the neurons in the network have the same properties, the motif counts do not depend on the choice of the central node. Therefore, for counting all the motifs that include the neuron *N*_1_, it is sufficient to count the motifs where *N*_1_ is central and multiply the obtained counts with a coefficient.

Motifs M1, M4, M8, and M13 have one possible pattern with *N*_1_ as the central node, M2, M3, M5, M6, M7, M9, and M10 have two, M11 and M12 have four, but only two should be considered as the other two are repeated in columns two and three. If we further analyze the pairs of patterns that appear in column one, it is evident that one of them can be obtained from the other by switching the positions of *N*_2_ and *N*_3_. Therefore, it is sufficient to consider only one of them, irrelevant which one is chosen (here, we selected the first one). The reason is the following: in order to create patterns from the first group the dendrite centers B2 and B3 have to be inside the connectivity area of axon A1. To compute all the motif counts, we have to consider every possible position of B2 and B3 within 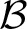
_*r*_*max*__(*A*1). Consequently, both choices of coordinates *B*2 = (α_*a*_, *r*_*a*_), *B*3 = (α_*b*_, *r*_*b*_) and *B*2 = (α_*b*_, *r*_*b*_), *B*2 = (α_*a*_, *r*_*a*_) are considered, as well as both connectivity patterns that correspond to a certain motif. It can also happen that B2 = B3 or B2 = B1 or B3 = B1, but the number of such examples is negligible, as shown in Supplementary Material 2. To count all the occurrences of M2 and M9, we put one dendrite center (B2 or B3) in the connectivity area of axon A1, and one axon center (A3 or A2) in the connectivity area of dendrite B1. Regardless of the neuron numeration, this is sufficient to take into account every appearance of these two motifs.

Next, consider motifs M1 and M4. One of them is obtained from another by switching the orientation of all the connections. This is equivalent to exchanging dendrites and axons, if motif M1 requires B2 and B3 inside the connectivity area of A1, then motif M4 requires A2 and A3 inside the connectivity area of B1. Connectivity areas of dendrites and axons are equal, which means that counts for M1 and M4 must be equal, 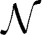
(**M1**) = 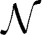
(**M4**). The same holds for motifs M3 and M7, and also for M6 and M11. Consequently, M4, M7, and M11 do not need to be considered separately. This completes the search for the minimal set of patterns that are shown in red in Figure 3B.

Once the counts for the 10 representative patterns are computed, the final motif counts are obtained by multiplying them with the following coefficients: **3 for M2, M3, M5, M7, M10, and M12, 1.5 for M1, M4, M6, M8, and M11, 1 for M9, 0.5 for M13**. The first set of motifs is multiplied by 3 in order to take into account three possible choices of the central node. There is no need to take into account two different patterns for each central node because that is already accounted for by considering all the possible coordinates of B2 and B3, as described in a previous paragraph. Motifs M1, M4, M8 are multiplied by $\frac{3}{2}$, because each central node corresponds to only one pattern. Consequently, the procedure that takes into account all possible positions of B2 and B3 leads to counting every pattern twice. Closer inspection of the patterns for M6, M10, and M11 shows that each pattern in the table in Figure 3B repeats twice, e.g., for M6, pattern 1 for *N*_1_ as the central node is equal to pattern 2 for *N*_2_ as the central node. If we multiply the motif counts for central node *N*_1_ by 3, in order to take into account other choices of central nodes, we actually consider each pattern twice. So the counts should be additionally divided by 2. Next, motifs M9 and M13 are circular and any choice of the central node gives the same pattern. So there is no need to multiply the counts obtained for *N*_1_ by 3. In addition, M13 has only one pattern that corresponds to *N*_1_ as the central node, so the count should be additionally divided by 2.

#### 2.3.8. The expected number of motifs M1, M3, M5, M6, M8, M10, M12, and M13

The expressions for the expected number of 3-node motifs are obtained by combining Equations (14) with the procedure for computing the expected number of 2-node motifs. Equations (14) give probabilities for different types of connections from *N*_2_ to *N*_1_ and *N*_3_, and also from *N*_3_ to *N*_1_ and *N*_2_. The probability for each connectivity pattern from Figure 3 is obtained by multiplying the probability of the appropriate connection from *N*_2_ to *N*_1_ and *N*_3_ with the probability of the connection from *N*_3_ to *N*_1_ and *N*_2_. These probabilities are defined for any pair of coordinates of B2 and B3. In order to form any of the listed motifs, B2 and B3 have to be inside the connectivity area of A1, which defines the range of their coordinates: in the coordinate system fixed to A1, the angular coordinates α_2_ and α_3_ take all the possible values and the radial coordinates *r*_2_ and *r*_3_ have to be smaller than *r*_*max*_. Similarly, as in the case of 2-node motifs we should integrate the expressions for the probabilities of connectivity patterns over all the possible coordinates for both B2 and B3, i.e., over two pairs of coordinates. This results in a quadruple integral, and the coefficient in front of the integral is the square of the coefficient obtained for the 2-node motifs.

The following expression (Equation 16) gives the expected number of the representative connectivity patterns for the motifs from this group. The total motif counts are obtained by multiplying them with the coefficients given in the previous section.

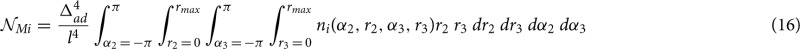



The expression 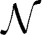
_*Mi*_ corresponds to the motif Mi, and depends on the function *n*_*i*_(α_2_, *r*_2_, α_3_, *r*_3_):

$$\begin{array}{l} n_{1}(\alpha_{2},r_{2},\alpha_{3},r_{3})=\frac{1}{4\pi^{2}}\cdot \left(2\pi -\kappa_{1}(\alpha_{2},r_{2})-\kappa (\alpha_{2},r_{2},\alpha_{3},r_{3})+\lambda (\alpha_{2},r_{2},\alpha_{3},r_{3})\right)\cdot \\ \;\cdot \left(2\pi -\kappa_{1}(\alpha_{3},r_{3})-\kappa (\alpha_{3},r_{3},\alpha_{2},r_{2})+\lambda (\alpha_{3},r_{3},\alpha_{2},r_{2})\right),\\ n_{3}(\alpha_{2},r_{2},\alpha_{3},r_{3})=\frac{1}{4\pi^{2}}\cdot \left(\kappa_{1}(\alpha_{2},r_{2})-\lambda (\alpha_{2},r_{2},\alpha_{3},r_{3})\right)\cdot \\ \;\cdot \left(2\pi -\kappa_{1}(\alpha_{3},r_{3})-\kappa (\alpha_{2},r_{2},\alpha_{3},r_{3})+\lambda (\alpha_{3},r_{3},\alpha_{2},r_{2})\right),\\ n_{5}(\alpha_{2},r_{2}\alpha_{3},r_{3})=\frac{1}{4\pi^{2}}\cdot \left(\kappa (\alpha_{2},r_{2},\alpha_{3},r_{3})-\lambda (\alpha_{2},r_{2},\alpha_{3},r_{3})\right)\cdot \\ \;\left(2\pi -\kappa (\alpha_{3},r_{3},\alpha_{2},r_{2})-\kappa_{1}(\alpha_{3},r_{3})+\lambda (\alpha_{3},r_{3},\alpha_{2},r_{2})\right),\\ n_{6}(\alpha_{2},r_{2}\alpha_{3},r_{3})=\frac{1}{4\pi^{2}}\cdot \lambda (\alpha_{2},r_{2},\alpha_{3},r_{3})\cdot \\ \;\left(2\pi -\kappa_{1}(\alpha_{3},r_{3})-\kappa (\alpha_{3},r_{3},\alpha_{2},r_{2})+\lambda (\alpha_{3},r_{3},\alpha_{2},r_{2})\right),\\ n_{8}(\alpha_{2},r_{2}\alpha_{3},r_{3})=\frac{1}{4\pi^{2}}\cdot \left(\kappa_{1}(\alpha_{2},r_{2})-\lambda (\alpha_{2},r_{2},\alpha_{3},r_{3})\right)\cdot \left(\kappa_{1}(\alpha_{3},r_{3})-\lambda (\alpha_{3},r_{3},\alpha_{2},r_{2})\right),\\ n_{10}(\alpha_{2},r_{2}\alpha_{3},r_{3})=\frac{1}{4\pi^{2}}\cdot \left(\kappa_{1}(\alpha_{2},r_{2})-\lambda (\alpha_{2},r_{2},\alpha_{3},r_{3})\right)\cdot \left(\kappa (\alpha_{2},r_{2},\alpha_{3},r_{3})-\lambda (\alpha_{3},r_{3},\alpha_{2},r_{2})\right),\\ n_{12}(\alpha_{2},r_{2}\alpha_{3},r_{3})=\frac{1}{4\pi^{2}}\cdot \lambda (\alpha_{2},r_{2},\alpha_{3},r_{3})\cdot \left(\kappa_{1}(\alpha_{2},r_{2})-\lambda (\alpha_{3},r_{3},\alpha_{2},r_{2})\right),\\ n_{13}(\alpha_{2},r_{2}\alpha_{3},r_{3})=\frac{1}{4\pi^{2}}\cdot \lambda (\alpha_{2},r_{2},\alpha_{3},r_{3})\cdot \lambda (\alpha_{3},r_{3},\alpha_{2},r_{2}). \end{array}$$

From the definition of κ, κ_1_, and λ, all the functions *n*_*i*_ have discontinuities and therefore cannot be integrated straightforwardly. The problem was solved by dividing the entire domain of integration into sub-domains where the functions are continuous. Then, the integration was performed for each sub-domain and the total motif count is obtained by summing up all of the obtained values. The details are presented in Supplementary Material 2.

#### 2.3.9. The expected number of motifs M4, M7, M11

From the previous discussion, these values are equal to the expected number of motifs M1, M3, and M6, respectively.

#### 2.3.10. The expected number of motifs M2 and M9 (Figure 2E)

The computations for motifs M2 and M9 require a four-step procedure illustrated in Figure 2E. First, the axon center A3, given by coordinates (α^*a*^_3_, *r*^*a*^_3_), is chosen inside the connectivity area of dendrite B1. Next, the dendrite center B2 with coordinates (α_2_, *r*_2_) is chosen inside the connectivity area of A1, but outside of the connectivity area of A3 (the dark green area in Figure 2E). This results in the connectivity pattern *N*_3_ → *N*_1_ → *N*_2_, a necessary condition for both motifs M2 and M9. In the third step, the dendrite center B3(α_3_, *r*_3_) is chosen on the circle 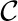
_1_(*A*3), but outside the connectivity area of A1, i.e., in the domain 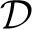
(B3) = 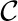
_1_(*A*3) \ 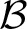
_*r*_*max*__(*A*1). This way, the bidirectional connection between *N*_1_ and *N*_3_ is avoided. If 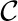
_1_(*A*3) entirely belongs to the connectivity area of A1, motifs M2 and M9 are impossible. Therefore, an additional condition for the coordinates of A3 is: *r*^*a*^_3_ > *r*_*max*_ − 1. In the final step, axon A2 is chosen on the circle 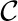
_1_(*B*2). Same as before, the intersection between this circle and the connectivity areas of B1 and B3 defines the probabilities to form motifs M2 and M9. These probabilities are expressed using functions κ_1_, κ, and λ. Motif M2 emerges if A2 falls outside of both connectivity areas, while M9 emerges if A2 falls inside the connectivity area of B3, but outside the one of B1.

The expected numbers of motifs M2 and M9 are computed similarly as before. The probabilities of the representative connectivity patterns are integrated for all possible positions of A3 and B2. In addition, we have to take into account all the positions of B3, which adds the fifth integral to the equations. The easiest way to evaluate this innermost integral is by translating the coordinate system from A1 to A3, to simplify expressions for the coordinates of B3 in 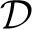
(*B*3) = 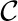
_1_(*A*3) \ 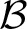
_*r*_*max*__(*A*1). The outer quadruple integral is evaluated in the coordinate system of A1. The obtained expressions for the expected number of motif counts are:

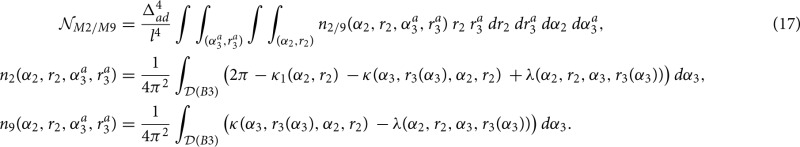



#### 2.3.11. Clustering coefficient (CC)

Clustering coefficient quantifies the density of connections in the local neighborhood of each network node. The percent of connected neighbors is estimated for each network node, and the average over all nodes represents the clustering coefficient (Watts and Strogatz, 1998; Boccaletti et al., 2006). A global measure related to the clustering coefficient is transitivity (Watts and Strogatz, 1998; Boccaletti et al., 2006) which estimates the number of triangles among all the connected triplets in a network. Here, we consider a simple case of identical neurons (network nodes) uniformly distributed in a planar space without boundaries. The clustering coefficient of the resulting network is identical to the local clustering coefficient of each node. Similarly, the global transitivity measure reduces to the measure evaluated for a single node. We employ one possible extension of the original clustering coefficient (for undirected networks) to the case of directed networks (Boccaletti et al., 2006; Sporns, 2011; Telesford et al., 2011; Mäki-Marttunen et al., 2013):

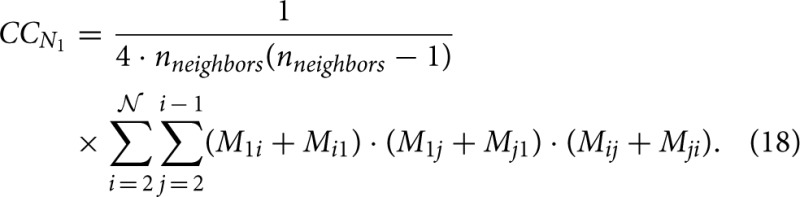



The expression holds for a network of 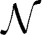 nodes where each node has *n*_*neighbors*_ neighbors. The values *M*_*ij*_ describe the presence or absence of a connection between nodes *i* and *j*, *M*_*ij*_ = 1 if a connection from *i* to *j* exists and *M*_*ij*_ = 0 otherwise. This equation can be re-written as a linear combination of motif counts. We can group all pairs of neighbors of node *N*_1_ according to the motif they form. The number of pairs in each group is equal to the corresponding motif count. Each motif count should be multiplied with the coefficient determined by the product from the summation above. Clearly, if a motif has two unconnected nodes (like M1 or M2) the coefficient is zero. For M5 and M9 the coefficient is 1, for M6, M10, M11 it is 2, for M12 it is 4, and for M13 it is 8. From the previous derivations, the expected motif counts are given by the values 3
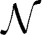
_*M*5_ for M5, 1.5
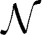
_*M*6_ for M6, 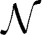
_*M*9_ for M9, 3
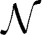
_*M*10_ for M10, 1.5
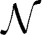
_*M*11_ for M11, 3
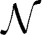
_*M*12_ for M12, 0.5
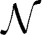
_*M*13_ for M13. The number of neighbors can be expressed using the expected 2-node motif counts, *n*_*neighbors*_ = 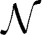
_*M*1−2_ + 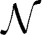
_*M*2−2_, as the sum of unidirected and bidirected connections that start or end in *N*_1_. The equation for the expected clustering coefficient becomes

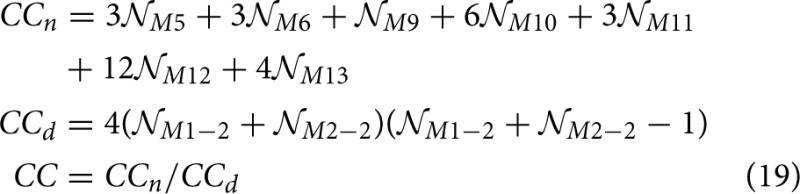



#### 2.3.12. Path length

The path length *PL*_*ij*_ from neuron *N*_*i*_ to neuron *N*_*j*_ is equal to the minimal number of edges on a **traversable** path between them. If the neurons are unconnected then *PL*_*i, j*_ = ∞. If *PL*_*i, j*_ = *k* > 1, no direct connection between the two neurons exists. Instead, the path from one of them to the other goes through *k*−1 other neurons. We compute the harmonic path length (Watts and Strogatz, 1998; Boccaletti et al., 2006; Mäki-Marttunen et al., 2011), the harmonic mean over the shortest path lengths for all the pairs of neurons in the network. In the population of identical, randomly oriented and uniformly distributed neurons, this coefficient becomes equal to the harmonic path length computed for one fixed neuron, for example neuron *N*_1_, as follows

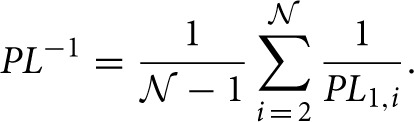



Instead of computing the harmonic mean we use an equivalent expression for the expected harmonic path length

(20) $$PL^{-1}=\sum_{k=1}^{+\infty }\frac{1}{k}\cdot P(PL=k).$$

There, *P*(*PL* = *k*) is the probability that the shortest path from *N*_1_ to some other node goes through *k* direct edges, i.e., through *k*−1 other nodes. For sufficiently large networks, the mean converges toward the expected value, which should hold for the considered model. In the derivations that follow, all the coordinates are expressed in the coordinate system fixed to neuron *N*_1_, as it was described before. In this coordinate system, the path length from *N*_1_ to a specific neuron *N*_*X*_ depends only on the radial but not on the angular coordinate of *N*_*X*_, so we can fix the angular coordinate to α_*X*_ = 0 and consider only the neurons along the coordinate axis.

The probability of the shortest path length *P*(*PL* = *k*) is computed using the following expression

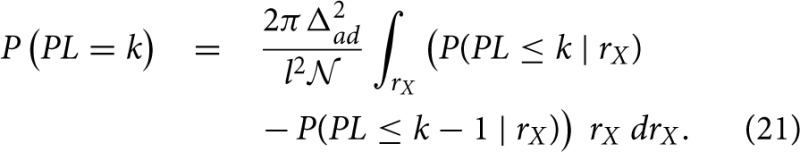



where the integration is done over the radial coordinate *r*_*X*_. The integrated function is the joint distribution of path length and radial coordinate. The joint distribution is expressed as the product of the shortest path length distribution conditioned on the radial coordinate and the probability that a dendrite center has such radial coordinate. The probability of having a radial coordinate *r*_*X*_ is simply expressed as the number of dendrite centers within a ring with the radius *r*_*X*_ divided by the total number of neurons 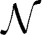. The path length distribution conditioned on the radial coordinate is expressed using another conditional probability, *P*(*PL* ≤ *k* | *r*_*X*_). For the fixed radial coordinate, this probability shows how likely is that the shortest path length of the considered neuron does not exceed *k*.

The last conditional probability is obtained from the following analysis. Consider a neuron *N*_*X*_ and fix its dendrite center to *r*_*X*_. If it has the shortest path length at most *k*, then there must be one other neuron that connects to *N*_*X*_, i.e., that has its axon center within the connectivity area of the dendrite *B*_*X*_, and that has the shortest path length no bigger than *k*−1. Clearly, this is opposite to the statement that every neuron either does not connect to *N*_*X*_ or has the shortest path length bigger than *k*−1. If we express this formally, as probabilities of the described events, and consider all neurons independent on each other we can write the conditional probability as

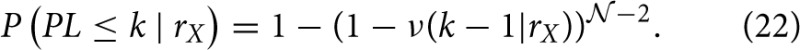



The last equation depends on the assisting function ν(*k* − 1|*r*_*X*_). If we consider one particular neuron with the fixed coordinates, the probability that it connects to *N*_*X*_ and has the path length at most equal to *k*−1 is described by ν(*k* − 1|*r*_*X*_). Finally, this function can be expressed as a function of the conditional probability

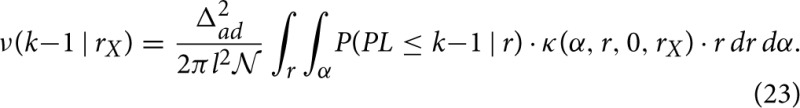



The expressions for the conditional probability and the ν-function form a pair of iterative equations that should be computed for all feasible values of *k*. The definition of connectivity area gives the initial condition for these equations

$$P\left(PL\leq 1\;|\;r_{X}\right)=\{\begin{array}{c} 1\;r_{X}\leq r_{max}\\ 0\;r_{X}>r_{max} \end{array}$$

The obtained expressions are different from the methodology used for motif counts or clustering coefficient. The harmonic path length represents a global measure of network structure and consequently depends on the total number of neurons in the population. The equations derived here are carefully analyzed in Supplementary Material 3. Every step in the presented procedure is illustrated. An equivalent model is simulated and the results from the theoretical model (from this section) and the simulated model are shown alongside.

#### 2.3.13. The small-world coefficient

The clustering coefficient and the shortest path length are sufficient for the computation of the small-world coefficient. We consider two different definitions. The classical definition of the small-world coefficient (Watts and Strogatz, 1998) is the following:

$$SW_{ws}=\frac{CC/CC_{random}}{PL/PL_{random}}.$$

Here, the clustering coefficient CC and the shortest path length PL of the considered network are compared to those of a uniform random network. In a small-world network, the clustering coefficient should be relatively high, similarly to the situation in lattice networks, while the path length should be short, similarly to the case of uniform random networks. Therefore, the SW coefficient should be close to one for the uniform random networks and much bigger than one for the small-world networks.

Additionally, we consider another definition from the literature introduced in Telesford et al. (2011) that compares a network with both, uniform random and locally coupled networks

(24) $$SW_{q}=\frac{PL_{random}}{PL}-\frac{CC}{CC_{local}}.$$

For a network similar to the uniform random one, the first factor $\frac{PL_{random}}{PL}$ should be close to one while the second factor $\frac{CC}{CC_{local}}$ becomes very small as uniform random networks have a much smaller clustering coefficient than locally coupled networks. Therefore, *SW*_*q*_ is positive and close to one. For a network similar to a locally coupled network, the first factor is small, as the PL of such networks is much larger than in random networks, while the second factor is close to one. The coefficient *SW*_*q*_ is negative and close to minus one. In case of small-world networks, both the first and the second factor are close to one and *SW*_*q*_ is close to zero.

#### 2.3.14. Locally coupled networks

The locally coupled networks are generated to correspond to the extreme situation in our model, the overlapping axon and dendrite centers (Δ_*ad*_ = 0). The number of 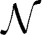 nodes is uniformly distributed in the two-dimensional space (of size *L* × *L*) with the density equal as before (i.e., equal $\frac{1}{l^{2}}$). The two-dimensional space is projected on a torus to avoid boundary conditions. The number of nodes is sufficiently bigger than the maximal considered node degree. A node is connected to every other node inside its connectivity area, which gives the node degree according to Equation (6). A network generated this way has only bi-directional connections and can express only motifs M2-1, M8, and M13, we call it “strictly locally coupled network.” To increase variability in motif counts and still maintain the properties of a locally coupled network, we removed 10% randomly selected connections and established them with the nearest neurons outside the connectivity area, we refer to it as “locally coupled network with 10% of non-local connections.”

#### 2.3.15. Uniform random networks

These networks are generated in a standard way. Each connection is set with the probability 
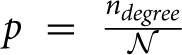
 independently on other connections. Clearly, the finite size of these networks raises some issues. In the analyzed model networks, the total number of nodes is explicitly considered only when computing path length through the network. The network is considered virtually infinite. There is no boundary conditions and each node has an equal number of available neighbors. In the locally coupled networks, as described above, a comparable model is provided by choosing a large enough network and projecting it on a torus. In uniform random networks, the problem is somewhat more difficult because the network size determines probability of connection, the parameter that affects all considered network measures. In the results presented here, we fix the network size and the probability of connection solely varies with the node degree.

## 3. Results

The results of the model analysis are divided into two parts, similarly to the model description. In the first part, the properties of neurite morphology are related to the connectivity between pairs of neurons. Quantitative measures such as the expected number of synapses, the effective radius of the connectivity area, and the node degree are derived as functions of the neurite model parameters. In the second part, the concept derived in the first step, the effective radius, is related to the typical measures used to quantify connectivity in networks, motif counts, clustering coefficient, path length, and small-world coefficient. This way we divided the initial question, how the properties of neurite morphology affect connectivity in large networks, into two easier goals that better explain the role of different aspects of the model.

### 3.1. The expected number of synapses

In this section, we show how the expected number of synapses *S* depends on the neuron model parameters. We give the general expression for this dependency in Methods Section by Equation (4). The derivation of *S* is given in Supplementary Material 1. We consider the neurites with circular support, i.e., with neurite segments distributed inside the circle of radius *R*_*a*_ for axons and *R*_*d*_ for dendrites, and with one of the two forms of distributions, uniform or truncated Gaussian. The truncated Gaussians have equal variances along the two dimensions and the zero cross-covariance, the cases that simplify computations. Neurite distributions are described by the parameter set *M*, which is an empty set for the uniform distribution and contains normalized parameters *M* = {σ, *k*_σ_} for the truncated Gaussians. The presented methodology can be applied in more general situations, for neurites with elliptic support and a general form of truncated Gaussian distribution.

According to Equation (4), *S* depends linearly on the number of axon and dendrite segments, *N*_*a*_ and *N*_*d*_, and also on the square of the unit length *D*. It has a non-trivial dependency on the axon-dendrite distance Δ, on the average neurite size *R*, and on their ratio, the normalized axon-dendrite distance ρ. In addition, it depends on the asymmetry index η, the parameter that quantifies asymmetry between the size of axons and dendrites. This parameter takes values from the interval η ϵ [0, 1], for η = 0 the dendrite and axon radii are the same (*R*_*a*_ = *R*_*d*_), and for η → 1 the axons are much bigger than dendrites (*R*_*a*_ >> *R*_*d*_). In the considered model, the axons are always bigger than the dendrites. Finally, *S* depends on the neurite density distributions and the set of normalized parameters *M*.

#### 3.1.1. The expected number of synapses as a function of axon-dendrite distance (Figures 4A–D)

**Figure 4:**
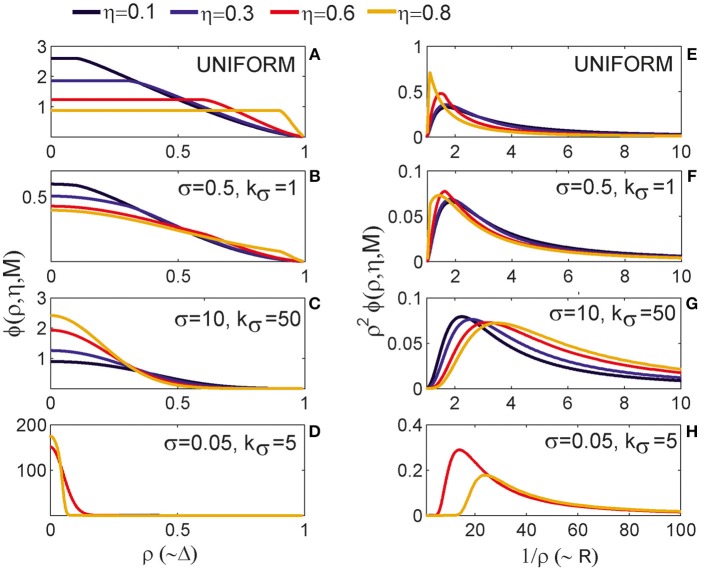
**Expected number of synapses. Left column:** Distance-dependent expected number of synapses [ϕ(ρ, η, *M*)] as a function of the axon-dendrite distance (Δ), and consequently the normalized axon-dendrite distance (ρ). When all the parameters but Δ are fixed this function is equal to the expected number of synapses up to a multiplicative constant. **Right column:** Size-dependent expected number of synapses [ρ^2^ϕ(ρ, η, *M*)] as function of the average neurite radius *R* and the inverse of the normalized axon-dendrite distance (1/ρ) which is proportional to the average neurite radius. When all the parameters but the average neurite radius are fixed this function describes the expected number of synapses up to a multiplicative constant. The functions are shown for four values of the asymmetry index (η), dark blue, η = 0.1; blue, η = 0.3; red, η = 0.6; yellow, η = 0.8. **(A,E)** are obtained for the uniformly distributed axon and dendrite segments. **(B–D, F–H)** show three typical examples obtained for the truncated Gaussian distribution of neurite segments, and the normalized parameters of the distribution are indicated on each panel.

We first show how *S* depends on the axon-dendrite distance and on the normalized axon-dendrite distance by fixing all the other parameters. This way the expected number of synapses becomes proportional to the function ϕ(ρ, η, *M*), consequently called the distance-dependent expected number of synapses. This is illustrated in the left column in Figures 4A–D. Different panels correspond to different distributions of neurite segments, which are indicated on each panel along with the distribution parameters. The x-axis in Figures 4A–D shows the axon-dendrite distance (Δ ∈ [0, *R*_*a*_ + *R*_*d*_]) and the normalized axon dendrite distance (ρ ∈ [0, 1]). Four different cases in each panel correspond to different values of the asymmetry index (values for the asymmetry index and the color code are indicated in Figure 4).

Figure 4A illustrates the expected number of synapses obtained when both the axon and dendrite have uniform distribution of neurite segments. In this case, the function is determined solely by the overlap between neurites, i.e., by the parameters that determine the overlap, the (normalized) axon-dendrite distance and the average neurite size. For ρ ≤ η, i.e., Δ ≤ *R*_*a*_ − *R*_*d*_, the dendrite is entirely inside the axon and the expected number of synapses is maximal. As the axon-dendrite distance increases further, the overlap between the two neurites decreases until it vanishes for ρ > 1, i.e., for Δ > *R*_*a*_ + *R*_*d*_.

Figures 4B–D show three typical results obtained for axons and dendrites modeled as truncated Gaussians. When neurite segments are evenly distributed across the neurite support, i.e., when the distribution variances are similar or larger than the neurite radii, the size of the axon-dendrite overlap dominantly determines the shape of distance-dependent expected number of synapses. The resulting function, shown in Figure 4B, is somewhat similar to the case obtained for the uniform density distributions from Figure 4A. For ρ ≤ η the function slowly decreases (unlike the case in Figure 4A where it is constant), while for ρ > η it decreases faster until it becomes zero. If one of the variances is similar to the average neurite size and the other is much smaller, the expected number of synapses behaves like an example in Figure 4C. The decrease from the maximal to zero value is much faster than in the case of Figure 4B. The presented example resembles a bell-shaped curve, but for some other model parameters the decrease can be even faster and result in a step function. The reason for this behavior is the following: one of the neurites has a small distribution variance, which means that the majority of neurite segments gets concentrated around the center of the neurite field. In this case, the increase in the distance between neurite centers decreases the distance-dependent expected number of synapses much faster than in the example in Figure 4B. When the neurite centers are close, the majority of neurite segments can form synapses, which gives maximal connectivity. For all axon-dendrite distances, when the neurite with small variance stays inside the area of other neurite, the number of synapses is high. But, when it approaches to the edge of the other neurite the majority of its segments becomes unavailable for creating synapses, so the expected number of synapses quickly decreases. If both the axon and dendrite have small variances, the expected number of synapses is a very narrow bell-shaped curve, as shown in Figure 4D. Both neurites have a majority of segments located around the neurite centers. As soon as those centers move apart, the probability of connection drops to almost zero. In this case, the neurite asymmetry index does not affect the expected number of synapses as much as in the other cases because narrow distributions effectively decrease neurite radii.

#### 3.1.2. The expected number of synapses as a function of the average neurite size (Figures 4E–H)

The relation between *S* and the average neurite size is examined by fixing all the parameters except *R*. The dependency is described by function ρ^2^ ϕ(ρ, η, *M*), named the size-dependent expected number of synapses, and illustrated in the right column in Figures 4E–H. The x axis shows the inverse of the normalized axon-dendrite distance on the interval $\frac{1}{\rho }\in [1,+\infty )$ and the average neurite radius on the interval $R\in \left[\frac{\Delta }{2},+\infty \right)$. The same neurite distributions and the same values of the asymmetry index are considered as in Figures 4A–D.

The size-dependent expected number of synapses is determined by two opposing mechanisms. An increase in the average neurite size leads to an increasing overlap between the two neurites from zero (for $R=\frac{\Delta }{2}$) to the maximal overlap containing the entire dendrite field (for $R=\frac{\Delta }{2\eta }$). The increasing overlap leads to the increasing expected number of synapses. At the same time, the increase in the average neurite radius leads to a decrease in the normalized axon-dendrite distance, the variable that reflects the distribution of neurite segments. As the neurite size increases, the fixed number of segments gets distributed over a larger area, so that the probability of neurite segment per unit area decreases. Eventually, this probability approaches zero as the average neurite size becomes very big. Clearly, the smaller probability of finding two neurite segments within the same unit area decreases the expected number of potential synapses. For small values of the average neurite size, the first effect is dominant and the expected number of synapses increases with *R*. For the larger neurites the second effect dominates and the expected number of synapses decreases with the increasing *R*. The same arguments hold for all the neurite distributions that we examined which is illustrated in Figures 4E–H.

#### 3.1.3. Properties of the distance-dependent expected number of synapses (Figure 5)

**Figure 5:**
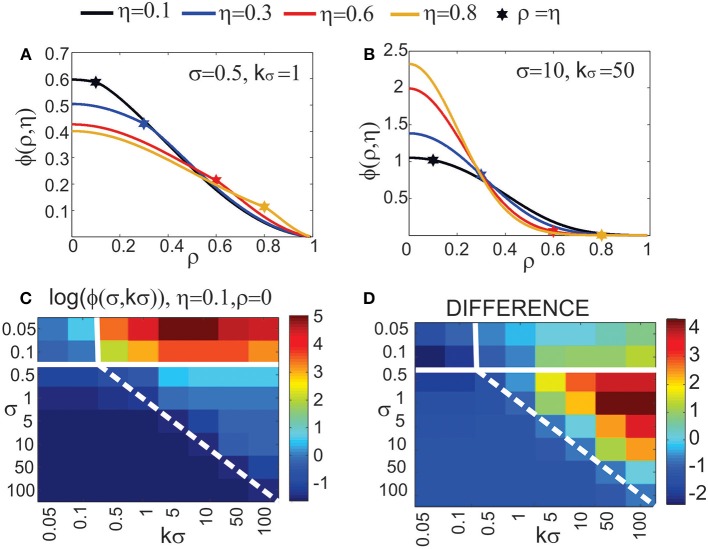
**Top row:** Additional analysis of the distance-dependent expected number of synapses [ϕ(ρ, η, *M*)]. The point where ρ = η is marked with a star. **(A)** Truncated Gaussian distribution of neurites with parameters σ = 0.5 and *k*_σ_ = 1 (repeated example from Figure 4B). **(B)** Truncated Gaussian distribution of neurites with parameters σ = 10, *k*_σ_ = 50 (repeated example from Figure 4C). **Bottom row:** Maximal values of the distance-dependent expected number of synapses, obtained for ρ = 0. **(C)** shows the logarithm of the maximal values obtained for η = 0.1, ρ = 0 and a wide range of values for σ and *k*_σ_. **(D)** illustrates the range of values for the logarithm of the function maxima, i.e., the difference between log(ϕ) for η = 0.8 and for η = 0.1. Bars on the right of the panels show the color code. The values for σ and *k*_σ_ are given on the y and x axis, respectively. The white lines on the panels divide the parameter space (σ, *k*_σ_) according to the shape of the obtained function ϕ(ρ, η, *M*). The upper left area and the lower right triangular area give functions between step-functions and bell-shaped curves, as in **(B)**. The upper right area gives narrow bell-shaped functions like the ones from Figure 4D. The lower left area corresponds to functions similar to the case of neurites with uniform distribution, shown in **(A)**. The dashed white line indicates a slow transition of the function shape between the two areas.

Two additional aspects of the distance-dependent expected number of synapses should be analyzed for the truncated Gaussian neurites, its maximal value obtained when the axon and dendrite centers overlap (ρ = 0) and the value obtained when the axon and dendrite edges touch from the inside (ρ = η). When the axon-dendrite overlap is maximal (for ρ ≤ η), the expected number of synapses slowly decreases as the distance between the neurite centers increases, but when the overlap is smaller than the maximum (for ρ > η) the decrease becomes faster. The point of change is marked with a star in Figures 5A,B, which are the repeated examples from Figures 4B,C. The neurites in Figure 5A have more evenly distributed neurite segments so the size of the axon-dendrite overlap has a bigger effect on the expected number of synapses and the shape of the function ϕ(ρ, η, *M*). For the truncated Gaussian neurites, the function ϕ(ρ, η, *M*) is always invertible and the effective radius can be computed (see Equation 5). The situation is different for neurites with the uniform distribution of segments, where the point (ρ = η) marks the transition from the constant to the monotonously decreasing part of the function. The constant segment is not invertible, therefore we consider only the monotonously decreasing part, i.e., the function obtained for ρ > η.

Figures 5C,D illustrate the range of maximal values for the distance-dependent expected number of synapses, obtained when the two neurites overlap maximally. For the truncated Gaussian neurites the maximal overlap is also given by the following equation obtained for ρ = 0 (see Supplementary Material 1):

(25) $$\begin{array}{l} \phi_{max}(\eta ,M)=\phi (0,\eta ,M)=\frac{k_{\sigma }^{2}}{1+k_{\sigma }^{2}}\cdot \frac{\pi }{8\sigma^{2}}\\ \;\cdot \frac{1-\exp\left(-\frac{{\left(1-\eta \right)}^{2}\cdot (k_{\sigma }^{2}+1)}{8\sigma^{2}}\right)}{\left(1-\exp\left(-\frac{{\left(1+\eta \right)}^{2}k_{\sigma }^{2}}{2\sigma^{2}}\right)\right)\cdot \left(1-\exp\left(-\frac{{\left(1-\eta \right)}^{2}}{2\sigma^{2}}\right)\right)}. \end{array}$$

Figure 5C shows log(ϕ_*max*_) for the asymmetry index η = 0.1 and a wide range of values for σ and *k*_σ_, the logarithm is used because the function varies a lot for the given range of parameters. Figure 5D illustrates the range of values for log(ϕ_*max*_) obtained for different asymmetry indices, i.e., it shows the difference between log(ϕ_*max*_) obtained for η = 0.8 and for η = 0.1. Blue areas in Figure 5D correspond to the cases when the distance-dependent expected number of synapses decreases with the increase of the asymmetry index. The white lines that parcel the parameter space (*k*_σ_, σ) mark the regions that give different types of functions. The upper left region corresponds to narrow dendrites, wider axons and the distance-dependent expected number of synapses in the form that goes from a step-functions to a bell-shaped function. The upper right region with high amplitudes corresponds to narrow axons and dendrites that give narrow bell-shaped expected number of synapses show in Figure 4D. The lower left region marks the parameter space that gives functions similar to those obtained for the uniformly distributed neurites, examples are shown in Figure 4B and in Figure 5A. The lower right triangular region corresponds to narrow axons and wider dendrites and the expected number of synapses in the form that goes from step-like to bell-shaped functions. In this case, the function maximum depends a lot on the asymmetry index, as indicated by the large values in Figure 5D. As the asymmetry index increases the size of the dendrite decreases compared to the axon size, the dendrite segments become more concentrated in a small area around the center which increases the probability of forming a synapse. The example in Figure 4C and in Figure 5B is picked near the border between the two regions, close to the dashed white line. The dashed line indicates a gradual transition between the regions^5^.

### 3.2. Effective radius and node degree (Figure 6)

**Figure 6:**
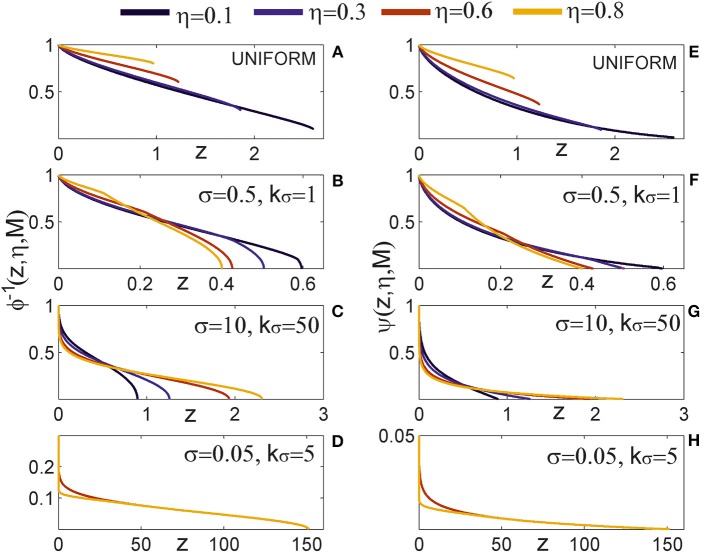
**Effective radius and node degree. Left column:** The inverse function of the distance-dependent expected number of synapses [ϕ^−1^(*z*, η, *M*)] which maps the non-linear dependency between the effective radius and the model parameters. The x axis shows the function argument $z=\frac{R^{2}\pi }{2N_{a}N_{d}D^{2}}$ which takes values from the interval [0, ϕ_*max*_]. **Right column:** The node degree is a quadratic function of the effective radius in the considered model. It depends on the square of the inverse distance-dependent expected number of synapses, ψ(*z*, η, *M*) = [ϕ^−1^(*z*, η, *M*)]^2^, which is shown in panels in the right column. The x axis in these panels also shows the variable *z* on the interval [0, ϕ_*max*_]. The effective radius and the node degree are shown for the same model parameters as the examples in Figures 4, 5. The asymmetry index takes the values η ∈ {0.1, 0.3, 0.6, 0.8}. The distribution of neurite segments is either uniform or truncated Gaussian with parameters σ = 0.5, *k*_σ_ = 1), (σ = 10, *k*_σ_ = 50) or (σ = 0.50, *k*_σ_ = 5).

The effective radius Δ_*max*_ is the maximal distance between an axon-dendrite pair expected to connect with at least one synapse and is given by Equation (5). In this section we analyze the properties of the inverse distance-dependent expected number of synapses (ϕ^−1^) that maps the non-linear dependency between the effective radius and the model parameters. Figure 6 shows how this function depends on variable *z* that is related to several model parameters as $z=\frac{\pi }{2N_{a}N_{d}D^{2}}\cdot R^{2}$. The range of values for *z* is determined from Equation (5) and is equal to

$$\begin{array}{l} 1\leq \frac{1}{z}\phi (\rho ,\eta ,M)\leq \frac{1}{z}\phi_{max}(\eta ,M)\\ \Rightarrow z\leq \phi_{max}(\eta ,M)=\phi (0,\eta ,M). \end{array}$$

The inverse distance-dependent expected number of synapses, and consequently the effective radius, exists on the interval *z* ∈ [0, ϕ_*max*_] for every distribution that we analyzed in this work. Figure 6A shows the case with uniform distribution of neurite segments where the function almost linearly decreases from one (for *z* = 0) to η$\left(\text{for }z=\frac{\pi }{{\left(1+\eta \right)}^{2}}\right)$. Figures 6B–D illustrate examples with a truncated Gaussian distribution of neurite segments (the same examples are shown in Figures 4, 5). In all those examples the effective radius decreases with *z*, but with non-linearities that are the most visible for *z* around zero and around the maximum. When *z* increases the ratio $\frac{N_{a}N_{d}}{R^{2}}$ decreases. This ratio is proportional to the average number of axon-dendrite pairs of segments per unit area. A decrease of $\frac{N_{a}N_{d}}{R^{2}}$ decreases the expected number of synapses everywhere, and further from the neurite center the expected number of synapses can decrease below one. Consequently the effective radius becomes smaller.

The node degree is a quadratic function of the effective radius (see Equation 6) described by the function ψ(*z*, η, *M*), which is the square of the inverse distance-dependent expected number of synapses. This function is shown in Figures 6E–H for the same examples as those in Figures 6A–D^6^.

### 3.3. Motif distribution

Equation (12) for 2-node motifs and Equations (16, 17) for 3-node motifs were numerically integrated using the Matlab built-in function quad2d ^7^. The exception is the innermost integral in Equation (17) which was computed using the simple trapezoid method in order to increase the speed of computations. The obtained results were additionally verified by simulating the equivalent model in Matlab, then counting motifs from the simulations.

#### 3.3.1. The expected number of motifs (Figure 7)

**Figure 7:**
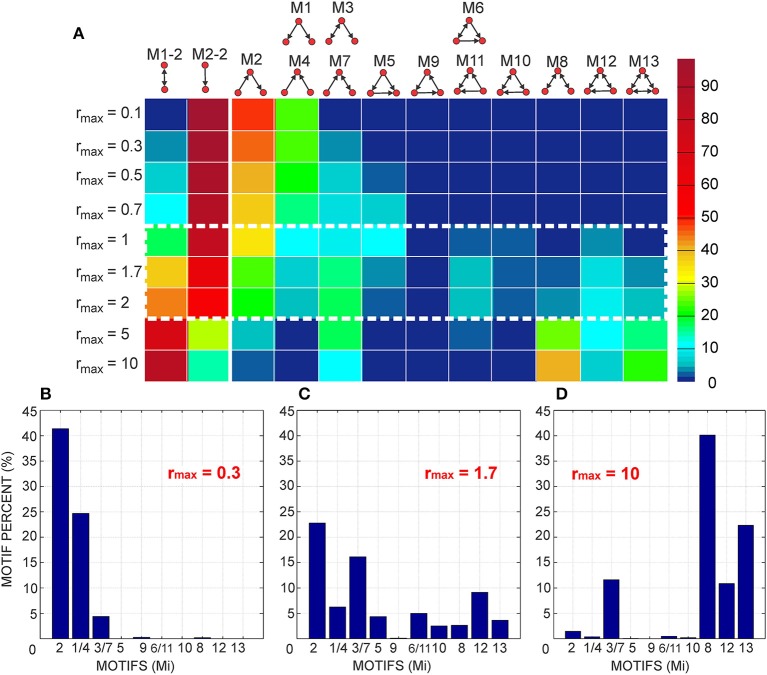
**The expected motif counts normalized with the total number of the same-size motifs. (A)** The expected motif counts obtained for a wide range of values for the normalized effective radius (*r*_*max*_ ∈ {0.1, 0.3, 0.5, 0.7, 1, 1.7, 2, 5, 10}). The color code on the right shows the percentages of different motifs in the total number of the same-size motifs. The left part of the panel (columns one and two) corresponds to the 2-node motifs, while the right part of the panel illustrates the 3-node motifs. The middle range of the values for *r*_*max*_ (encircled with the dashed white line) results in the biggest variation of motifs. **(B–D)** These panels additionally illustrate the three typical results shown in **(A)**. **(B)** shows the expected motif counts in networks with a small parameter *r*_*max*_ = 0.3, where motifs with a few connections are pronounced. **(C)** shows the example for *r*_*max*_ = 1.7, where all of the motifs, except M9, are present with at least few percents in the total motif count. **(D)** illustrates an example with the largest considered value for *r*_*max*_ = 10, where the motifs with bidirectional connections dominate.

Figure 7A summarizes the expected motif counts for all 2-node and 3-node motifs. Each column in the color-coded matrix corresponds to one motif, while each row corresponds to one value of the normalized effective radius (*r*_*max*_) obtained by dividing the effective radius (Δ_*max*_) with the axon-dendrite distance in a neuron (Δ_*ad*_). We consider a wide range of values for the normalized effective radius, *r*_max_ ∈ {0.1, 0.3, 0.5, 0.7, 1, 1.7, 2, 5, 10}. The schematic representation of each motif is plotted at the top of the corresponding column. The motifs that have identical expected counts are represented by the same column (e.g., M1 and M4). Each motif count is normalized with the total number of same-size motifs, i.e., M1-2 and M2-2 are divided with the total number of 2-node motifs and motifs M1–M13 are divided with the total number of 3-node motifs. Normalization removes parameters that act as multiplicative constants in the expressions for motif counts, i.e., it removes the coefficient $\frac{\Delta_{ad}^{2}}{l^{2}}$ for the 2-node motifs and $\frac{\Delta_{ad}^{4}}{l^{4}}$ for the 3-node motifs. The normalized expected motif counts depend only on the normalized effective radius.

The first two columns in the color-coded matrix correspond to the 2-node motifs. For the smaller values of the normalized effective radius (*r*_*max*_ ≤ 2) most of the connections are unidirectional, as indicated by the higher percent of motifs M2-2 in the second column. For the two biggest values of the normalized effective radius most of the connections become bidirectional and the fraction of motif M1-2 increases over 50%.

The 3-node motifs are shown in columns 3–15, arranged according to the increasing number of connections (in one direction, i.e., a bidirectional coupling counts twice). For the smallest values of *r*_*max*_ the motifs with two unidirectional connections are dominant. As the parameter increases the percent of the motifs with one bidirectional and one unidirectional connection (M3 and M7) increases. The middle range of values for the normalized effective radius (*r*_*max*_ between 1 and 2, encircled with the dashed white line in the figure) is the most interesting as it gives the biggest variability of motif counts. For these values, almost all of the motifs are present in the network structure. For the biggest values of *r*_*max*_, most of the nodes form bidirectional connections and the motifs with bidirectional couplings become dominant. It should be noted that motifs M3 and M7 appear for all values of the normalized effective radius but the smallest one. They contain one bidirectional connection and one unidirectional connection between the three-nodes and seem to be the most feasible connectivity pattern for the considered type of network (with uniformly distributed and randomly oriented neurons). On the contrary, the cyclic pattern of motif M9 almost never appears in these networks.

These conclusions are additionally illustrated in Figures 7B–D, which show the motif percents for the three representative values of the normalized effective radius. Figure 7B shows the case for *r*_*max*_ = 0.3 when the motifs with a small number of unidirectional connections (M2, M1, and M4) dominate. Figure 7C illustrates the middle range of values for the normalized effective radius (example: *r*_*max*_ = 1.7), which enables the biggest variability of motifs. Figure 7D shows the case for the biggest *r*_*max*_ when the motifs with bidirectional connections dominate.

#### 3.3.2. Comparison with the uniform random and the locally coupled networks (Figure 8)

For a comparison, motif counts are computed for the uniform random and for the locally coupled networks described in Methods Section. The networks are simulated for *N* = 3600 nodes. Node degrees are computed according to Equation (6). The values of the normalized effective radius are the same as those considered in Figure 7, i.e., *r*_*max*_ ∈ {0.1, 0.3, 0.5, 0.7, 1, 1.7, 2, 5, 10}. The axon-dendrite distance in a neuron is fixed to Δ_*ad*_ = 1, and the parameter that determines the neuron density is *l* ∈ {0.3, 0.5}. For *l* = 0.3, the square of edge Δ*_ad_* contains about 11 somata (a denser network). For *l* = 0.5 that square contains 4 somata (a sparser network). For each value of the node degree we generated a uniform random network, strictly locally coupled network, and a locally coupled network with 10% of non-local connections. The construction of these networks is described in Methods Section. Each connection in the uniform random network is established with equal probability (that depends on the selected node degree) and independently of other connections. In the strictly locally coupled network, each node is connected to all the nodes within its connectivity area, which results in all bi-directional connections. The second example of the locally connected network is similar to the first one, but 10% of all the connections are removed and re-established with the closest nodes outside of the connectivity area.

**Figure 8:**
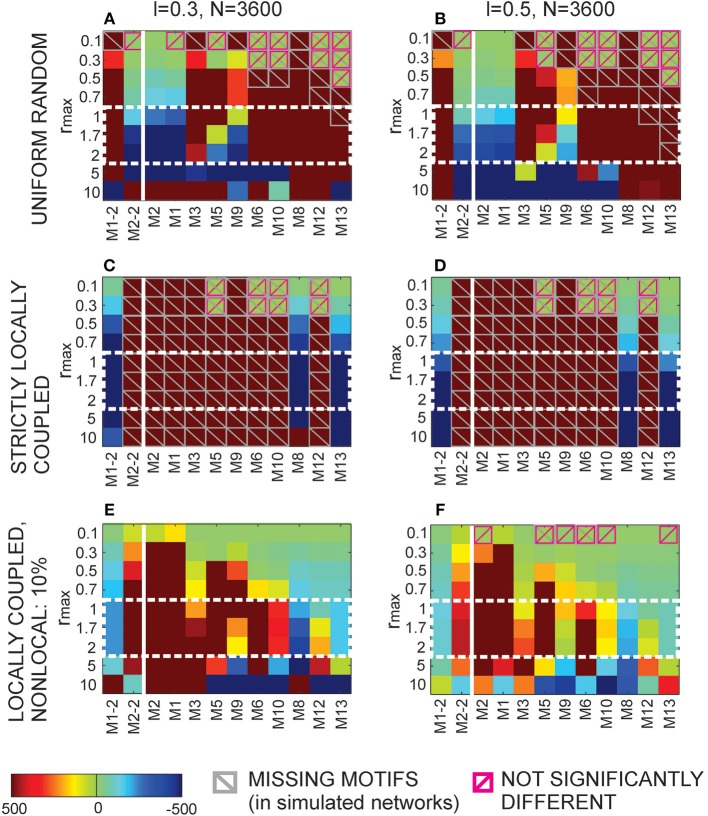
**Comparison of the results from Figure 7 with the motif counts obtained from simulated uniform random and locally coupled networks**. The color maps show the t-scores for all motifs (shown on the x axis) and all considered values of the normalized effective radius (shown on the y axis). The comparison is done for two population densities, a denser one (*l* = 0.3, left column) and a sparser one (*l* = 0.5, right column). A positive t-score indicates a bigger expected number of motifs in our model than in the simulated network, while a negative t-score indicates a smaller expected number in our model than in the simulated network. All the values outside the interval [-500, 500] are set to ± 500. The scores are shown as color maps in the same format as Figure 7, while the color bar at the bottom explains the color code. The crossed gray squares correspond to zero motif counts in the simulated networks, i.e., both the mean value and the variance are zero. The crossed pink squares are the cases when our model and the simulated networks give statistically the same results (the *t*-test with 5% significance level). **(A,B)** Comparison with the uniform random networks. **(C,D)** Comparison with the strictly locally coupled networks. In these networks all connections are bidirectional and only motifs M1-2, M8, and M13 are possible. **(E,F)** Comparison with the locally coupled networks with 10% non-local connections.

Figure 8 shows the comparison between our model and the simulated uniform random and the locally coupled networks. The color maps show t-scores computed using Matlab function ttest.m. For a simulated network, we obtained motif counts for every node (3600 values) and tested whether this sample has a mean value statistically equal to the expected motif count obtained from our model. The cases that pass the test are marked with the crossed pink squares in the figure. Clearly, most of the cases have significantly different motif counts than our model. The positive t-scores indicate that our model gives more motifs of a certain type than the simulated network, while the negative scores indicate fewer motifs in our model compared to the simulated network (t-scores obtained from Matlab are multiplied with -1). Also, we set the values outside of the interval [−500, 500] to ± 500, to emphasize the values closer to zero. In some cases, certain motifs do not appear in the simulated network. We marked them with gray crossed squares in the figure. When our model gives zero expected number of motifs, the case is marked with both gray and pink squares.

The color maps in Figure 8 are in the same format as in Figure 7. The color bar at the bottom of the figure explains the color code. The motif types are indicated on the x axis, and the values of the normalized effective radius are indicated on the y axis. The first row (Figures 8A,B) shows the comparison with the uniform random networks, the second row (Figures 8C,D) is the comparison with strictly locally coupled networks and the third row (Figures 8E–F) is the comparison with locally coupled networks with 10% non-local connections. The first column corresponds to the denser population (*l* = 0.3), and the second column to the sparser population (*l* = 0.5).

For almost all the cases shown in Figure 8, the number of bidirectional motifs (M1-2) in our model is larger than in the uniform random networks and smaller than in the locally coupled networks, while the opposite holds for the unidirectional motifs (M2-2). Similarly, the number of 3-node motifs with two unidirectional connections (M1 and M2) is smaller in our model than in the uniform random networks, and larger than in the locally coupled networks. On the contrary, the motifs with solely bidirectional connections (M8 and M13) are almost always more frequent than in the uniform random networks and less frequent than in the locally coupled networks. The motifs with three or four connections are in most cases more frequent in our model than in both the uniform random and the locally coupled networks. The exceptions are motifs M5 and M9 that become less frequent than in uniform random networks for a sufficiently big *r*_*max*_. As the normalized effective radius increases, our model forms more bidirectional connections and the motifs that require three unidirectional connections becomes less likely (this is more visible for M9, as it is anyway rare in our model). For even higher values of the effective radius, motif M10, which is not very frequent in our model, becomes underrepresented compared to both types of networks.

For the expected node degree of approximately 25–35% (cases: *r*_*max*_ = 5, *l* = 0.3 and *r*_*max*_ = 10, *l* = 0.5), our model contains dense local connectivity with many bidirectional connections. Eventually, most of the 3-node motifs become less frequent than in the uniform random networks except the three highly connected motifs, M8, M12, and M13. The motif M13 becomes more represented than in the locally coupled networks with 10% non-local connections, indicating very dense local connectivity in our model for these values of model parameters. On the contrary, motif M8 with two bidirectional connections is always less frequent in our model than in the locally coupled networks.

The last set of model parameters, *r*_*max*_ = 10, *l* = 0.3, gives very high connectivity, the probability of connection reaches 0.97 in the network with 3600 nodes. The obtained results are not consistent with the rest of the analysis, as in this case both simulated networks contain a high number of the most connected motif M13, while many other motif types become less frequent than in our model. We wanted to show this case to illustrate the effect of the finite simulation size. Our model allows analysis for any value of the model parameters, but in the simulated networks, the model size determines the maximal range of feasible parameters.

### 3.4. Clustering coefficient, path length and small-world coefficient (Figure 9)

Once the motif counts are obtained, the clustering coefficient follows from Equation (19). For comparison, we also evaluated the clustering coefficient for the uniform random and for the locally coupled networks with 10% of non-local connections (see Methods Section). The clustering coefficients are computed from the motif counts. Motifs in random and locally coupled networks are computed in a standard way, by counting the connectivity patterns. Those counts are used in Equation (19) instead of 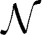
_*Mi*_ values. The motifs are multiplied with the coefficients 1 for M5 and M9, 2 for M6, M10, and M11, 4 for M12, and 8 for M13 in the numerator of the equation in order to take into account bidirectional connections in some of the motifs, the same way as in the standard expression for the clustering coefficient (Equation 18).

**Figure 9:**
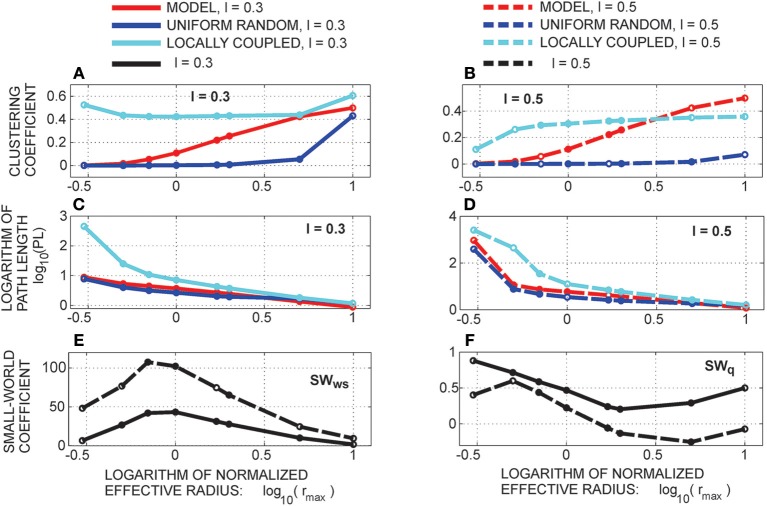
**The clustering coefficient (A,B), 10-based logarithm of the harmonic path length (C,D), and two definitions of the small-world coefficient (E,F). (E)** shows the standard definition (Watts and Strogatz, 1998) and **(F)** the alternative definition introduced in Telesford et al. (2011). Uniform random and locally coupled networks used for comparison are simulated for 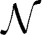 = 4900 neurons and model parameters Δ_*ad*_ = 1, *l* = 0.3 (solid line) and *l* = 0.5 (dashed line). Red line, our model; blue, uniform random networks; turquoise, locally coupled networks with 10% of non-local connections. The x axis shows the logarithm of 10 of the normalized effective radius (*r*_*max*_), y axis gives the considered network measures or their logarithms.

The simulated random and locally coupled networks have 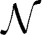 = 3600 or 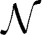 = 4900 nodes and model parameters Δ_*ad*_ = 1 and *l* ∈ {0.3, 0.5}. We consider only values from 0.3 to 10 for the normalized effective radius. For *r*_*max*_ = 0.1 the obtained networks are sparsely connected, possibly with many isolated cells. This causes a bias in the computation of the clustering coefficient and we omit these examples.

Figures 9A,B show the clustering coefficient for our model (red line), and also for the uniform random (blue) and the locally coupled network (turquoise) for two populations, a denser one (for *l* = 0.3) and a sparser one (*l* = 0.5). For most of the values of the normalized effective radius (*r*_*max*_), the clustering coefficient of our model is in between those of uniform random and locally coupled networks. Only for the largest two values of *r*_*max*_ in the sparser population the clustering coefficient becomes bigger than the one in the locally coupled networks. For small values of *r*_*max*_, axon and dendrite centers are relatively far apart and connect with different groups of cells. As *r*_*max*_ increases the axon and dendrite centers approach each other and the cells that connect to the axon become closer to those that connect to the dendrite, which makes connections between them more probable. This increases the number of motifs that contribute to the clustering coefficient. The distance between the axon and dendrite increases the effective area of the neighborhood, which might be a reason for the cases with higher clustering coefficient than in locally coupled networks. In the locally coupled networks, the dendrite and axon centers are identical and the neighborhood is defined by a single circle around that center. It should be noted that (Rieubland et al., 2014) reports higher clustering coefficient estimated from the experimental data than the one computed from the simulated uniform random and locally coupled networks. The example obtained for *l* = 0.3 and *r*_*max*_ = 10 demonstrates that the cut-off effect present in smaller networks (see Figure 8 obtained for 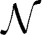 = 3600) disappears when comparing our model with bigger simulated networks (for 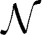 = 4900). An extensive comparison between our “infinite-size” model and the finite size simulated networks is presented in Supplementary Material 3.

The expected harmonic path length obtained using the iterative (Equations 20–23) (see Methods) is shown in Figures 9C,D. For all the considered model parameters the harmonic path length is slightly bigger in our model than in the uniform random network and smaller than in the locally coupled network. The computations used in this study result in somewhat smaller values for the harmonic path length than those obtained when simulating the equivalent model. This is a consequence of the finite simulation size (see the analysis presented in Supplementary Material 3). Consequently, the harmonic path length obtained from the numerical simulations differs more from the harmonic path length in the uniform random network, but is still smaller than the harmonic path length in the locally coupled network.

Finally, we computed the small-world coefficients out of clustering coefficients and path lengths. Two definitions of this coefficient are computed, the standard Watts-Strogatz definition (*SW*_*ws*_, see Watts and Strogatz, 1998), shown in Figure 9E, and an alternative definition *SW*_*q*_ from Telesford et al. (2011), shown in Figure 9F. The standard version compares our model to the uniform random networks and should be large for small-world networks. The alternative definition compares our model to both, the uniform random and the locally coupled networks, and should be around zero for the small-world networks. Both considered populations (for *l* = 0.3 and *l* = 0.5) maximize *SW*_*ws*_ for the normalized effective radius *r*_*max*_ = 0.7. The alternative coefficient *SW*_*q*_ is the closest to zero for *r*_*max*_ = 1.7 and *r*_*max*_ = 2, although, for the denser population (*l* = 0.3) it stays above zero for all the values of *r*_*max*_. The parameter *r*_*max*_ in the interval [1, 2] also maximizes the repertoire of possible motif counts, as shown in Figure 7.

## 4. Discussion

We presented a two-level statistical model that examines how properties of single neurons and neurites constraint the connectivity in neuronal population. The connectivity is quantified using the standard graph theoretic measures like motif counts, clustering coefficient, harmonic path length, and the two definitions of small-world coefficient. Neurites are represented as neurite fields in accordance with the model already addressed in the literature (Snider et al., 2010; Teeter and Stevens, 2011; Cuntz, 2012; van Pelt and van Ooyen, 2013; McAssey et al., 2014). Such model provides a low-resolution and low-dimensional representation of neurites. The entire neuron model has three components, the neurite field of the axon, the neurite field of the dendrite, and the parameter that maps the distance between the axon and dendrite centers. The population of neurons is uniformly distributed in two-dimensional space with the density of neurons defined by a model parameter. This resembles the experiments with dissociated cortical cultures, and is often used in theoretical studies. Finally, the synapse formation rule is entirely based on the proximity of axons and dendrites (Peters' rule, Peters et al., 1991; Peters and Feldman, 1976), and no activity-dependent synapse reorganization is considered. Consequently, we consider only the potential connectivity as defined in Stepanyants and Chklovskii (2005). The synapse formation rule, as well as the population properties, are selected to emphasize the role of neuron morphology and make a clear link between the morphology and connectivity.

### 4.1. Summary of the findings

We first introduced the notion of effective radius of neurites, which is the maximal distance between an axon-dendrite pair of two neurons expected to connect with at least one synapse. The effective radius, the expected number of synapses, and the expected node degree are expressed as functions of neurite parameters. The expected number of synapses linearly depends on the density of neurite distribution, but non-linearly on the neurite size and the distance between the axon and dendrite centers. We considered several choices of neurite distributions, including the uniform distribution and several cases of the truncated Gaussian distribution with different distribution parameters. When both axon and dendrite are evenly distributed within the distribution support the expected number of synapses decreases almost linearly with the axon-dendrite distance. This has also been observed in the experimental studies (Rieubland et al., 2014), and in the modeling studies that reproduce realistic neuronal morphologies (Hill et al., 2012).

Next, we expressed the considered connectivity measures as functions of the normalized effective radius, which is the effective radius divided by the distance between axon and dendrite centers of the same neuron. We derived the closed-form expressions for the 2- and 3-node motifs. These motifs represent the minimal-size networks with structured connectivity that can be studied experimentally. The experimental study of path lengths requires recording of a much bigger population of neurons, which can easily become infeasible. The expected motif counts are expressed in the form of multiple integrals that are evaluated numerically. The obtained results vary significantly for different values of the normalized effective radius. For most of the considered values of the normalized effective radius, the unidirectional 2-node motifs are more frequent than the bidirectional motifs. This resembles the statistics of 2-node motifs in the uniform random networks. For large values of the normalized effective radius, the bidirectional motifs become dominant, similarly as in the locally coupled networks. Additional comparison shows that our model always expresses more bidirectional motifs that the uniform random networks and less than the locally coupled networks. The opposite holds for the unidirectional motifs.

The sparsely connected 3-node motifs (with 2 unidirectional connections) are dominant for the small normalized effective radius, which resembles the 3-node motif distribution in the uniform random networks. For the large normalized effective radius, the densely connected motifs (with two or three bidirectional connections) become frequent, which is typical for the locally coupled networks. For all considered values of the normalized effective radius, our model exhibits less sparsely connected motifs than the uniform random networks and more than the locally coupled networks. The opposite holds for the motif with the maximal connectivity (i.e., with three bidirectional connections). In-between these extremes we can identify the range of values for the normalized effective radius that maximizes the variability in connection repertoires on the micro-scale. For these values, almost all the motifs are present in the network, which is not the case in the uniform random and the locally coupled networks that favor certain motifs. The analysis of the clustering coefficient, harmonic path length, and the small-world coefficient shows that the same range of values results in the small-world coefficient closest to the one of the small-world networks. For the normalized effective radius between 1 and 2, the clustering coefficient is close to the one of the locally coupled networks, and the path length is somewhat longer than the one of the uniform random networks.

### 4.2. Axons and dendrites modeled as neurite fields

We adopted several approximations when choosing models for the individual neurons and for the populations of neurons. In what follows we will additionally motivate the adopted choices. The coarse representation of neurites, reduced to the distribution of neurite segments, neglects the fine details of the neurite tree structure, including the non-random orientation of neurite segments, the branching patterns, or any correlation in the structure of neurite branches. Previous studies suggest that this low-resolution neurite description still captures relevant dendrite properties at the level of the whole neuron morphology (Snider et al., 2010; Teeter and Stevens, 2011). In this work, we also used density fields to represent axons, which better describes the properties of neurons in cell cultures than in the three-dimensional tissue. In the cortical tissue, the axons are elongated and branched structures that cover large area compared to dendrites. In most of the cases, just a single axonal branch passes through the dendritic field (Braitenberg and Schüz, 1998). The axon density field can be interpreted as uncertainty of the position of individual axonal branches within the space covered by the axon. This complies with our model, where the principal axonal orientation is random, and the neurite field describes the additional randomness of position of the axonal branches with respect to the principal orientation. In the systems with non-random principal orientation of axons, or in neurons for which the correlation between the axonal branches cannot be approximated a model that describes each branch might be more suitable. For example, a neurite field description of dendrites can be combined with axons modeled in NETMORPH (Koene et al., 2009). Still, as long as both dendrites and axons cover a limited space, the effective radius can be derived as well as the expressions for the considered measures of network connectivity. Eventually, the expression for the effective radius might have more complex dependency on the neurite properties.

### 4.3. Potential synapses estimated from the neurite fields

An important issue related to this modeling approach is addressed in van Pelt and van Ooyen (2013). This study systematically examines several aspects of connectivity, including the number of synapses per neurite, the number of synapses between pairs of neurons, and the connectivity per neuron. Those aspects are evaluated for neurites with realistic branching trees and also for neurites described by the neurite density distribution. The paper finds agreement between realistic and neurite field based descriptions of neurons when estimating the expected number of synapses. But, the disagreement arises when computing the expected number of synapses per connected axon-dendrite pair. To overcome the problem, the authors proposed an empirical mapping function between the connectivity obtained from detailed simulated morphologies and the connectivity computed using density fields obtained by averaging over detailed simulated morphologies.

The model examined in our study derives the average connectivity from neurite distributions, therefore might suffer from the issues indicated in van Pelt and van Ooyen (2013). We can adopt the same method to overcome the problem, and apply an empirical mapping function to the Equation (5) that defines the effective radius. On the right side of the first inequality, instead of one there will be a constant dependent on the empirical mapping function. This constant will be added to the expression for the effective radius, but the rest of the analysis will not be affected. Eventually, the optimal range of values for the normalized effective radius might be shifted from the interval [1, 2]. Alternatively, the expected number of synapses can be obtained from a more realistic model of neurites, e.g., from the reconstructed cells from neuroimaging studies or from detailed morphologies simulated using NETMORPH (Koene et al., 2009). As long as the obtained function is at least piecewise invertible the effective radius can be computed from it, and the results for the expected motif counts and for the other considered measures still apply.

### 4.4. Relation between the actual and the potential number of synapses

We derive all the network measures from the potential connectivity, but potential connectivity does not fully explain the actual connectivity. The obtained potential number of synapses (Figure 4), the range of values and the functional form, is in agreement with other studies that estimate the connectivity from neuronal morphology (Hill et al., 2012; van Pelt and van Ooyen, 2013), but it cannot fully explain the actual synaptic connectivity reported in Markram et al. (1997) and Fares and Stepanyants (2009). Figure 4 indicates the adequate range of values for the properly selected model parameters. The distance-dependent expected number of synapses [ϕ(ρ, η, *M*)] is smaller than 3 for the typical examples presented in Figure 4, with much bigger values obtained only for the very narrow (and unrealistic) neurite fields. To obtain the expected number of potential synapses, it should be multiplied with a coefficient that depends on the model parameters and is not greater than ϕ_*max*_. The obtained expected number of synapses per connection reaches 10 synapses or less. Although the range of values is (roughly) accurate for the properly selected model parameters, the distribution of synapse counts is not according to Fares and Stepanyants (2009). This study demonstrates that the distribution of potential synapses between a connected axon-dendrite pair has much higher variance than the distribution of actual synapses. They proposed a cooperative model of synapse formation, described by a sigmoid function, that establishes actual synapses only between axon-dendrite pairs with sufficient number of potential contacts, while it rules out the pairs with few contacts. This correction can be incorporated in our study, similarly to the mapping function discussed in the previous paragraph, by applying the proposed sigmoid function to the left side of the first inequality in Equation (5).

Finally, corrections proposed in van Pelt and van Ooyen (2013) and Fares and Stepanyants (2009) can be combined. First, the empirical mapping function from van Pelt and van Ooyen (2013) can be used to convert the synapse counts obtained from the neurite fields to the values that would be obtained by simulating detailed morphologies. Then, the cooperative rule from Fares and Stepanyants (2009) can be used to convert the number of potential synapses to the counts of actual synapses. All these operations will somewhat alter the functional form of Equation (5) and, consequently, the expression for the effective radius and how it depends on the neurite parameters. Eventually, an additional parameter might be introduced to describe the connectivity area. The computation of the network measures can then be done following the same method described in this study.

### 4.5. Alternative potential synapse formation rules

We considered a simple potential synapse formation rule based on the proximity criteria: axon and dendrite segments form contacts if they find themselves on a distance smaller than the average dendritic spine length. The only constraint is that a dendritic segment cannot form potential synapses with more than one segment of the same neighboring axon. Still it can form potential synapses with the segments of other axons. A more realistic rule would require that each dendritic segment connects to at most one among all the proximal segments of all the axons, this may better reflect the connectivity in cortical tissue (Braitenberg and Schüz, 1998) and also reduce the total number of potential synapses per neuron. In the current model, the number of potential synapses is controlled by the choice of model parameters (see Methods). An alternative potential synapse formation rule would allow a wider range of model parameters. Implementing the alternative rule would likely result in a more complex relation between the effective radius and the neurite parameters. Still, if we consider one particular dendrite, all the axons that connect to it have to be on a finite distance from it, and the effective radius is always finite. The alternative rule would alter the criterion for connectivity: a neuron would not connect to all the neurons inside of its connectivity area, but just to some of them and according to some selection criteria derived from the potential synapse formation rule.

Activity-dependent synaptic rearrangement is not considered in this study, although it represents an important mechanism in shaping the synaptic patterns. We focus on the most stable aspects of neuronal connectivity, those governed by morphology of neurite trees. As indicated in the literature (Stepanyants et al., 2002), remodeling of neurite branches requires longer time scale than formation or removal of the individual synapses. The synaptic connectivity derived from neuromorphology can be considered as an additional constrain in the process of the activity-dependent synaptic rearrangement. It is reasonable to expect that the networks with larger diversity of motif counts retain larger variability of the connectivity also in the presence of the activity-dependent synaptic changes.

### 4.6. Comparison with the experimentally observed motif counts

The presented study focuses on a statistical description of neuronal connectivity and the constraints to connectivity imposed by low-resolution properties of neuronal morphology. The considered problem was solved analytically. We established the functional dependencies between the considered connectivity descriptors and the parameters that describe neuronal morphology and the organization of neuronal population. In order to solve the described problem, we had to approximate several mechanisms that significantly influence the formation and maintenance of synaptic contacts. Those include the details of neurite structure, the realistic organization of neurons in the cortical tissue (as we considered a model that corresponds to organization in cell cultures), and most importantly the fine tuning of connectivity patterns through synaptic plasticity. Consequently, certain differences between the results obtained from our model and the corresponding experimental findings are expected.

The studies in Markram et al. (1997), Song et al. (2005), and Perin et al. (2011) examined the connectivity between cortical layer 5 pyramidal neurons and reported over-representation of bidirectional motifs compared to the uniform random networks. The study in Rieubland et al. (2014) addressed the connectivity between molecular layer interneurons in the cerebellum and found no significant difference compared to the uniform random networks. In Markram et al. (1997), 30% observed connections were bidirectional and 70% unidirectional. This corresponds to distribution of unidirectional and bidirectional connections obtained in our model for the normalized effective radius close to 1.7. Our results show that values of the normalized effective radius smaller than 2 give less than 50% of bidirectional connections, while the opposite holds for the normalized effective radius larger than 2. For almost every choice of the model parameter value, the number of bidirectional connections exceeds the one of the uniform random networks, similarly as in Song et al. (2005) and Perin et al. (2011). A recent study (Cossell et al., 2015) examined the role of bidirectional connections in sensory information processing. They found that neurons with correlated responses to visual stimuli often connect with strong bidirectional couplings, while the majority of neurons exhibits weakly or uncorrelated responses to visual stimuli and connects with unidirectional couplings.

Three studies (Song et al., 2005; Perin et al., 2011; Rieubland et al., 2014) reported the distribution of 3-node motifs in cortical neuronal networks. In Song et al. (2005), the authors defined the optimal transitive connectivity rule stating that “if node *N*_1_ connects to *N*_2_, and *N*_2_ connects to *N*_3_ (in any direction), the probability that *N*_1_ connects to *N*_3_ significantly exceeds the chance level.” Motifs M1, M5, M6, M9, M10, M11, M12, and M13 have been found in the data more often than in the uniform random networks. In addition, motif M3 was less frequent than in the uniform random networks. The study in Perin et al. (2011) confirms the same connectivity rule and finds motifs M1, M5, M6, and M11 to be overrepresented in the data compared to the locally coupled networks. In Rieubland et al. (2014), the preference for transitive motifs is also confirmed, with motifs M1 and M5 being overrepresented compared to both the uniform random and the locally coupled networks. Our model suggests the optimal range of values for the normalized effective radius that supports formation of the reported motifs (particularly, M5, M6, M10–M13), i.e., the interval *r*_*max*_ ∈ [1, 2]. Outside of this interval, some of these motifs become rare. Contrary to Song et al. (2005), we rarely ever observe the loop-motif M9, the same motif is also rare in the locally coupled networks. Motif M12 becomes relatively frequent in our model for the sufficiently big values of *r*_*max*_. Although it is not reported in all experimental studies, it also has transitive connectivity. We frequently observe motifs M3 and M7, more frequently than in both the locally coupled and the uniform random networks. Such motifs can be formed between three neurons if two of them fall inside the connectivity area of the third one in such a way that one is close to the center of the connectivity area and the other is close to its border. The neuron close to the center of the connectivity area is likely to form a bidirectional connection present in motifs M3 and M7. The neuron close to the border of the connectivity area is likely to form the remaining unidirectional connection.

Finally, it should be mentioned that our model cannot predict missing connections and disconnected motifs, like those analyzed in Rieubland et al. (2014), or the anti-clustering coefficient emphasized in the same study. This is a consequence of the definition of connectivity area and the fact that all dendrites within the connectivity area of an axon synapse to that axon. A different synapse formation rule, allowing that some of the dendrites within the connectivity area remain disconnected from the considered axon, like the alternative rule described in a previous paragraph, would allow analysis of the missing connections and the additional motifs discussed in the literature.

### 4.7. Limitations of the experimental studies

Connectivity measures obtained from experimental studies are to some extent affected by the adopted experimental protocols. A recent modeling study (Miner and Triesch, 2014) examined the possible bias in the connectivity measures introduced by sampling and finite size of the slices. Our model can also be used to examine the effects of the finite size of the considered neuronal population. The analytical results presented in our study are derived for an infinite-size population of neurons. On contrary, simulation of the equivalent model can only be done for the finite number of neurons. Comparison between the analytical and the simulated results illustrates the bias induced when estimating the properties of a large neuronal circuit using a small sub-population. In the following paragraph, we give two examples of this issue. We illustrate a case where the finite network size affects motifs computation. We carefully discuss how the reduction of model size affects the path-length and the small-world coefficient computations.

### 4.8. The effects of the finite model size

In most of the derivations presented in this study, the network size is not explicitly considered, i.e., we treat the model as if it were infinite. An exception is the path length, a global measure of the network structure that has to depend on the model size. In our study, the information about model size is, however, introduced only in the later steps of the path length computation. (In)finite model size becomes an issue if we want to compare our model to a simulated, therefore, a finite-size network. In Figure 8, we compare the expected motif counts obtained from our model to those obtained from the uniform random and the locally coupled networks. The result shown for the biggest value of the effective radius is biased due to the finite number of neurons in the network. While our model does not suffer from this effect, the two simulated networks do. The large effective radius leads to a large number of neighbors, in the considered case those neighbors represent 97% of all the network nodes. Clearly, both the uniform random and the locally coupled networks become densely connected, close to all-to-all connectivity, so the results obtained in this case visibly deviate from all the other examples.

In our model, the harmonic path length is computed using the iterative equations derived in Methods Section. The obtained harmonic path length is somewhat smaller than the result computed by simulating the equivalent model. In Supplementary Material 3, we analyze steps in computation of the path length and identify all the differences between the analytically solved and the simulated model. We first compute all the intermediate steps and probabilities defined by the iterative procedure. Then, we calculate all those intermediate probabilities from the simulated model and compare them to the results of the iterative procedure. The finite size of the model imposes the maximal distance between any pair of neurons. As we approach this maximal distance, the intermediate probabilities computed from simulations converge to zero. On contrary, the intermediate probabilities obtained using the iterative method do not contain the information about the model size, but instead describe an infinite-size model. Next, we compute the distribution of path lengths from the intermediate probabilities. This is possible only if we cut-off the intermediate probabilities at the maximal allowed distance between a pair of neurons in the model, and therefore artificially introduce the model size. Consequently, for the larger values of the effective radius the probabilities obtained from the iterative equations drop faster than the same probabilities obtained from the simulations. The harmonic path length obtained from the iterative equations is somewhat smaller than the result of simulations.

The connectivity measures most affected by model size are the small-world coefficients. As the size of simulated networks increases, the small-world coefficient computed using the definition from Watts and Strogatz (1998) increases. At the same time, the coefficient from Telesford et al. (2011) decreases and becomes closer to zero. In both cases, larger analyzed networks are more likely to be classified as small-world networks. The impact of numerical methods, model size, and number of simulation iterations is discussed in Supplementary Material 3. It should also be noted that the two considered definitions of the small-world coefficient lead to somewhat different conclusions. While the coefficient from Telesford et al. (2011) suggests that networks obtained for the effective radius in the interval [1, 2] have the connectivity closest to the small-world networks, the definition from Watts and Strogatz (1998) points at smaller values of the effective radius, namely the interval [0.7, 1]. The interval [1, 2] also maximizes the diversity in the obtained expected motifs counts, so the results obtained using the coefficient from Telesford et al. (2011) better agree with the motifs analysis. At the other hand, this coefficient seems to be more sensitive to the methodology used to compute the harmonic path length, although both considered methods (our iterative method and the numerical simulations) give qualitatively similar results.

### 4.9. Related modeling studies

Two previous modeling studies, Herzog et al. (2007) and Voges et al. (2010), use a similar neuron description to address the same problem, i.e., how the coarse scale properties of neuronal morphology shape the connectivity in large networks. They examined a neuron model that reproduces patchy connections observed in the cortex. Axons are modeled as Gaussian neurite fields with the axon center displaced from the soma in order to capture the long-distance connectivity in the considered networks. A neuron is allowed to connect to other neurons close to its soma and also to the neurons close to its displaced axon field. The generated networks exhibit small-world properties suggesting optimized wiring in the cortex. In our model, both axons and dendrites are described by neurite fields, but axons can connect only to the dendrites that are sufficiently close to the axonal field. Our model is constructed to capture general properties of neuronal morphology suggested by Snider et al. (2010), while the studies in Herzog et al. (2007) and Voges et al. (2010) focus on the specific types of pyramidal cells with long patchy projections and the neuronal connectivity derived from this property. In a recent study (McAssey et al., 2014), a similar model that uses neurite density fields to represent axons and dendrites is analyzed through simulations. The authors carefully fitted the density fields using the reconstructed neuronal morphologies fed to the simulator (Koene et al., 2009). They demonstrated the realistic distribution of potential synapses and the optimal properties of the obtained networks treated as weighted graphs. The results suggest that these networks possess properties similar to the small-world networks.

The model we considered in this study is very similar to those described in Herzog et al. (2007), Voges et al. (2010), and McAssey et al. (2014), but we opted for a different approach to analyzing the model. Instead of simulating the model for different parameter sets, we derived the analytical solution that allows us to fully understand the significance of the individual model parameters. We introduced the concepts of effective radius and connectivity area. Through these concepts we mapped the parameters of the individual neurons to a combined parameter that further determines the network-level properties. Additional work should be done to estimate this parameter from the experimental data, an issue that will be a subject of our future studies.

The two-level statistical model analyzed in this study can be seen as a framework to connect single neuron properties with the network-level organization. The main question is how to reduce the number of parameters in the neuron model in order to easier embed it to the network-level model. Ideally, the single neuron model should include as much details as possible that are then reduced using averaging and statistical description into a lower-dimensional representation. The lower-dimensional representation should provide a possibility to clearly tract the most crucial aspects of the neuron model when embedded into the network. We followed this methodology by introducing the concept of effective radius. The adopted methodology provides flexibility in selection of model components and allows easier modification of the presented framework to include new aspects of neurons and neuronal populations.

### Conflict of interest statement

The authors declare that the research was conducted in the absence of any commercial or financial relationships that could be construed as a potential conflict of interest.

## Footnotes

^1^To simplify the explanations in the text we sometimes use the notation for neurite centers when talking about the corresponding neurites. For example, A1 could stand for “the center of the axon of neuron *N*_1_” but also for “the axon of neuron *N*_1_.” Since all neurons have equal properties, the only parameters that distinguish them are the coordinates of their centers.

^2^The notation *r* is simultaneously used for radial coordinates in the coordinate system of axon A1 and the axon-dendrite distances between A1 and other dendrites, because these distances are at the same time radial coordinates in the coordinate system of A1.

^3^The functions κ_1_ and κ are introduced as two separate notions in order to simplify the notation in the equations that follow.

^4^To following calculation confirms that all patterns are included in the table. Each pair of nodes forms one of the four types of connections (2 in one direction, 1 bidirectional, no connection), this gives 4^3^ = 64 motifs and 54 of them are connected motifs. In the table, some patterns repeat. Each motif M1, M4, and M8 corresponds to 3 different patterns. M2, M3, M5, M7, M10, and M12 correspond to 6 patterns each. M6 and M11 correspond to 3 different patterns, M9 to 2, and M13 to 1 pattern. This gives 54 patterns in total.

^5^Function ϕ(ρ, η, *M*) varies a lot with the model parameters. In order to ensure an accurate numerical evaluation of the function it has to be scaled down with a fixed coefficient before integration, then multiplied with the same coefficient after integration. Very small values of σ and large *k*_σ_ (very narrow Gaussians) might cause numerical errors even after the scaling (in the form of glitches for some values of ρ), which then requires additional scaling of the function. Anyway, such narrow Gaussians likely correspond to unrealistic morphologies.

^6^Function ϕ^−1^(*z*, η, *M*) is computed using a simple method. The values of ϕ(ρ, η, *M*) are computed on the interval [0, 1] with a resolution 0.001. For each value *z* the closest value of ϕ(ρ), ϕ(ρ_0_), is found. Then ϕ^−1^(*z*) is computed from the approximation $\phi (\rho )=\phi (\rho_{0})+(\phi^{-1}(z)-\rho_{0})\cdot \phi (\rho_{o}{)}^{\prime }\Rightarrow \phi^{-1}(z)=\rho_{0}+\;\frac{\phi (\rho )-\phi (\rho_{0})}{\phi^{\prime }(\rho_{0})}$. The derivative is also estimated using the simple equation $\phi^{\prime }(\rho +d\rho )=\frac{\phi (\rho +d\rho )-\phi (\rho s)}{d\rho }$.

^7^Matlab version R2014a

## References

[B1] Boccaletti S. Latora V. Moreno Y. Chavez M. Hwang D.-U. (2006). Complex networks: structure and dynamics. Phys. Rep. 424, 175–308. 10.1016/j.physrep.2005.10.009

[B2] Braitenberg V. Schüz A. (1998). Cortex: Statistics and Geometry of Neuronal Connectivity. Berlin; Heidelberg: Springer-Verlag.

[B3] Cossell L. Iacaruso M. Muir D. Houlton R. Sader E. Ko H.. (2015). Functional organization of excitatory synaptic strength in primary visual cortex. Nat. Lett. 518, 399–401. 10.1038/nature14182 25652823 PMC4843963

[B4] Cuntz H. (2012). The dendritic density field of a cortical pyramidal cell. Front. Neuroanat 6:2. 10.3389/fnana.2012.00002 22347169 PMC3269636

[B5] Deco G. Jirsa V. K. Robinson P. A. Breakspear M. Friston K. (2008). The dynamic brain: from spiking neurons to neural masses and cortical fields. PLoS Comput. Biol. 4:e1000092. 10.1371/journal.pcbi.1000092 18769680 PMC2519166

[B6] Fares T. Stepanyants A. (2009). Cooperative synapse formation in the neocortex. Proc. Natl. Acad. Sci. U.S.A. 106, 16463–16468. 10.1073/pnas.0813265106 19805321 PMC2738618

[B7] Frégnac Y. Rudolph M. Davison A. P. Destexhe A. (2007). Complexity in neuronal networks, chapter 9, in Biological Networks, ed Képès F. (Singapore: World Scientific), 291–340. 10.1142/9789812772367_0009

[B8] Herzog A. Kube K. Michaelis B. de Lima A. D. Voigt T. (2007). Displaced strategies optimize connectivity in neocortical networks. Neurocomputing 70, 1121–1129. 10.1016/j.neucom.2006.11.016

[B9] Hill S. L. Wang Y. Riachi I. Schürmann F. Markram H. (2012). Statistical connectivity provides a sufficient foundation for specific functional connectivity in neocortical neural microcircuits. Proc. Natl. Acad. Sci. U.S.A. 109, E2885–E2894. 10.1073/pnas.1202128109 22991468 PMC3479474

[B10] Koene R. A. Tijms B. van Hees P. Postma F. de Ridder A. Ramakers G. J.. (2009). Netmorph: a framework for the stochastic generation of large scale neuronal networks with realistic neuron morphologies. Neuroinformatics 7, 195–210. 10.1007/s12021-009-9052-3 19672726

[B11] Kriener B. Helias M. Aertsen A. Rotter S. (2009). Correlations in spiking neuronal networks with distance dependent connections. J. Comput. Neurosci. 27, 177–200. 10.1007/s10827-008-0135-1 19568923 PMC2731936

[B12] Liley D. Wright J. (1994). Intracortical connectivity of pyramidal and stellate cells: estimates of synaptic densities and coupling symmetry. Network. Comp. Neural. 5, 175–189.

[B13] Mäki-Marttunen T. Aćimović J. Nykter M. Kesseli J. Ruohonen K. Yli-Harja O.. (2011). Information diversity in structure and dynamics of simulated neuronal networks. Front. Comput. Neurosci. 5:26. 10.3389/fncom.2011.00026 21852970 PMC3151619

[B14] Mäki-Marttunen T. Aćimović J. Ruohonen K. Linne M.-L. (2013). Structure-dynamics relationship in bursting neuronal networks revealed using a prediction framework. PLoS ONE 8:e69373. 10.1371/journal.pone.0069373 23935998 PMC3723901

[B15] Markram H. Lübke J. Frotscher M. Roth A. Sakmann B. (1997). Physiology and anatomy of synaptic connections between thick tufted pyramidal neurones in the developing rat neocortex. J. Physiol. 500(Pt 2), 409–440. 9147328 10.1113/jphysiol.1997.sp022031 PMC1159394

[B16] McAssey M. P. Bijma F. Tarigan B. van Pelt J. van Ooyen A. de Gunst M. (2014). A morpho-density approach to estimating neural connectivity. PLoS ONE 9:e86526. 10.1371/journal.pone.0086526 24489738 PMC3906031

[B17] Milo R. Shen-Orr S. Itzkovitz S. Kashtan N. Chklovskii D. Alon U. (2002). Network motifs: simple building blocks of complex networks. Science 298, 824–827. 10.1126/science.298.5594.824 12399590

[B18] Miner D. Triesch J. (2014). Slicing, sampling, and distance-dependent effects affect network measures in simulated cortical circuit structures. Front. Neuroanat. 8:125. 10.3389/fnana.2014.00125 25414647 PMC4220704

[B19] Perin R. Berger T. Markram H. (2011). A synaptic organizing principle for cortical neuronal groups. Proc. Natl. Acad. Sci. U.S.A. 108, 5419–5424. 10.1073/pnas.1016051108 21383177 PMC3069183

[B20] Peters A. Feldman M. (1976). The projection of the lateral geniculate nucleus to area 17 of the rat cerebral cortex. I. general description. J. Neurocytol. 5, 63–84. 1249593 10.1007/BF01176183

[B21] Peters A. Palay S. L. Webster H. D. (1991). The Fine Structure of the Nervous System: Neurons and Their Supporting Cells. New York, NY: Oxford University Press.

[B22] Rieubland S. Roth A. Häusser M. (2014). Structured connectivity in cerebellar inhibitory networks. Neuron 81, 913–929. 10.1016/j.neuron.2013.12.029 24559679 PMC3988957

[B23] Snider J. Pillai A. Stevens C. F. (2010). A universal property of axonal and dendritic arbors. Neuron 66, 45–56. 10.1016/j.neuron.2010.02.013 20399728

[B24] Sompolinsky H. (2014). Computational neuroscience: beyond the local circuit. Curr. Opin. Neurobiol. 25, xiii–xviii. 10.1016/j.conb.2014.02.002 24602868

[B25] Song S. Sjöström P. J. Reigl M. Nelson S. Chklovskii D. B. (2005). Highly nonrandom features of synaptic connectivity in local cortical circuits. PLoS Biol. 3:e68. 10.1371/journal.pbio.0030068 15737062 PMC1054880

[B26] Sporns O. (2011). Networks of the Brain. Cambridge, MA: The MIT Press.

[B27] Stepanyants A. Chklovskii D. B. (2005). Neurogeometry and potential synaptic connectivity. Trends Neurosci. 28, 387–394. 10.1016/j.tins.2005.05.006 15935485

[B28] Stepanyants A. Hof P. R. Chklovskii D. B. (2002). Geometry and structural plasticity of synaptic connectivity. Neuron 34, 275–288. 10.1016/S0896-6273(02)00652-9 11970869

[B29] Teeter C. M. Stevens C. F. (2011). A general principle of neural arbor branch density. Curr. Biol. 21, 2105–2108. 10.1016/j.cub.2011.11.013 22169536

[B30] Telesford Q. K. Joyce K. E. Hayasaka S. Burdette J. H. Laurienti P. J. (2011). The ubiquity of small-word networks. Brain Connect. 1, 367–375. 10.1089/brain.2011.0038 22432451 PMC3604768

[B31] van Pelt J. van Ooyen A. (2013). Estimating neuronal connectivity from axonal and dendritic density fields. Front. Comput. Neurosci. 7:160. 10.3389/fncom.2013.00160 24324430 PMC3839411

[B32] Voges N. Guijarro C. Aertsen A. Rotter S. (2010). Models of cortical networks with long-range patchy projections. J. Comput. Neurosci. 28, 137–154. 10.1007/s10827-009-0193-z 19866352

[B33] Watts D. J. Strogatz S. H. (1998). Collective dynamics of ‘small-world’ networks. Nature 393, 440–442. 9623998 10.1038/30918

