# Supplementary material for: The effects of neuron morphology on graph theoretic measures of network connectivity: the analysis of a two-level statistical model
Source: Front Neuroanat. 2015 Jun 10;9:76. doi: 10.3389/fnana.2015.00076 (PMC4461825; doi:10.3389/fnana.2015.00076)
Supplement: Supplementary file 1 [file Presentation1.PDF]

Frontiers in Neuroanatomy

Research Article
3 February 2015

Supplementary material 1: The expected number of (potential) synapses

Jugoslava Aćimović [1,*], Tuomo Mäki-Marttunen [2,1] and Marja-Leena Linne [1]

[1] *Computational Neuroscience Group, Department of Signal Processing, Tampere University of Technology, Tampere, Finland,*
[2] *Psychosis Research Centre, Institute of Clinical Medicine, University of Oslo, Oslo, Norway.*

Correspondence*:
Jugoslava Aćimović
Computational Neuroscience Group, Department of Signal Processing, Tampere University of Technology, P.O.Box 553, 33101 Tampere, Finland,
jugoslava.acimovic@tut.fi

DERIVATION OF THE EXPECTED NUMBER OF SYNAPSES PER NEURON

In this section, we show the steps to derive the expression for the (potential) synapses $\overline{S}$ (Equation (4) in the paper). Equivalent expressions are already discussed in the literature (Peters et al. (1991), Liley and Wright (1994), van Pelt and van Ooyen (2013)). We present one possible way to derive this expression here in order to better connect it to the analyzed model, and also to better connect it to other derivations presented in this work. This is the first step in the computation of the effective radius, the concept needed to derive the expressions for all of the considered network measures, i.e. motif distributions, clustering coefficient, harmonic path length, and small-world coefficient. Also, we show the solution of the general expression for $\overline{S}$ for the two examples of neurite fields analyzed in this study.

We will first introduce the notation and then explain the adopted description of the neurite trees. Next, we explain the derivation of the expected number of synapses. The approximations used in this derivation are additionally discussed at the end.

Dendrites and axons: The representation of neurite trees is rather approximative, as we take into account only two basic properties, the area filled by the neurite and the density of neurite fibers within that area. We limit the study to planar neurites and 2D geometry, but all of the introduced concepts can be extended to 3D geometries. The density of the neurites is represented by the distribution of the neurite segments within the limited area. In this study we do not take into account the fine structure of the neurites, the organization of neurite segments into trees, the branching statistics, orientation of segments etc. These aspects will be taken into account in our future studies, while here we opted to use the approximate model that can be solved analytically and that captures the mean tendencies of the considered measures of network structure.

A dendrite is represented by its N_d segments. A segment is a small part of a fiber that can be approximated with a cylinder. The length of a segment is very short, e.g. similar to the length of dendritic spines, and is equal for all the segments. Each segment is represented by the (2D) coordinates of its center point. These centers are modeled as an array of independent and identically distributed random values $\{X_i\}_{i=1..N_d}$, each described by a distribution:

$$X_i \sim \begin{cases} p_d(\underline{x}), & \underline{x} \in \Omega_d \\ 0, & else \end{cases}$$

Similarly, an axon is represented by N_a segments with centers $\{Y_i\}_{i=1..N_a}$ where

$$Y_i \sim \begin{cases} p_a(\underline{x}), & \underline{x} \in \Omega_a \\ 0, & else \end{cases}$$

Here, $\underline{x}$ is a (2D) vector of Cartesian coordinates, $\underline{x} = [x\ y]^T$, $p_d(\underline{x})$ and $p_a(\underline{x})$ are the (2D) probability distributions of the coordinates, and Ω_d and Ω_a are the limited areas covered by dendrite and axon.

Synapses: As described in the paper, in this work we adopted the simplest possible rule for synapse formation entirely based on morphology.

1. A dendrite-axon pair forms synapses independently of other axons in the dendrite neighborhood.
2. If we consider one dendrite and axon pair, each dendrite segment forms a contact with maximally one axon segment in its neighborhood. The justification for this assumption will be given in what follows.
3. A neighborhood of a dendrite segment is a ball with a radius D centered in the segment center, D should take the value of an average dendritic spine length. This also determines the dendrite segment length. The segment should fall entirely inside the ball of radius D, so we take the segment length as being $2D$. This way we avoid having the same axon segment in more than one neighborhood, and the possibility that it connects to more than one dendrite segment of the same neuron.

The synapses on a dendrite are represented by an array of discrete random variables $\{S_i\}_{i=1..N_d}$, where S_i is the number of synapses on a dendrite segment with the center in X_i.

The number of synapses per dendrite segment: To compute the expected number of synapses per neuron we first need to estimate the probability that each dendrite segment forms m synapses. At the same time we will give the arguments for the initial assumption, i.e. that the expected number of synapses per segment should be 0 or 1. We will show how the probability of having m segments decreases with the number of segments, and under which conditions it becomes close to zero for $m > 1$.

Consider a dendrite segment X_i with the center in $\underline{x}_k$ and one axon from its neighborhood. We compute the probability that it forms $S_i = m$ synapses with that axon, $P(S_i = m \mid X_i = \underline{x}_k)$. If the axon is represented by N_a segments with indices $\{1, 2, ..., N_a\}$, then we can find $\{l_1, l_2, ..., l_m\} \subset \{1, 2, ..., N_a\}$ such that every segment from that subset falls at a distance smaller than D from X_i, and every segment outside of that set is at a distance larger than D.

$$P(S_i = m|X_i = \underline{x}_k) = P((\forall j \in \{l_1, ...l_m\}) \, \|Y_j - \underline{x}_k\| \leq D \text{ and } (\forall j \notin \{l_{m+1}, ...l_{N_a}\}) \, \|Y_j - \underline{x}_k\| > D)$$

Assuming that the positions of the axon and dendrite segments are independent and distributed inside the areas $\Omega_{a,d}$, the probability becomes:

$$P(S_i = m|X_i = \underline{x}_k) = \binom{N_a}{m} P(\|Y - \underline{x}_k\| \leq D)^m \cdot P(\|Y - \underline{x}_k\| > D)^{N_a - m}$$

As mentioned before, we consider D to be small compared to the other elements of the network (total neurite size, the distance between neurons, etc.). Bearing this in mind, the following three approximations are introduced:

1. The probability that an axon segment Y lies in the small neighborhood around $\underline{x}_k$.
$$P(\|Y - \underline{x}_k\| \leq D) = \begin{cases} \approx p_a(\underline{x}_k) \cdot D^2\pi & \text{if } \underline{x}_k \in \Omega_a \\ 0 & \text{if } \underline{y}_k \notin \Omega_a \end{cases}$$
2. The probability that the N axon segments lie outside of the small neighborhood of $\underline{x}_k$.
$$(1 - p_a(\underline{x}_k)D^2\pi)^N \approx 1 - Np_a(\underline{x}_k)D^2\pi$$
3. In addition, we can assume that $m << N_a$, i.e. a single dendrite segment, will form synapses with a very small percent of available axon segments. This will not hold only for some very peculiar probability distributions, for example, a sum of delta functions, or a Gaussian with very narrow variance compared to the neurite dimensions.

Using these assumptions we obtain the following:

$$\begin{aligned} P(S_i = m | X_i = \underline{x}_k) &\approx \binom{N_a}{m} \left(p_a(\underline{x}_k)D^2\pi\right)^m \cdot (1 - (N_a - m)p_a(\underline{x}_k)D^2\pi) \\ &= \frac{N_a!}{(N_a - m)!m!} \left(p_a(\underline{x}_k)D^2\pi\right)^m \cdot (1 - (N_a - m)p_a(\underline{x}_k)D^2\pi) \end{aligned}$$

From Stirling's approximation and the assumption that $m << N_a$ we have:

$$\begin{aligned} \frac{N_a!}{(N_a - m)!} &\approx \frac{\sqrt{(2\pi N_a)}(N_a/e)^{N_a}}{\sqrt{(2\pi(N_a - m))}((N_a - m)/e)^{N_a - m}} \\ &= \sqrt{\frac{1}{1 - m/N_a}} \left(\frac{N_a}{e}\right)^m \cdot \left(\frac{1}{1 - m/N_a}\right)^{N_a - m} \approx \left(\frac{N_a}{e}\right)^m, \end{aligned}$$

This gives:

$$P(S_i = m | X_i = \underline{x}_k) \approx \frac{1}{m!} \cdot \left(N_a p_a(\underline{x}_k)D^2\frac{\pi}{e}\right)^m \cdot (1 - N_a p_a(\underline{x}_k)D^2\pi).$$

If we select $\frac{\pi}{e} \cdot N_a p_a(\underline{x}_k)D^2 = 1.15 \cdot N_a p_a(\underline{x}_k)D^2 < 1$, the assumption that holds for a sufficiently small D, the values for $P(S_i = m | X_i = \underline{x}_k)$ will rapidly decrease as m increases, which justifies the approximation $P(S_i | X_i = \underline{x}_k) \approx 0$ for $m > 1$. This assumption will be analyzed more carefully for uniform distribution of axons and dendrites and the conditions for model parameters will be given.

We end up with the following probabilities (valid when $\underline{x}_k \in \Omega_a \cap \Omega_d$).

$$P(S_i = m | X_i = \underline{x}_k) = \begin{cases} (1 - p_a(\underline{x}_k)D^2\pi)^{N_a} \approx 1 - N_a p_a(\underline{x}_k)D^2\pi, & m = 0 \\ \approx N_a p_a(\underline{x}_k)D^2\pi & m = 1 \\ \approx 0, & \text{else} \end{cases}$$

The next step is to obtain the probability that a dendrite segment forms m synapses regardless of its position within Ω_d, which is computed by integrating the previous equation over the entire intersection

$\Omega_d \cap \Omega_a$.

$$\begin{aligned} P(S_i = m) &= \int\int_{\Omega_d \cap \Omega_a} P(S_i = m | X_i = \underline{x}) \cdot P(X_i = \underline{x}) d\underline{x} \\ \Rightarrow P(S_i = 0) &= \int\int_{\Omega_d \cap \Omega_a} (1 - N_a p_a(\underline{x}) D^2 \pi) \cdot p_d(\underline{x}) d\underline{x} \\ \Rightarrow P(S_i = 1) &= \int\int_{\Omega_d \cap \Omega_a} (N_a p_a(\underline{x}) D^2 \pi) \cdot p_d(\underline{x}) d\underline{x} \end{aligned}$$

The expected number of synapses on $\Omega_a \cap \Omega_d$ is a summation of the number of synapses on all dendrite segments.

$$\sum_{i=1}^{N_d} P(S_i = m) \cdot m \approx \sum_{i=1}^{N_d} P(S_i = 1) = N_d \int\int_{\Omega_a \cap \Omega_d} N_a p_a(\underline{x}) D^2 \pi p_d(\underline{x}) d\underline{x}$$

Therefore, the expected number of synapses formed between any pair of neurons in the population is:

$$\overline{\mathbf{S}} = \mathbf{N_a N_d D^2} \pi \int\int_{\mathbf{\Omega_a \cap \Omega_d}} \mathbf{p_a(\underline{x}) p_d(\underline{x}) d\underline{x}} \quad (1)$$

Approximations: It is important to look once again at the approximations used in this section. We assumed the following:

1. $p_a(\underline{x}_k) D^2 \pi << 1$
   This assumption was necessary for the approximations: $P(S_i = m > 1 | X_i = \underline{x}_k) \approx 0$, and $(1 - p_a(\underline{x}_k) D^2 \pi)^{N_a} \approx 1 - N_a p_a(\underline{x}_k) D^2 \pi$.
2. $N_a p_a(\underline{x}_k) D^2 \pi < 1$,
   Without this the expressions for $P(S_i = \{0, 1\} | X_i = \underline{x}_k)$ is not a probability, and the expression $\left(N_a p_a(\underline{x}_k) D^2 \frac{\pi}{e}\right)^m$ might not become close to zero as m increases.

The first assumption follows from the second one and the choice of dendrite segments. They should be very small compared to the total length of dendrites, which implies large values of N_a.

To justify the second assumption we need to closely examine $p_a(\cdot)$. For the uniform probability distribution $p_a(\underline{x}) = \frac{1}{R_a^2 \pi}$ for $\underline{x} \in \Omega_a$, which gives $N_a p_a(\underline{x}) D^2 \pi = N_a \left(\frac{D}{R_a}\right)^2$. If we assume $N_a \sim 10^3$, $D \sim 1\mu m$, $R_a \sim 100\mu m$, the values close to the estimations for stellate cells, we get $N_a p_a(\underline{x}) D^2 \pi \sim 0.1$. For bigger values of R_a, the neurite radius, these values become smaller.

Now consider the truncated Gaussian distribution as the one proposed in (Snider et al. (2010)). The probability distribution is $p_a(\underline{x}) = \frac{1}{2\pi\sqrt{|\Sigma| \cdot C}} \cdot \exp\left(-\frac{1}{2}(\underline{x} - \underline{x}_A)^T \Sigma^{-1} (\underline{x} - \underline{x}_A)\right)$, where Σ is the covariance matrix of the distribution, $\underline{x}_A$ are the coordinates of the center of axon distribution, T is the vector transposition (we assume that $\underline{x}$ is a 2x1 vector and $\underline{x}^T$ is a 1x2 vector), and C the normalization factor. This factor is equal to $C = \frac{1}{2\pi\sqrt{|\Sigma|}} \int\int_{\Omega_a} \exp\left(-\frac{1}{2}(\underline{x} - \underline{x}_A)^T \Sigma^{-1} (\underline{x} - \underline{x}_A)\right) d\underline{x}$. The probability $p_a(\underline{x})$ reaches its maximum when $\underline{x} = \underline{x}_A$, and equals $p_A(\underline{x}_A) = \frac{1}{2\pi\sqrt{|\Sigma|}\, C}$. For simplicity, suppose that the distribution

has a circular support of radius R_a, a variance σ_a and the center $\underline{x}_A$ in the coordinate center. The upper bound for the probability distribution becomes:

$$p_a(\underline{x}) \leq p_a(\underline{x}_A) = \left(\int_{-\pi}^{\pi} \int_{0}^{R_a} \exp\left(-\frac{r^2}{2\sigma_a^2} \right) \, r \, dr \, d\alpha \right)^{-1}$$

$$\Rightarrow \quad N_a p_a(\underline{x}_A) D^2 \pi = N_a \left(\frac{D}{R_a} \right)^2 \frac{\frac{R_a^2}{2\sigma_a^2}}{1 - \exp(-\frac{R_a^2}{2\sigma_a^2})} = N_a \left(\frac{D}{R_a} \right)^2 \cdot f\left(\frac{R_a}{\sqrt{2}\sigma_a} \right)$$

Assuming the same values for $N_a,\ R_a,\ D$ as before, the last expression becomes bigger than 1 for $\sigma_a < 0.224\ R_a$. The initial assumptions fail when most of the neurite segments are concentrated around the neurite center, and very few of them get further from it. Bearing in mind the branching and elongation model for neurite trees (for example, the one used in Koene et al. (2009)) this is an unlikely scenario.

DERIVATION OF THE EXPECTED NUMBER OF SYNAPSES FOR UNIFORMLY DISTRIBUTED NEURITES

If we assume uniform distribution of the neurite segments within the circle with the radius $R_{a/d}$, we can further develop the expression for the expected number of synapses between a dendrite-axon pair.

$$\overline{S} = N_a N_d D^2 \pi \int\int_{\Omega_a \cap \Omega_d} p_a(x_1, x_2)\, p_d(x_1, x_2)\, dx_1\, dx_2 = \frac{N_a N_d D^2}{R_a^2 R_d^2 \pi} \, \|\Omega_a \cap \Omega_d\|$$

The expected number of synapses depends on the intersection area between the axon and the dendrite (Peter's rule, Peters et al. (1976), Peters et al. (1991)). Figure 1 shows three possible situations for the two intersecting circles, the red one represents an axon, and the blue one a dendrite. We will assume that $R_a \geq R_d$, which is usually true, but a similar analysis can be done for the opposite relation between the neurite radii. Figure 1: panels from left to right correspond to the situations:

- $R_a \leq \Delta \leq R_a + R_d$ (the left most panel on the figure)
- $R_a - R_d \leq \Delta \leq R_a$ (the middle panel)
- $\Delta \leq R_a - R_d$ (the right most panel)

Here, Δ is the distance between the neurite centers and $\Delta_1,\ \Delta_2$ are the distances from the axon and dendrite centers to the line connecting the two intersection points. In the first case, when $R_a \leq d \leq R_a + R_d$, the intersection area of neurites can be expressed as:

$$\|\Omega_a \cap \Omega_d\| = R_a^2 \cdot \arccos\left(\frac{\Delta_1}{R_a} \right) - \Delta_1 R_a \sqrt{1 - \frac{\Delta_1^2}{R_a^2}} + R_d^2 \cdot \arccos\left(\frac{\Delta_2}{R_d} \right) - \Delta_2 R_d \sqrt{1 - \frac{\Delta_2^2}{R_d^2}}$$

We introduce the substitutes: $\mathbf{2R} = \mathbf{R_a} + \mathbf{R_d}$ (the mean radius of the neurites), $\mathbf{2R}' = \mathbf{R_a} - \mathbf{R_d}$, and $\boldsymbol{\Delta} = \boldsymbol{\Delta_1} + \boldsymbol{\Delta_2}$ (SA = soma-to-axon distance, or the dendrite-axon distance if we assume that the dendrite center is in the soma). From the figure it is evident that $R_a^2 - \Delta_1^2 = R_d^2 - \Delta_2^2$, which gives $\Delta_1 =$

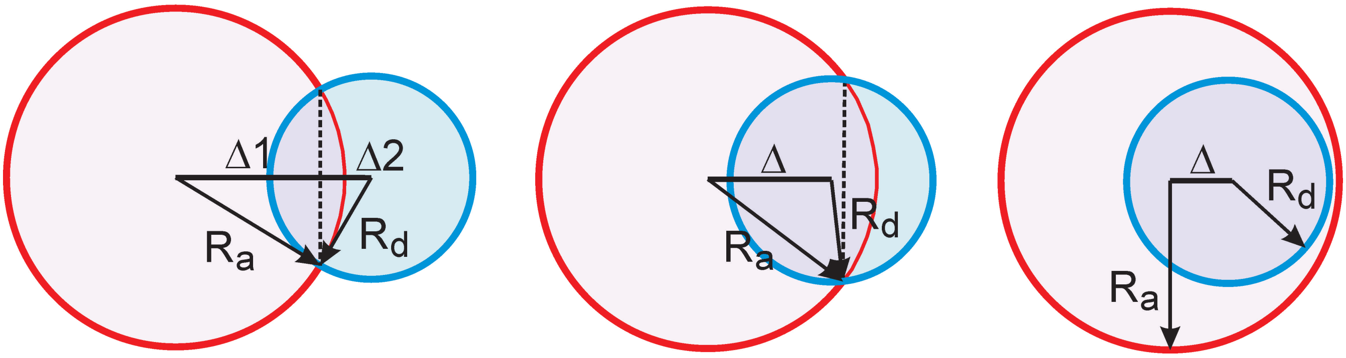

Figure 1. The intersection of two unequal circles; red circle - axon of the radius R_a, blue circle - dendrite of the radius R_d. The dashed line connects the two intersection points. The distance from the dashed line to the axon center is Δ_1, and to the dendrite center is Δ_2. The distance between the axon and dendrite centers is $\Delta = \Delta_1 + \Delta_2$.

$\frac{d^2+R_a^2-R_d^2}{2\Delta_{ad}} = \frac{d^2+4RR^{'}}{2\Delta_{ad}}$ and also $\Delta_2 = \frac{d^2-R_a^2+R_d^2}{2\Delta_{ad}} = \frac{d^2-4RR^{'}}{2\Delta_{ad}}$. Furthermore:

$$
\begin{aligned}
\|\Omega_a \cap \Omega_d\| &= (R+R^{'})^2 \cdot \arccos\left(\frac{\Delta_{ad}^2+4RR^{'}}{2\Delta_{ad}\cdot(R+R')}\right) \\
&- \frac{\Delta_{ad}^2+4RR^{'}}{2\Delta_{ad}} \cdot (R+R^{'}) \cdot \sqrt{1-\frac{(\Delta_{ad}^2+4RR')^2}{4\Delta_{ad}^2(R+R')^2}} \\
&+ (R-R^{'})^2 \cdot \arccos\left(\frac{\Delta_{ad}^2-4RR^{'}}{2\Delta_{ad}\cdot(R-R')}\right) \\
&- \frac{\Delta_{ad}^2-4RR^{'}}{2d} \cdot (R-R^{'})\sqrt{1-\frac{(\Delta_{ad}^2-4RR')^2}{4\Delta_{ad}^2(R-R')^2}} \\
&= (R+R^{'})^2 \cdot \arccos\left(\frac{\Delta_{ad}^2+4RR^{'}}{2\Delta_{ad}\cdot(R+R')}\right) + (R-R^{'})^2 \cdot \arccos\left(\frac{\Delta_{ad}^2-4RR^{'}}{2\Delta_{ad}\cdot(R-R')}\right) \\
&- \frac{1}{2}\sqrt{(4R^2-\Delta_{ad}^2)(\Delta_{ad}^2-4(R')^2)}
\end{aligned}
$$

Here, new substitutions can be introduced: $\eta = \frac{\mathbf{R}^{'}}{\mathbf{R}} = \frac{\mathbf{R_a-R_d}}{\mathbf{R_a+R_d}}$ and $\rho = \frac{\mathbf{\Delta_{ad}}}{\mathbf{2R}} = \frac{\mathbf{\Delta_{ad}}}{\mathbf{R_a+R_d}}$. The expression for the surface of the intersection area becomes:

$$
\begin{aligned}
\|\Omega_a \cap \Omega_d\| &= R^2(1+\eta)^2 \arccos\left(\frac{\eta+\rho^2}{\rho(1+\eta)}\right) + R^2(1-\eta)^2 \arccos\left(\frac{\rho^2-\eta}{\rho(1-\eta)}\right) \\
&- 2R^2\sqrt{(1-\rho^2)(\rho^2-\eta^2)} \\
&= R^2(1+\eta)^2(1-\eta)^2 \cdot \phi(\rho,\eta), \\
\text{where } \phi(\rho,\eta) &= \frac{\arccos\left(\frac{\rho^2+\eta}{\rho(1+\eta)}\right)}{(1-\eta)^2} + \frac{\arccos\left(\frac{\rho^2-\eta}{\rho(1-\eta)}\right)}{(1+\eta)^2} - 2\frac{\sqrt{(1-\rho^2)(\rho^2-\eta^2)}}{(1-\eta^2)^2}.
\end{aligned}
$$

Finally, by replacing the expression above with the one for the expected number of synapses we get:

$$\overline{S} = \frac{N_a N_d D^2}{\pi} \cdot \left(\frac{R_a + R_d}{2R_a R_d}\right)^2 (1-\eta^2)^2 \phi(\rho,\eta) = \frac{N_a N_d D^2}{R^2\pi}\phi(\rho,\eta)$$

If the model parameters are such that $R_a - R_d \leq \Delta \leq R_a \Rightarrow \eta \leq \rho \leq \frac{1+\eta}{2}$.

$$\|\Omega_a \cap \Omega_d\| = R_a^2 \cdot \arccos\left(\frac{\Delta_1}{R_a}\right) - \Delta_1 R_a \sqrt{1 - \frac{\Delta_1^2}{R_a^2}} + R_d^2\pi - R_d^2 \cdot \arccos\left(\frac{\Delta_2}{R_d}\right) + \Delta_2 R_d \sqrt{1 - \frac{\Delta_2^2}{R_d^2}}$$

In this case $\Delta_{ad} = \Delta_1 - \Delta_2$ and, as before, $R_a^2 - \Delta_1^2 = R_d^2 - \Delta_2^2$, which gives $\Delta_1 = \frac{\Delta_{ad}^2 + 4RR'}{2\Delta_{ad}} = R \cdot \frac{\rho^2+\eta}{\rho}$ and $\Delta_2 = \frac{-\Delta_{ad}^2 + 4RR'}{2\Delta_{ad}} = R \cdot \frac{-\rho^2+\eta}{\rho}$. When we replace these and $R_a = R + R' = R(1+\eta)$ and $R_d = R(1-\eta)$ into the previous equation:

$$\begin{aligned}
\|\Omega_a \cap \Omega_d\| &= R^2(1+\eta)^2 \cdot \arccos\left(\frac{\rho^2+\eta}{\rho(1+\eta)}\right) - R^2\frac{\rho^2+\eta}{\rho}(1+\eta)\sqrt{1 - \frac{(\rho^2+\eta)^2}{\rho^2(1+\eta)^2}} \\
&+ R^2(1-\eta)^2\pi - R^2(1-\eta)^2 \cdot \arccos\left(\frac{-\rho^2+\eta}{\rho(1-\eta)}\right) \\
&+ R^2\frac{-\rho^2+\eta}{\rho}(1-\eta)\sqrt{1 - \frac{(-\rho^2+\eta)^2}{\rho^2(1-\eta)^2}} \\
&= R^2(1+\eta)^2 \cdot \arccos\left(\frac{\rho^2+\eta}{\rho(1+\eta)}\right) + R^2(1-\eta)^2 \arccos\left(\frac{\rho^2-\eta}{\rho(1-\eta)}\right) \\
&- 2R^2\sqrt{(1-\rho^2)(\rho^2-\eta^2)}
\end{aligned}$$

Obviously, the expression for $\|\Omega_a \cap \Omega_d\|$ is the same as in the previous case.

The last possible situation is $\Delta_{ad} \leq R_a - R_d$, i.e. $\rho \leq \eta$, when we have $\|\Omega_a \cap \Omega_d\| = R_d^2\pi = R^2(1-\eta)^2\pi$. The expression for the expected number of synapses becomes:

$$\overline{S} = \frac{N_a N_d D^2}{R^4(1-\eta^2)^2\pi} R^2(1-\eta)^2\pi = \frac{N_a N_d D^2}{R^2\pi} \cdot \frac{\pi}{(1+\eta)^2}$$

All these equations can be combined into the following expression:

$$\overline{S} = \frac{N_a N_d D^2}{R^2\pi} \cdot \phi(\rho,\eta) = \frac{4N_a N_d D^2}{\Delta^2\pi}\rho^2\phi(\rho,\eta) \tag{2}$$

The function $\phi(\rho,\eta)$ is extended to take into account the last case:

$$\phi(\rho,\eta) = \begin{cases} \frac{\pi}{(1+\eta)^2}, & 0 \leq \rho \leq \eta \\ \frac{\arccos\left(\frac{\rho^2+\eta}{\rho(1+\eta)}\right)}{(1-\eta)^2} + \frac{\arccos\left(\frac{\rho^2-\eta}{\rho(1-\eta)}\right)}{(1+\eta)^2} - \frac{2\sqrt{(1-\rho^2)(\rho^2-\eta^2)}}{(1-\eta^2)^2}, & \eta < \rho \leq 1 \end{cases} \tag{3}$$

Note: For $\eta = 1$ the value $(1-\eta)^2 = 0$, but because $\rho \leq 1$ the function must be equal to $\phi(\rho, 1) = \frac{\pi}{4}$.

DERIVATION OF THE EXPECTED NUMBER OF SYNAPSES FOR TRUNCATED GAUSSIAN DISTRIBUTION

For the truncated Gaussian distribution it is difficult to compute the analytic solution, as in the previous case. Instead, only the general expression will be given here, and in the Result section this expression will be evaluated numerically. As already described, assume that the coordinate center is in the axon center, and the x ax is along the line connecting the centers of the intersecting axon and dendrite. The Cartesian coordinates are expressed as $\underline{x} = [x \;\; y]^T$, a 2x1 dimension vector. A normalized version of polar coordinates will be used. We will analyze only a less general version of the truncated Gaussian distribution, but sufficient to demonstrate the steps in the computation of mean degree and effective radius. The following is assumed:

- Circular support for the axon and dendrite distributions with the radii R_a and R_d, respectively.
- Variances of the axon distribution in 2D: $\sigma_{ax} = \sigma_{ay} = \sigma_a$, and variances of the dendrite distribution in 2D: $\sigma_{dx} = \sigma_{dy} = \sigma_d$
- No cross-correlation $\sigma_{axy} = \sigma_{dxy} = 0$.

More general forms of the truncated Gaussians can also be considered following the same procedure.

The probability distributions become:

$$p_a(\underline{x}) = \begin{cases} \frac{1}{2\pi\sigma_a^2\, C_a} \exp\left(-\frac{x^2+y^2}{2\sigma_a^2}\right), & \underline{x} \in \Omega_a \\ 0, & \text{else.} \end{cases}$$

$$p_d(\underline{x}) = \begin{cases} \frac{1}{2\pi\sigma_d^2\, C_d} \exp\left(-\frac{(x-\Delta)^2+y^2}{2\sigma_d^2}\right), & \underline{x} \in \Omega_d \\ 0, & \text{else.} \end{cases}$$

Here Δ is the distance between the axon and dendrite centers, and at the same time the coordinates of the dendrite center in the described coordinate system fixed to the axon center. The coefficients C_a and C_d compensate for the truncation of the Gaussians, i.e. they ensure that $p_a(\underline{x})$ and $p_d(\underline{x})$ are probability distributions. The coefficient C_a is equal to:

$$C_a = \frac{1}{2\pi\sigma_a^2} \int\int_{x^2+y^2 \le R_a} \exp\left(-\frac{x^2+y^2}{2\sigma_a^2}\right) dx\, dy = 1 - \exp\left(-\frac{R_a^2}{2\sigma_a^2}\right) = 1 - \exp\left(-\frac{k_\sigma^2(1+\eta)^2}{2\sigma^2}\right)$$

Similarly, the other coefficient is:

$$C_d = 1 - \exp\left(-\frac{R_d^2}{2\sigma_d^2}\right) = 1 - \exp\left(-\frac{(1-\eta)^2}{2\sigma^2}\right)$$

The expression for the expected number of synapses becomes:

$$\overline{S} \;=\; \frac{N_a N_d D^2 \pi}{4\pi^2\sigma_a^2\sigma_d^2\, C_a\, C_d} \int\int_{\Omega_a \cap \Omega_d} \exp\left(-\frac{x^2+y^2}{2\sigma_a^2} - \frac{(x-\Delta)^2+y^2}{2\sigma_d^2}\right) dx\, dy$$

Next we introduce the following normalizations to simplify the equations $\Delta = 2R\rho$, $\sigma_d = 2R\sigma$, $\sigma_a = \frac{2R\sigma}{k_\sigma}$. In addition, the coordinates are transformed from Cartesian to polar coordinates with a normalized

radius: $x = 2R \cdot r\cos(\alpha)$, $y = 2R \cdot r\sin(\alpha)$. This gives the following expression for the expected number of synapses:

$$\begin{aligned} \overline{S} &= \frac{N_a\, N_d\, D^2}{R^2\pi} \cdot \frac{k_\sigma^2\ \exp\left(-\frac{\rho^2}{2\sigma^2}\right)}{16\ \sigma^4\ \left(1 - \exp\left(-\frac{k_\sigma^2(1+\eta)^2}{2\sigma^2}\right)\right)\ \left(1 - \exp\left(-\frac{(1-\eta)^2}{2\sigma^2}\right)\right)} \times \\ &\times \int\int_{\Omega_a \cap \Omega_d} \exp\left(-\frac{r^2}{2\sigma^2}(k_\sigma^2 + 1) + r\frac{\rho}{\sigma^2}\cos(\alpha)\right)\ r\ dr\ d\alpha \end{aligned}$$

The domain of integration $\Omega_a \cap \Omega_d$ depends on the mutual position of the axon and dendrite. Assume, as before, that $R_d \leq R_a$. The expression for the circular border of the dendrite is $(x - \Delta)^2 + y^2 = R_d^2$, and expressed in the normalized polar coordinates it becomes:

$$r^2 - 2r\rho\cos(\alpha) + \rho^2 = \left(\frac{1-\eta}{2}\right)^2$$

The solutions for this equation are:

$$r^{\pm}(\alpha) = \rho\cos(\alpha) \pm \sqrt{\left(\frac{1-\eta}{2}\right)^2 - \rho^2\sin^2(\alpha)}$$

The tangent point from the axon center to the circle is given by the coordinates:

$$\alpha_t = \arcsin\left(\frac{1-\eta}{2\rho}\right), \quad r_t = \rho\sqrt{1 - \sin^2(\alpha_t)} = \frac{1}{2}\sqrt{4\rho^2 - (1-\eta)^2}$$

The intersection points between the axon and dendrite borders are obtained for $r = \frac{R_a}{2R} = \frac{1+\eta}{2}$ and are given by the angles:

$$\frac{(1+\eta)^2}{4} - \rho(1+\eta)\cos(\alpha) + \rho^2 = \frac{(1-\eta)^2}{4} \Rightarrow \alpha_i = \pm\arccos\left(\frac{\eta + \rho^2}{(1+\eta)\rho}\right)$$

The following cases have to be considered: whether the tangent points are inside or outside of the axon, whether the entire dendrite is inside the axon, whether the axon center is inside the dendrite. The five possible situations are summarized in Table **??**. Finally, the expected number of synapses can be expressed in the same format as the one derived for the uniform distribution of neurite segments:

$$\begin{aligned} \overline{S} &= \frac{N_a N_d D^2}{R^2\pi} \cdot \phi(\rho, \eta, M) \\ \phi(\rho, \eta, M) &= \frac{k_\sigma^2 \exp\left(-\frac{\rho^2}{2\sigma^2}\right)}{16\sigma^4\left(1 - \exp\left(-\frac{k_\sigma^2(1+\eta)^2}{2\sigma^2}\right)\right)\left(1 - \exp\left(-\frac{(1-\eta)^2}{2\sigma^2}\right)\right)} \times \\ &\times \int\int_{\mathcal{D}(\rho,\eta,M)} \exp\left(-\frac{r^2}{2\sigma^2}(k_\sigma^2 + 1) + r\frac{\rho}{\sigma^2}\cos(\alpha)\right)\ r\ dr\ d\alpha \end{aligned}$$

Here, $\mathcal{D}(\rho, \eta, M)$ is the integration domain that is given by the table below, and M is the set of parameters that depends on the neurite field distribution, in this case $M = [\sigma\ k_\sigma]$. The function $\phi(\cdot)$ can be evaluated using numerical integration, and is further analyzed in Results section. More general forms of the truncated Gaussian distribution (with elliptic support, with a different covariance matrix), as well as other possible distributions of neurite segments, can be analyzed using the same procedure.

Table 1.A summary of all possible shapes of the integration domain $\Omega_a \cap \Omega_d$ that should be taken into account when analyzing the function $\phi(\cdot)$ for neurites with truncated Gaussian distribution. In the table: $r^{\pm}(\alpha) = \rho\cos(\alpha) \pm \sqrt{\left(\frac{1-\eta}{2}\right)^2 - \rho^2\sin^2(\alpha)}$, $\alpha_i = \arccos\left(\frac{\eta+\rho^2}{(1+\eta)\rho}\right)$, $\alpha_t = \arcsin\left(\frac{1-\eta}{2\rho}\right)$. The integration domain depends on the normalized parameters ρ, η, and σ.

Case	Conditions	$\rho,\ \eta$	$r^{\pm}$	α
Tangent points outside the axon	$r_t \geq R_a$	$\sqrt{\frac{1+\eta^2}{2}} \leq \rho < 1$	$\left[r^-(\alpha), \frac{1+\eta}{2}\right]$	$[-\alpha_i,\ \alpha_i]$
Tangent points inside the axon,	$r_t < R_a$	$\max\{\eta, \frac{1-\eta}{2}\} < \rho$	$\left[r^-(\alpha), \frac{1+\eta}{2}\right]$	$[-\alpha_i,\ \alpha_i]$
part of the dendrite outside the axon,<br>the axon center outside the dendrite	$\Delta + R_d > R_a$<br>$\Delta > R_d$	$\rho < \sqrt{\frac{1+\eta^2}{2}}$	$\left[r^-(\alpha), r^+(\alpha)\right]$	$[-\alpha_t, -\alpha_i]$<br>$\cup[\alpha_i, \alpha_t]$
Tangent points inside the axon,<br>the whole dendrite inside the axon,<br>the axon center outside the dendrite	$r_t < R_a$<br>$\Delta + R_d \leq R_a$	$\frac{1-\eta}{2} < \rho \leq \eta$<br>$\eta > \frac{1}{3}$	$\left[r^-(\alpha), r^+(\alpha)\right]$	$[-\alpha_t, \alpha_t]$
Part of the dendrite outside the axon,	$\Delta + R_d > R_a$	$\eta < \rho \leq \frac{1-\eta}{2}$	$\left[0, \frac{1+\eta}{2}\right]$	$[-\alpha_i, \alpha_i]$
the axon center inside the dendrite	$\Delta < R_d$	$\eta \leq \frac{1}{3}$	$[0, r^+(\alpha)]$	$[-\pi, -\alpha_i]$<br>$\cup[\alpha_i, \pi]$
The whole dendrite inside the axon,<br>the axon center inside the dendrite	$\Delta + R_d \leq R_a$<br>$\Delta \leq R_d$	$\rho \leq \min\{\eta, \frac{1-\eta}{2}\}$	$[0, r^+(\alpha)]$	$[-\pi, \pi]$
