# Supplementary material for: The effects of neuron morphology on graph theoretic measures of network connectivity: the analysis of a two-level statistical model
Source: Front Neuroanat. 2015 Jun 10;9:76. doi: 10.3389/fnana.2015.00076 (PMC4461825; doi:10.3389/fnana.2015.00076)
Supplement: Supplementary file 2 [file Presentation2.PDF]

Supplementary material 2: Computation of the expected number of motif, implementation details

Jugoslava Aćimović [1,*], Tuomo Mäki-Marttunen [2,1] and Marja-Leena Linne [1]

[1] *Computational Neuroscience Group, Department of Signal Processing, Tampere University of Technology, Tampere, Finland,*
[2] *Psychosis Research Centre, Institute of Clinical Medicine, University of Oslo, Oslo, Norway.*

Correspondence*:
Jugoslava Aćimović
Computational Neuroscience Group, Department of Signal Processing, Tampere University of Technology, P.O.Box 553, 33101 Tampere, Finland,
jugoslava.acimovic@tut.fi

In what follows we describe the additional steps needed to compute the expected motif counts from the equations presented in the paper. These equations contain multiple integrals that were evaluated using the Matlab built-in function quad2d, for integration of double integrals, and a standard trapezoid method for the inner most integral in expressions for motifs M2 and M9. The function quad2d is sensitive to discontinuities in integrated functions. In the first section we describe the steps to avoid such discontinuities. The second section shows computation of the correction coefficients for 3-node motifs. These corrections removed cases when two or three dendrite centers overlap.

STEPS TO AVOID DISCONTINUITIES WHEN EVALUATING MULTIPLE INTEGRALS IN THE EXPECTED MOTIF COUNT EQUATIONS

As described in the paper, the counts for the tree-node motifs can be expressed as quadruple integrals for most of the motifs, or quintuple for motifs M2 and M9. These integrals cannot be evaluated straightforwardly for the entire domain $\mathcal{B}_{r_{max}}(A1)$ due to the discontinuities of the integrated function. In this section we show several steps in the implementation of motif counts, used to avoid singularities. The following issues caused singularities:

- The functions τ and τ^{-1} describe the change of polar coordinates when the coordinate center is translated. This change of coordinates is described with the arc-tangent function which is defined on the interval $\left[\frac{\pi}{2}, \frac{\pi}{2}\right]$. This might cause problems as the angles are defined on the interval $[-\pi, \pi]$, but this problem is easily avoided by using Matlab's function **atan2** that implements the arc-tangent in all four quadrants.
- The function $\lambda^{'}$ measures the length of the intersection between the circle that defines the potential axon positions, $\mathcal{C}_1$, and the connectivity areas of the two other dendrites. This length depends on the accurate evaluation of the intersection angles. The angles are computed using the functions τ, κ, κ_1, and τ^{-1} and the obtained results fall on the interval $[-\pi, \pi]$. This is another source of discontinuities. If some of the steps in the results computation give an intersection angle bigger than π or smaller than $-\pi$, the angle is automatically shifted to the interval $[-\pi, \pi]$, which wrongly evaluates some of

conditions used to compute $\lambda^{'}$ and causes a discontinuity in λ. This problem is solved by checking the sign of the intersection angles and, if needed, shifting them for $\pm 2\pi$ before computing $\lambda^{'}$.

- The biggest problem with discontinuities arises from the sole definition of functions κ, κ_1 and λ, i.e. the functions are defined differently for different values of the distances between three dendrite centers. This problem is solved by dividing the entire integration domain $\mathcal{B}_{r_{max}}(A1)$ into sub-domains and evaluating the expected number of motifs on these intervals. The borders of the sub-domains correspond to the discontinuities. Therefore, within each sub-domain, each function is continuous and can be integrated. In the rest of this section we explain the derivation of the sub-domains of integration.
- Additionally, some positions of the dendrite centers cause the function $\lambda^{'}$ to drop to zero. These situations are also considered when defining the sub-domains for integration.

From the definitions of κ, κ_1, λ, and $\lambda^{'}$, the following relations between dendrite centers lead to discontinuities:

- $\|B1B2\| = r_{max} - 1$, $\|B1B3\| = r_{max} - 1$
- $\|B2B3\| = r_{max} - 1$, $\|B2B3\| = r_{max} + 1$
- $\|B1B2\| = 2r_{max}$, $\|B1B3\| = 2r_{max}$ (cases G and H from Figure 2).

Each of these conditions represents a circular boundary that imposes a rapid change in some of the functions. In order to obtain the areas where all functions are monotonic, we have to find the intersections between all of these circles and their intersections with the integration domain $\mathcal{B}_{r_{max}}(A1)$. The derivation of the exact equations is difficult as it requires considering many conditions. Instead, we used the following algorithm implemented in Matlab. First, we compute all the intersection angles between circles and all the tangent angles from A1 to those circles (if they exist). These expressions are given below. Each interval between two successive angles is considered separately. For each interval we selected a representative point (usually the middle of the interval), then we evaluated the expressions for all the circles for that point. The relations between the obtained values determine how the boundaries are arranged within the considered interval. Knowing this, we can further divide the interval into sub-domains between the boundaries and evaluate integrals for each sub-domain. The intersection angles, the representative angle, boundaries and sub-domains are illustrated in Figure 1.

Figure 1 explains the selection of sub-domains for the dendrite center B3 when parameter r_{max} and point $B2(\alpha_2, r_2)$ are fixed. This situation corresponds to $1 < r_{max} < 2$. Discontinuities in the integrated functions appear when B3 belongs to one of the circles $\mathcal{C}_{|r_{max}-1|}(B1)$, $\mathcal{C}_{2r_{max}}(B1)$, $\mathcal{C}_{|r_{max}-1|}(B2)$, or $\mathcal{C}_{r_{max}+1}(B2)$. For the presented case, i.e. when $r_{max} > 1$ the circle $\mathcal{C}_{2r_{max}}(B1)$ does not intersect with the integration domain $\mathcal{B}_{r_{max}}(A1)$. Similarly, when $r_{max} < 1$, the circle $\mathcal{C}_{|r_{max}-1|}(B1)$ is entirely outside of the integration domain. In the presented case the integration and tangent angles that should be considered are the following:

- Intersection angles $\mathcal{C}_{r_{max}}(A1) \cap \mathcal{C}_{|r_{max}-1|}(B2)$: α_{i1}, α_{i2}
- Intersection angles $\mathcal{C}_{r_{max}}(A1) \cap \mathcal{C}_{|r_{max}-1|}(B1)$: α_{i3}, α_{i4}
- Intersection angles $\mathcal{C}_{r_{max}}(A1) \cap \mathcal{C}_{r_{max}+1}(B2)$: α_{i5}, α_{i6}
- Tangent angles $\mathcal{C}_{|r_{max}-1|}(B2)$: α_{t1}, α_{t2}
- Tangent angles $\mathcal{C}_{|r_{max}-1|}(B1)$: α_{t3}, α_{t4}

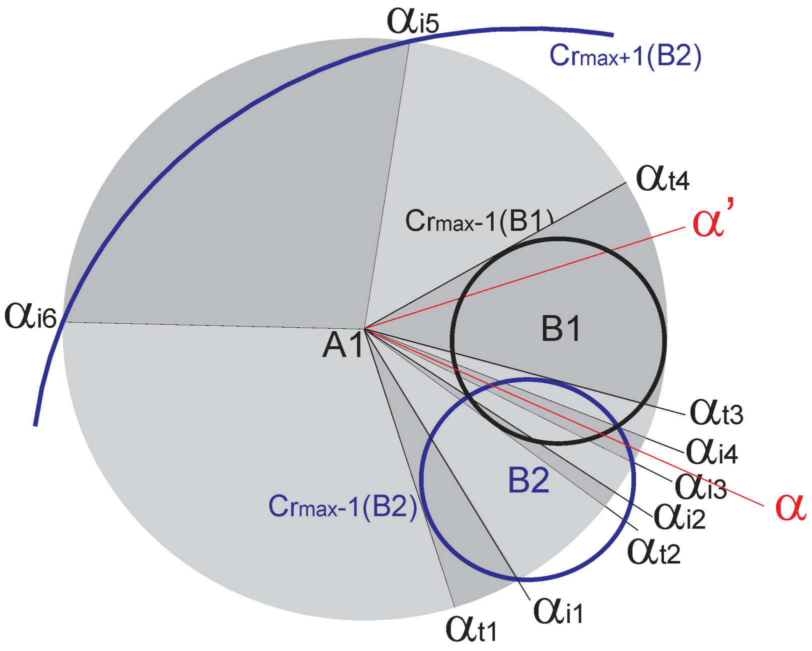

Figure 1. Division of the integration domain ($\mathcal{B}_{r_{max}}(A1)$) to avoid discontinuities in functions κ, κ_1, λ, example for $r_{max} > 1$. The domains for the dendrite center B3 are determined by the circles $\mathcal{C}_{r_{max}-1}(B1)$, $\mathcal{C}_{r_{max}-1}(B2)$ and $\mathcal{C}_{r_{max}+1}(B2)$. The intersection points between the circles are marked as α_i, tangent points from A1 to the circles are marked as α_t. The intervals between successive angles (intersection or tangent angles) are emphasized by alternating shades of gray. Each of these intervals is considered separately and divided into smaller regions between the circle borders.

Considering the relations between these listed angles the list of intervals that should be considered is $[-\pi, \alpha_{t1}]$, $[\alpha_{t1}, \alpha_{i1}]$, $[\alpha_{i1}, \alpha_{t2}]$, $[\alpha_{t2}, \alpha_{i2}]$, $[\alpha_{i2}, \alpha_{i3}]$, $[\alpha_{i3}, \alpha_{i4}]$, $[\alpha_{i4}, \alpha_{t3}]$, $[\alpha_{t3}, 0]$, $[0, \alpha_{t4}]$, $[\alpha_{t4}, \alpha_{i5}]$, $[\alpha_{i5}, \alpha_{i6}]$, and $[\alpha_{i6}, \pi]$ (in case $\alpha_{i6} > \pi$ the first intervals are $[\pi, \alpha_{i6}]$, $[\alpha_{i6}, \alpha_{t1}]$ and the last is $[\alpha_{i5}, \pi]$). Furthermore, each of these intervals should be subdivided so that the final sub-domains contain no singularities. Consider the two angles highlighted on Figure 1, α and $\alpha^{'}$ (in red). For each angle the circles define the range of values for the radial coordinate. For α the first domain is between $r = 0$ and the boundary of $\mathcal{C}_{|r_{max}-1|}(B2)$, the second interval is inside the circle $\mathcal{C}_{|r_{max}-1|}(B2)$, and the third interval is between this circle and $r = r_{max}$. For $\alpha^{'}$ the situation is similar, except that the second interval falls inside the circle $\mathcal{C}_{|r_{max}-1|}(B1)$, and so on. In the example shown on Figure 1, the entire domain of integration is divided into 33 sub-domains. Some of these sub-domains might be very small and not suitable for the estimation of integrals. This is taken care of in the Matlab function for numerical integration **quad2d** used to evaluate the expected number of motifs. Such small intervals may fall below the required minimal rectangle size in the approximate method for integral computation. Still, in the considered cases, this did not affect the precision of the numerical computations.

In addition, the domain of the integration for B2 should also be divided according to the circles $\|B1B2\| = |r_{max} - 1|$ and $\|B1B2\| = 2r_{max}$. For $r_{max} < 1$ the first circle is outside of $\mathcal{B}_{r_{max}}(A1)$ and does not need to be considered. The second circle divides the integration domain for B2 into two areas and the shape of those areas changes for $r_{max} = \frac{1}{3}$ (intersects with $\mathcal{B}_{r_{max}}(A1)$ for $r_{max} > \frac{1}{3}$), $r_{max} = \frac{1}{\sqrt{5}}$ (the tangent points of the circles enter the area $\mathcal{B}_{r_{max}}(A1)$), $r_{max} = \frac{1}{2}$ (A1 enters the circle $\mathcal{B}_{1-r_{max}}(B1)$, the tangent points do not exist anymore), $r_{max} = 1$ (B1 enters the area $\mathcal{B}_{r_{max}}(A1)$). For $r_{max} > 1$ the area $\mathcal{B}_{r_{max}}(A1)$ is entirely inside the circle $\mathcal{B}_{2r_{max}}(B1)$ and the two have no intersections. For $r_{max} > 2$ point A1 enters the circle $\mathcal{C}_{r_{max}-1}(B1)$.

The equations for the listed circles and their tangent angles:

$$\mathcal{C}_{|r_{max}-1|}(B1): \quad r^{\pm}(\alpha) = \cos(\alpha) \pm \sqrt{(r_{max}-1)^2 - \sin(\alpha)^2}, \quad \alpha_t = \pm\arcsin\left(|r_{max}-1|\right)$$

$$\mathcal{C}_{2r_{max}}(B1): \quad r^{\pm}(\alpha) = \cos(\alpha) \pm \sqrt{4r_{max}^2 - \sin(\alpha)^2}, \quad \alpha_t = \pm\arcsin\left(2r_{max}\right)$$

$$\mathcal{C}_{|r_{max}-1|}(B2): \quad r^{\pm}(\alpha) = r_2\cos(\alpha-\alpha_2) \pm \sqrt{(r_{max}-1)^2 - r_2^2\sin(\alpha-\alpha_2)^2},$$
$$\alpha_t = \alpha_2 \pm \arcsin\left(\frac{|r_{max}-1|}{r_2}\right)$$

$$\mathcal{C}_{r_{max}+1}(B2): \quad r^{\pm}(\alpha) = r_2\cos(\alpha-\alpha_2) \pm \sqrt{(r_{max}+1)^2 - r_2^2\sin(\alpha-\alpha_2)^2}$$
$$\text{(the tangent points are outside of } \mathcal{B}_{r_{max}}(A1))$$

The intersection angles between the considered circles (when they exist):

$$\mathcal{C}_{r_{max}}(A1) \cap \mathcal{C}_{|r_{max}-1|}(B1): \quad \alpha_i = 0$$
$$\mathcal{C}_{r_{max}}(A1) \cap \mathcal{C}_{2r_{max}}(B1): \quad \alpha_i = \pm\arccos\left(\frac{1-3r_{max}^2}{2r_{max}}\right)$$
$$\mathcal{C}_{r_{max}}(A1) \cap \mathcal{C}_{|r_{max}-1|}(B2): \quad \alpha_i = \alpha_2 \pm \arccos\left(\frac{r_2^2+2r_{max}-1}{2r_{max}r_2}\right)$$
$$\mathcal{C}_{r_{max}}(A1) \cap \mathcal{C}_{r_{max}+1}(B2): \quad \alpha_i = \alpha_2 \pm \arccos\left(\frac{r_2^2-2r_{max}-1}{2r_{max}r_2}\right)$$

CORRECTIONS OF MOTIF COUNTS WHEN THE SOMATA HAVE FINITE SIZE

According to model definition, a dendrite center falls approximately on the soma (an assumption suitable for basal dendrites) which occupies a finite space. Consequently, two dendrite centers should not have identical coordinates. The method for computation of the expected motif counts imposes no restrictions on dendrite positions. The finite dimension of the somata is ignored, i.e. it is considered to be negligible compared to the neurite field size or to the connectivity are size, so the probability of the two coinciding dendrite centers becomes zero. However, if the dimension of the somata is not neglected, a correction needs to be applied in order to avoid the cases of overlapping dendrite centers. Here we show the procedure to compute the correction coefficients for the 2- and 3-node motifs. Expressions for motif counts are evaluated in the small neighborhood (a circle of radius δr) of particular points. If the neighborhood is small enough, $\delta r << r_{max}$, we can assume that n_i has approximately constant value in the entire neighborhood.

The correction for the 2-node motifs: For $r_{max} > 1$ dendrite center B1 falls inside the connectivity area of its axon, therefore when integrating over the entire connectivity area of A1 we also include the case $B2 = B1$. To avoid this situations, we exclude a small circle of radius $\delta r << 1$ from the integration domain when we evaluate the expressions for the expected number of 2-node motifs. This is done by subtracting correction coefficients from the numbers obtained using the expressions in the paper. The correction coefficients are the appropriate motif counts evaluated for the circle of radius δr. For the

bidirectional motif M1-2, the correction coefficient is given as:

$$\text{corr-M1-2} = \frac{\Delta_{ad}^2}{2l^2\pi} \int_{-\pi}^{\pi} \int_0^{\delta r} \kappa_1(0,1)\ r\ dr\ d\alpha = \frac{\Delta_{ad}^2}{2l^2\pi} \cdot 2\pi^2\ \delta r^2$$

Given that $\kappa_1(0,1) = 2\pi$ the correction coefficient for motif $M1-2$ is always zero as $2\pi - \kappa_1(0,1) = 0$.

The correction for the situation $\mathbf{B2 = B3 \neq B1}$*:* If $r_{max} \geq 1$ the connection $N_2 \leftrightarrow N_3$ has to exist. Among the considered connectivity patterns this is true only for M13. Other patterns do not have to be considered since they are not considered when computing motif counts. For $r_{max} < 1$,no connection between N_2 and N_3 is possible. This holds for patterns that correspond to motifs M1, M2, M3, M8. The expression for the correction coefficient is:

$$\text{corr-Mi} = \frac{\Delta_{ad}^4 \cdot \delta r^2}{4l^4\pi} \int_{\alpha_2=-\pi}^{\pi} \int_{r_2=0}^{r_{max}} n_i(\alpha_2, r_2, \alpha_2, r_2)\ r_2^2\ dr_2\ d\alpha_2$$

In this case $\|B2B3\| = 0$ and from the definition of κ and λ it follows that:

$$\begin{aligned}
\kappa_1(\alpha_3, r_3) &= \kappa_1(\alpha_2, r_2) \\
\kappa(\alpha_2, r_2, \alpha_3, r_3) &= \begin{cases} 0, & r_{max} < 1 \\ 2\pi, & r_{max} \geq 1 \end{cases} \\
\lambda(\alpha_2, r_2, \alpha_3, r_3) &= \begin{cases} 0, & r_{max} < 1 \\ \kappa_1(\alpha_2, r_2), & r_{max} \geq 1, \quad \|B1B2\| > r_{max} - 1 \\ 2\pi, & r_{max} \geq 1, \quad \|B1B2\| \leq r_{max} - 1 \end{cases}
\end{aligned}$$

The function $n_i(\alpha_2, r_2, \alpha_2, r_2)$ has nonzero values in the following cases:

$$\begin{aligned}
n_1(\alpha_2, r_2, \alpha_2, r_2) &= (2\pi - \kappa_1(\alpha_2, r_2))^2, \quad r_{max} < 1 \\
n_3(\alpha_2, r_2, \alpha_2, r_2) &= \kappa_1(\alpha_2, r_2) \cdot (2\pi - \kappa_1(\alpha_2, r_2)), \quad r_{max} < 1 \\
n_8(\alpha_2, r_2, \alpha_2, r_2) &= \kappa_1(\alpha_2, r_2)^2, \quad r_{max} < 1 \\
n_{13}(\alpha_2, r_2, \alpha_2, r_2) &= \kappa_1(\alpha_2, r_2)^2, \quad r_{max} \geq 1
\end{aligned}$$

The correction for the situation $\mathbf{B2 = B1 \neq B3}$*:* Given that $B2 \in \mathcal{B}_{r_{max}}(A1)$, this is possible only for $r_{max} \geq 1$. In this case the connection $N_1 \leftrightarrow N_2$ has to exist, the possible motifs are M3, M6, M13. Functions κ and λ become:

$$\kappa_1(\alpha_2, r_2) = 2\pi, \quad \kappa(\alpha_2, r_2, \alpha_3, r_3) = \lambda(\alpha_2, r_2, \alpha_3, r_3) = \lambda(\alpha_3, r_3, \alpha_2, r_2) = \kappa_1(\alpha_3, r_3)$$

The correction coefficients for this case are given by the following expressions:

$$\begin{aligned}
\text{corr-Mi} &= \frac{\Delta_{ad}^4 \cdot \delta r^2}{4l^4\pi} \int_{\alpha_2=-\pi}^{\pi} \int_{r_2=0}^{r_{max}} n_i(0, 1, \alpha_3, r_3)\ r_3\ dr_3\ d\alpha_3 \\
n_3(0, 1, \alpha_3, r_3) &= (2\pi - \kappa_1(\alpha_3, r_3))^2 \\
n_6(0, 1, \alpha_3, r_3) &= \kappa_1(\alpha_3, r_3) \cdot (2\pi - \kappa_1(\alpha_3, r_3)) \\
n_{12}(0, 1, \alpha_3, r_3) &= \kappa_1(\alpha_3, r_3) \cdot (2\pi - \kappa_1(\alpha_3, r_3)) \\
n_{13}(0, 1, \alpha_3, r_3) &= \kappa_1(\alpha_3, r_3)^2
\end{aligned}$$

The correction for the situation $\mathbf{B2} \neq \mathbf{B3} = \mathbf{B1}$*:* This is also possible only for $r_{max} \geq 1$, in which case the connection $N_1 \leftrightarrow N_3$ has to exist. The functions κ and λ are:

$$\kappa_1(\alpha_3, r_3) = 2\pi, \;\; \kappa(\alpha_2, r_2, \alpha_3, r_3) = \lambda(\alpha_2, r_2, \alpha_3, r_3) = \lambda(\alpha_3, r_3, \alpha_2, r_2) = \kappa_1(\alpha_2, r_2)$$

The pattern that should be corrected in this case corresponds to M13.

$$\begin{aligned} \text{corr-M13} &= \frac{\Delta_{ad}^4 \cdot \delta r^2}{4 l^4 \pi} \int_{\alpha_2=-\pi}^{\pi} \int_{r_2=0}^{r_{max}} n_i(\alpha_2, r_2, 0, 1)\; r_2 \; dr_2 \; d\alpha_2 \\ n_{13}(\alpha_2, r_2, 0, 1) &= \kappa_1(\alpha_2, r_2)^2 \end{aligned}$$

The correction for the situation $\mathbf{B2} = \mathbf{B3} = \mathbf{B1}$*:* This can happen for $r_{max} \geq 1$, and the possible motif is M13. In this case it holds that: $\kappa_1(\alpha_2, r_2) = \kappa_1(\alpha_3, r_3) = \kappa(\alpha_2, r_2, \alpha_3, r_3) = \lambda(\alpha_2, r_2, \alpha_3, r_3) = \lambda(\alpha_3, r_3, \alpha_2, r_2) = 2\pi$. The correction coefficient is:

$$\text{corr-M13} = \frac{\Delta_{ad}^4 \cdot \delta r^4}{4 l^4} 4\pi^2$$

Summary of the corrections for the 3-node motifs: Finally, the counts for the particular motifs are:

$$\text{corr-M1} = h(1 - r_{max}) \cdot \frac{\Delta_{ad}^4 \cdot \delta r^2}{4 l^4 \pi} \int_{\alpha=-\pi}^{\pi} \int_{r=0}^{r_{max}} (2\pi - \kappa_1(\alpha, r))^2 \; r^2 \; dr \; d\alpha$$

$$\begin{aligned} \text{corr-M3} = \frac{\Delta_{ad}^4 \cdot \delta r^2}{4 l^4 \pi} \int_{\alpha=-\pi}^{\pi} \int_{r=0}^{r_{max}} \big(& \kappa_1(\alpha, r) \cdot (2\pi - \kappa_1(\alpha, r)) r^2 \cdot h(1 - r_{max}) + \\ & + (2\pi - \kappa_1(\alpha, r))^2 \; r \cdot h(r_{max} - 1)\big) \; dr \; d\alpha \end{aligned}$$

$$\text{corr-M6} = h(r_{max} - 1) \cdot \frac{\Delta_{ad}^4 \cdot \delta r^2}{4 l^4 \pi} \int_{\alpha=-\pi}^{\pi} \int_{r=0}^{r_{max}} \kappa_1(\alpha, r) \cdot (2\pi - \kappa_1(\alpha, r)) \; r \; dr \; d\alpha$$

$$\text{corr-M8} = h(1 - r_{max}) \cdot \frac{\Delta_{ad}^4 \cdot \delta r^2}{4 l^4 \pi} \int_{\alpha=-\pi}^{\pi} \int_{r=0}^{r_{max}} \kappa_1(\alpha, r)^2 \; r^2 \; dr \; d\alpha$$

$$\text{corr-M12} = h(r_{max} - 1) \cdot \frac{\Delta_{ad}^4 \cdot \delta r^2}{4 l^4 \pi} \int_{\alpha=-\pi}^{\pi} \int_{r=0}^{r_{max}} \kappa_1(\alpha, r) \cdot (2\pi - \kappa_1(\alpha, r)) \; r \; dr \; d\alpha$$

$$\text{corr-M13} = h(r_{max} - 1) \cdot \frac{\Delta_{ad}^4 \cdot \delta r^2}{4 l^4 \pi} \int_{\alpha=-\pi}^{\pi} \int_{r=0}^{r_{max}} \left(\kappa_1(\alpha, r)^2 \; r^2 + 2\kappa_1(\alpha, r)^2 \; r\right) \; dr \; d\alpha - \frac{\Delta_{ad}^4 \; \delta r^4 \; \pi^2}{l^4}$$

In the last equation (for M13) the coefficient for $B2 = B3 = B1$ is subtracted from the rest of the expression because this situation is already taken into account twice, once for $B1 = B2 \neq B3$, and once for $B2 \neq B3 = B1$. The small coefficient δr is a new model parameter that should be proportional to the soma radius. In the results shown in this paper it is fixed to $\delta r = 0.01 r_{max}$.
