# Supplementary material for: The effects of neuron morphology on graph theoretic measures of network connectivity: the analysis of a two-level statistical model
Source: Front Neuroanat. 2015 Jun 10;9:76. doi: 10.3389/fnana.2015.00076 (PMC4461825; doi:10.3389/fnana.2015.00076)
Supplement: Supplementary file 3 [file Presentation3.PDF]

Frontiers in Neuroanatomy

Research Article
30 April 2015

Supplementary material 3: Comparison between theoretical results and numerical simulations

Jugoslava Aćimović [1,*], Tuomo Mäki-Marttunen [2,1] and Marja-Leena Linne [1]

[1] *Computational Neuroscience Group, Department of Signal Processing, Tampere University of Technology, Tampere, Finland,*
[2] *Psychosis Research Centre, Institute of Clinical Medicine, University of Oslo, Oslo, Norway.*

Correspondence*:
Jugoslava Aćimović
Computational Neuroscience Group, Department of Signal Processing, Tampere University of Technology, P.O.Box 553, 33101 Tampere, Finland,
jugoslava.acimovic@tut.fi

In Results section, we compared our theoretical model to the standard types of networks, uniform random and locally coupled networks. We compute the t-coefficients for motif counts, as well as the small-world coefficient. The model size becomes an issue in these comparisons: The theoretical model, solved analytically, is practically infinite while the simulated models must be of finite size. In most of the computations, we do not have to make any assumption about the population size, we can consider that every neuron has an infinite number of other neurons available. This issue particularly affects the path length computation, a global measure of network structure. Motif counts and clustering coefficient (as defined in this study) are local measures and, as long as we simulate sufficiently big networks and avoid boundary conditions (by projecting the model on a torus), they are not significantly affected by the model size.

We illustrate steps of the iterative algorithm for the path-length computation presented in Methods section. For each step, we compare the obtained results with the results from an equivalent simulated finite size model.

EXPECTED HARMONIC PATH LENGTH COMPUTATION

The path length analysis requires different approach compared to all other computations used in this study. It is a global measure and depends on the network size, so we have to artificially limit our infinite-size model by placing it on a planar area of size $L \times L$ projected on a torus (as shown in Figure 1 in Methods section). The methodology used to analyze motifs and clustering coefficient cannot be applied to path length, as the complexity of the computations increases exponentially with the path length size. Instead, we used an iterative approach summarized by the following equations:

$$PL = \sum_k \frac{1}{k} \cdot P\left(PL = k\right) \tag{1}$$

$$P\left(PL=k\right) \quad = \quad \frac{2\pi\Delta_{ad}^{2}}{l^{2}\mathcal{N}}\int_{\Omega_{1}(r)}\left(P\left(PL\leq k\mid r_{2}\right)-P\left(PL\leq k-1\mid r_{2}\right)\right)\,r_{2}\,dr_{2} \tag{2}$$

$$P\left(PL=k,\;r_{2}\right) \quad = \quad \left(P\left(PL\leq k\mid r_{2}\right)-P\left(PL\leq k-1\mid r_{2}\right)\right)\cdot P(r_{2}) \tag{3}$$

$$P\left(PL\leq k\mid r_{2}\right) \quad = \quad 1-\left(1-\nu(k-1\mid r_{2})\right)^{\mathcal{N}-2} \tag{4}$$

$$\nu(k-1\mid r_{2}) \quad = \quad \frac{\Delta_{ad}^{2}}{2\pi l^{2}\mathcal{N}}\int\int_{\Omega_{2}(\alpha,r)}P\left(PL\leq k-1\mid r\right)\cdot\kappa\left(\alpha,r,0,r_{2}\right)\cdot r\,dr\,d\alpha \tag{5}$$

Equation 1 shows how to compute the harmonic path length from the known probabilities of path lengths, $P(PL=k)$. The harmonic path length is a harmonic mean over path lengths obtained for every pair of nodes in the network. As we consider a homogeneous population with random axon orientations, it is sufficient to compute only the path lengths from one node to all others. This node is called N_1 and the polar coordinate system is fixed to it, with the coordinate center in the axon center A1. All radial coordinates $(r,\ r_2)$ are computed with respect to A1.

Equation 2 gives a relation between the shortest path length probability $P(PL=k)$ and the conditional probability $P\left(PL\leq k\mid r_2\right)$. The conditional probability expresses the chance of having the shortest path length not bigger than k for a neuron with the radial distance of the dendrite center fixed to r_2. Next, Equation 3 shows how to compute $P(k,\ r_2)$, the joint probability of having the shortest path length k and the radial coordinate of the dendrite center r_2. This conditional probability is a function of $\nu(k-1|r_2)$, as described by Equation 4. This last function, $\nu(k-1|r_2)$, expresses the probability that one fixed neuron N_i has the shortest path length $k-1$ and the axon center inside the connectivity area of dendrite $B2(0,r_2)$. This is an assisting function needed to compute the conditional probability. Equations 4 and 5 represent a pair of iterative equations, and the first step in computation of the harmonic path length.

The domains of integration $\Omega_1(r)$ and $\Omega(\alpha,r)$ define the range of values for the polar coordinates in computation of the probabilities $P(k)$ and $P(PL\leq k\mid r_2)$, respectively. The first domain defines only the radial coordinate, while the second depends on both, radial and angular coordinates.

CONDITIONAL PROBABILITY: $P\left(PL\leq K\mid R_2\right)$

The first step in path length computation is to solve the pair of iterative equations and compute the conditional probability $P\left(PL\leq k\mid r_2\right)$. Figure 1 compares the result obtained from Equations 4 and 5 with those computed by simulating an equivalent model. In both cases, the considered model parameters are $\Delta_{ad}=1,\ l=0.3,\ N=3600$. We considered the same values of the normalized effective radius as before, $r_{max}\in\{0.1,0.3,0.5,0.7,1,1.7,2,5,10\}$, but on the figure we show only second to fifth of them. Each row on the figure corresponds to one value of the normalized effective radius, indicated at the end of each row. The smallest value of r_{max} is not considered because it gives a disconnected network where most of the neuron pairs have no traversable path to each other. For the larger r_{max}, the obtained conditional probabilities behave similarly as those in Figure 1, with more visible effects of the finite simulation size.

In most of the cases the analyzed conditional probability resembles a step function with faster or slower decay. The probabilities obtained for $r_{max}=0.3$ somewhat deviate from this. The networks considered in this case are very sparse with small node degree, and the integral in Equation 5 averages out a small number of neighbors. For some of those neighbors the function κ might have discontinuities, which is possible according to its definition. When the number of neighbors is small, such discontinuities affect the integral in Equation 5 more, and the evaluated probabilities deviate from the step-like functions. In the simulated model, this sparseness manifests differently. Small connectivity results in certain number of disconnected

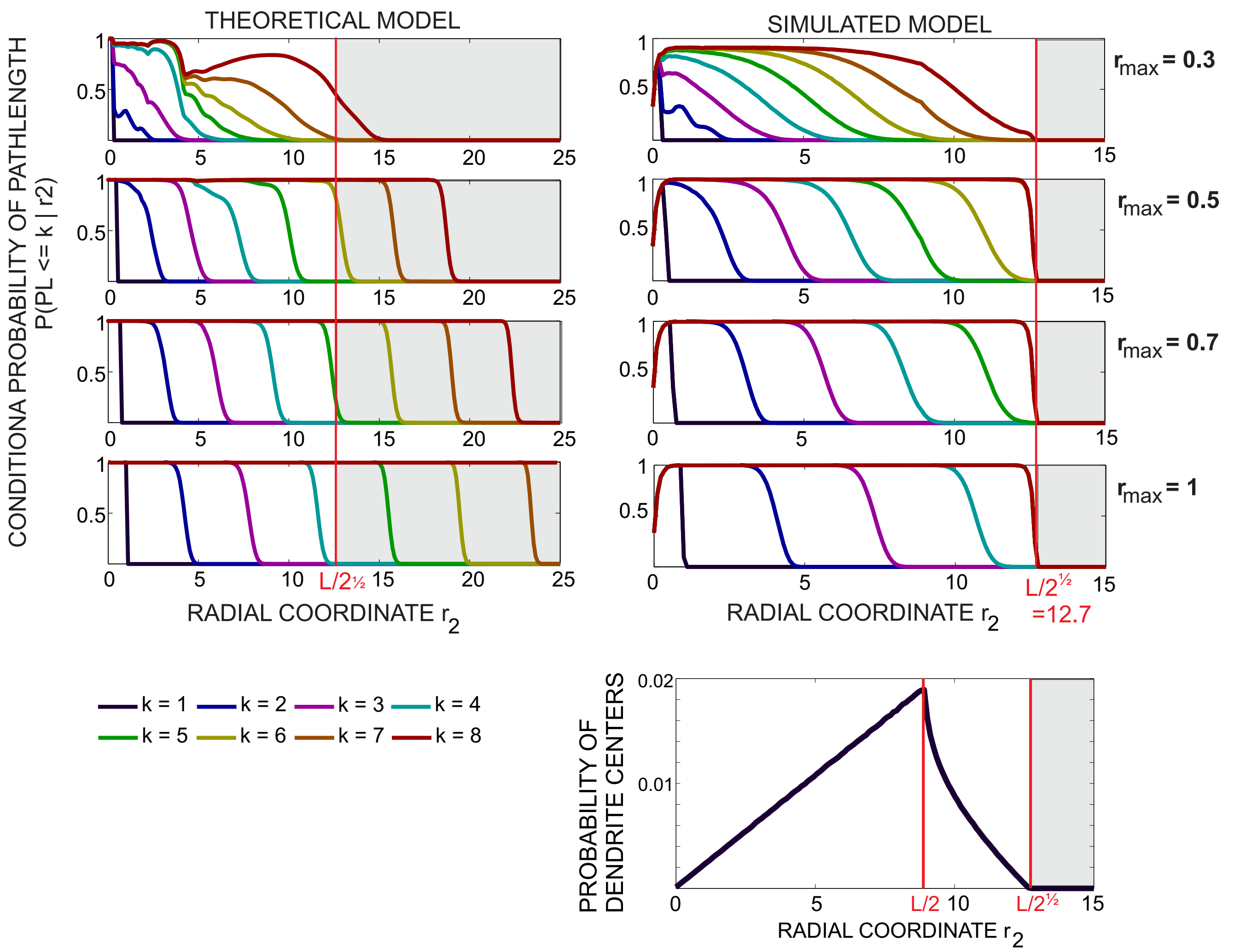

Figure 1. Conditional probability $P\,(PL \leq k \mid r_2)$ computed from the theoretical (left column) and the simulated model (right column). For a given radial coordinate of a dendrite center r_2 this probability shows how likely is that such neuron has path length not bigger than k from the neuron N_1 at the origin. Each row corresponds to one value of the normalized effective radius r_{max}, indicated at the end of the row. The x axis shows the radial coordinate r_2 and the y axis is the conditional probability. Each color corresponds to one value of the path length k, the color code is given at the bottom of the figure. The vertical red line, indicated on all panels, marks the maximal radial distance for the given model parameters equal to $\frac{L}{\sqrt{2}}$ (half the diagonal of the model area). In order to emphasize the difference between the theoretical and the simulated model, we also computed some conditional probabilities for 'too big' radial coordinates. These cases are inside the gray areas on the panels. The conditional probability of simulated model drops for small and large values of the radial coordinate. This is due to the small number of neurons available for those values of r_2. This is additionally illustrated on the bottom figure that shows the probability of finding a dendrite center with the given radial coordinate. This probability is maximal for $\frac{L}{2}$ and very small for $r_2 = 0$ and $r_2 = \frac{L}{\sqrt{2}}$.

neuron pairs, assigned the Inf path length. Such pairs are removed from further computations, and the obtained probabilities are still monotonous.

In the simulated model, the conditional probability drops for very small and very large values of the radial coordinate. This is due to the small number of dendrite centers available for those radial coordinates, an effect additionally illustrated on the bottom panel on Figure 1. This panel shows the probability of a dendrite center for each possible radial coordinate. The probability increases linearly until $r_2 = \frac{L}{2}$. For the

bigger values of the radial coordinate the cut-off effect appears in the finite size model, and the probability drops to zero for $r_2 = \frac{L}{\sqrt{2}}$, the maximal possible radial coordinate.

For the same reason, the conditional probability in the simulated model quickly drops to zero when the maximal radial coordinate is reached (red line on the panels). This does not happen for the theoretical model, which behaves as an infinite size model. For larger radial coordinates the equations give conditional probabilities for bigger values of k, eventually until both k and r_2 become infinite. But, the Equation 3 requires computation of $P(r_2)$, the probability to find a dendrite center with the radial coordinate r_2, we have to limit the model size. This is done by 'cutting out' an area of the size $L \times L$ and projecting it to the torus, which results in cutting the conditional probabilities with the red line. In the next step, we do not consider the functions that fall inside the gray areas.

JOINT PROBABILITY: $P(K,\ R_2)$

In the next step, we compute the joint probability $P(k,\ r_2)$ using the Equation 3. The obtained result is compared to the simulated model on Figure 2. The figure is in the same format and with the same color code as Figure 1. Here, also, the effect of the finite size model is visible. In the simulated model, the joint probability decreases towards zero for $r_2 = \frac{L}{\sqrt{2}}$. In the theoretical model, the probabilities increase infinitely, and they are cut at $r_2 = \frac{L}{\sqrt{2}}$ to make the model of finite size.

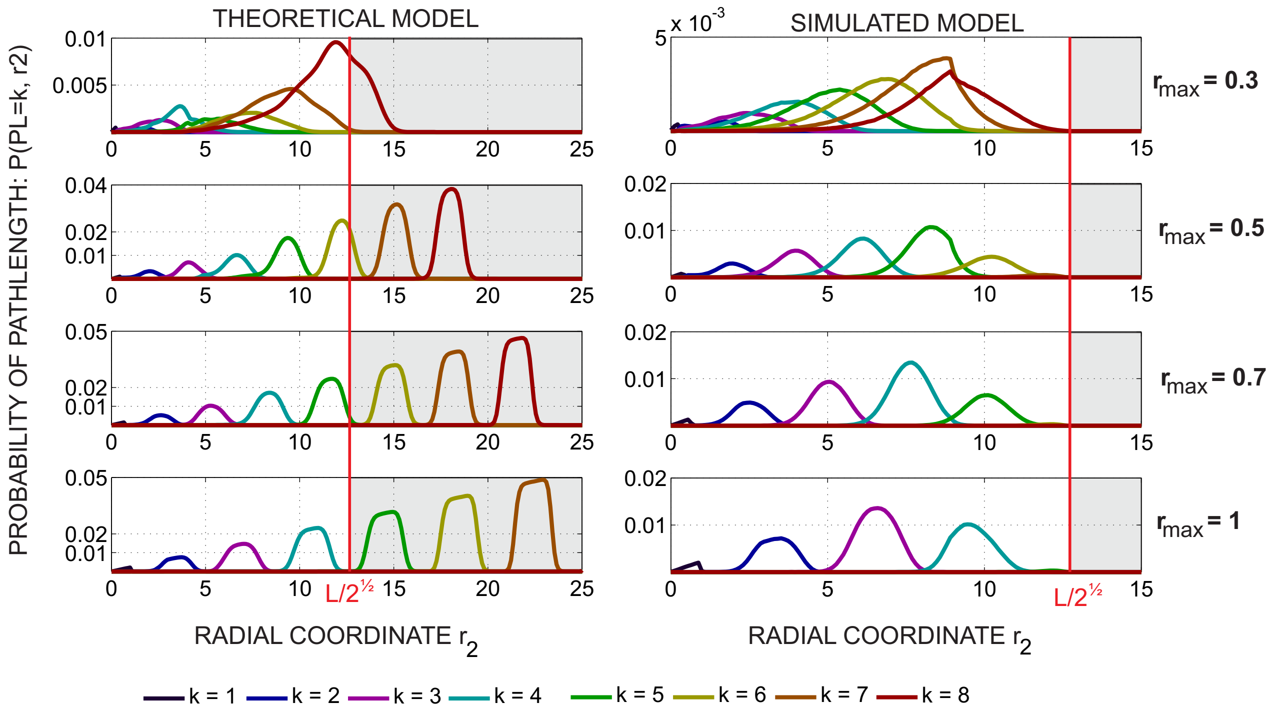

Figure 2. Figure illustrates the second step in the path length computation, the joint probabilities $P(PL = k,\ r_2)$ computed for the theoretical (left column) and simulated model (right column). The radial coordinate is show on the x axis, and the joint probability on the y axis. Different rows correspond to different values of the normalized effective radius (indicated at the end of each row). Different colors correspond to different values of path length k, the color code is shown on the bottom of the figure. Gray areas correspond to probabilities obtained for r_2 bigger than the maximal allowed for the given model size, still they are shown to illustrate the differences between the theoretical and the simulated model.

PROBABILITY OF PATH LENGTH, HARMONIC PATH LENGTH

From the previous results and the Equation 2 we compute the probability of having path length k, which is illustrated on Figure 3. The two panels in the upper row show the probability of path length $(P(PL = k))$ obtained for the theoretical model (panel A) and the simulated model (panel B). The path lengths are indicated on x axis, the probabilities on y axis, and different colors correspond to different normalized effective radius r_{max}. The difference between the two results is not so evident as in the previous steps. The simulated probabilities have slightly higher values for bigger path lengths. The theoretical probabilities drop to zero relatively fast, which is the consequence of model size limitation and the cut-of of joint probabilities (red line in Figure 2).

The bottom panel C compares the two obtained path lengths, the dashed line corresponds to simulated model and the full line to theoretical model. Individual data points are colored using the same code as in panels A and B. For every model parameter, the path lengths of the theoretical model are somewhat smaller than those obtained from the simulated model, as a result of the described difference in path length probabilities. The difference between path lengths decreases as the normalized effective radius increases.

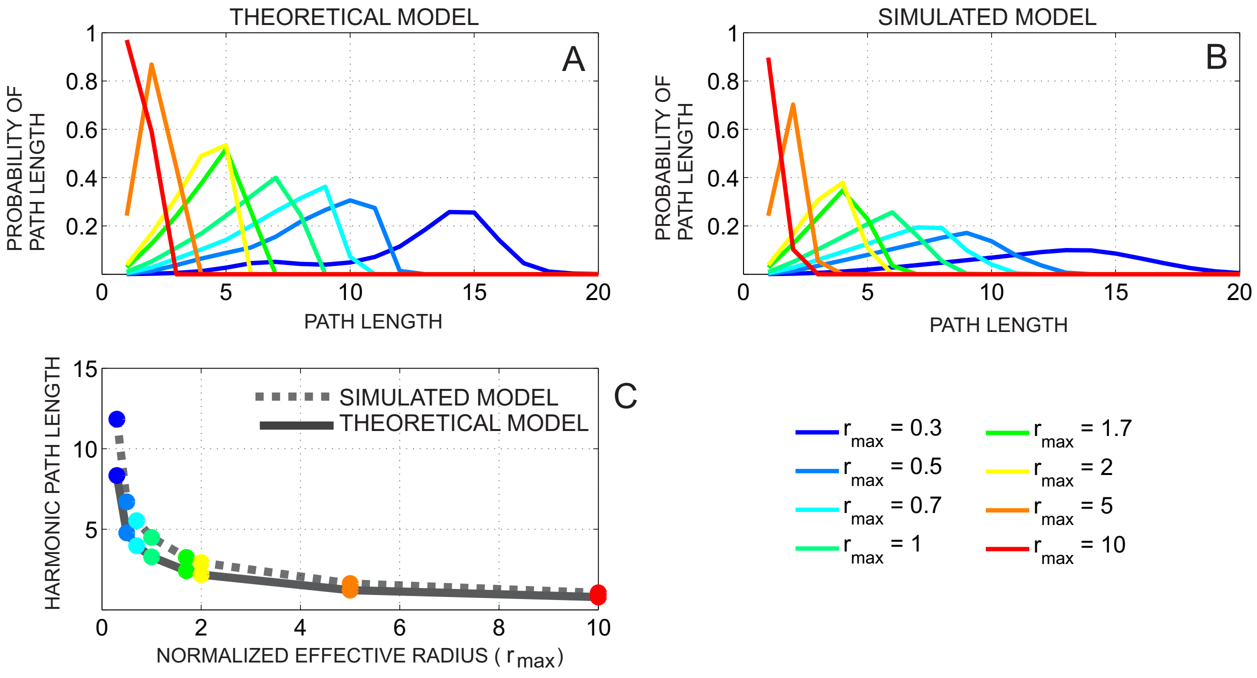

Figure 3. Panels A and B show the probability of certain path length, panel A is obtained from Equation 2 and panel B from simulation of the equivalent model. The x axis shows the path lengths, and y axis the corresponding probabilities. Different colors indicate different values of the normalized effective radius (color code is indicated under the panel B). Panel C, finally, shows the harmonic path lengths obtained for different r_{max} and for both considered models. The dashed line indicates simulated results, while the other one corresponds to the theoretical model. Colored dots indicate the values of r_{max}, consistent with the panels A and B.

THE EFFECT OF FINITE MODEL SIZE ON THE ANALYZED CONNECTIVITY PARAMETERS

Figures 4 and 5 compare the connectivity measures calculated using our analytical solutions with the values obtained from the corresponding numerical simulations. Analytically computed connectivity measures represent the grand average over all the nodes in an infinite-size network. Numerical simulations can be done only for the finite size models, and only a finite number of iterations can be done for each simulation. The results presented in this section illustrate the discrepancies between the analytical and the simulation-based results arising from these differences. We also demonstrate that all the conclusions presented in the paper for the analytical results also hold for the results obtained from simulations, even with some quantitative differences.

In addition, we examine how both the analytical and the simulation-based connectivity measures depend on the size of the considered model. The model size determines the maximal path length in a network and also the cut-off, described in the previous sections, in our iterative method for the harmonic path length computation. Consequently, the small-world coefficient also depends on the model size. In addition, the reference uniform random and locally coupled networks are simulated for the finite size networks.

Figure 4 presents the results obtained for the mid-range of values for the normalized effective radius, i.e. for $r_{max} \in \{0.5, 0.7, 1, 1.7, 2, 5\}$. This also includes the interval $r_{max} \in [1, 2]$ where the variability of connectivity repertoires is maximized and the obtained models approach to some extent the small-world networks. Panels on the left plot the considered connectivity measures: clustering coefficient, harmonic path length, and two versions of the small-world coefficient computed according to (Watts and Strogatz (1998)) and (Telesford et al. (2011)). Panel C shows the logarithm of the harmonic path length, in order to be consistent with Figure 9 from Results and Figure 5 from this section (these two figures show a wide range of values for the path length that requires a logarithmic scale). Panels on the right show absolute value of the differences between the analytical results and the corresponding simulated results (unlike Panel B, Panel C uses the standard linear scale). The x axis shows the considered values of the normalized effective radius on a logarithmic scale. Different colors correspond to different model sizes. We considered the networks with $\mathcal{N} = 1600$ nodes (yellow and green), $\mathcal{N} = 2500$ nodes (orange and turquoise), $\mathcal{N} = 3600$ nodes (red and blue), and $\mathcal{N} = 4900$ nodes (dark red and dark blue). The analytical results are marked with yellow-to-red lines, the simulated results and the differences between analytical and simulated results are marked with green-to-blue lines.

Panels A and B illustrate the clustering coefficient. The analytical and simulated results show good agreement with the relative mismatch of about 0.1%. This mismatch does not seem to depend on the model size, as shown in Panel B. The definition of clustering coefficient adopted in this study, as well as the expected motif counts that are used to compute the clustering coefficient, quantify the local connectivity in a network. As long as the considered network is sufficiently larger than the connectivity area of a neuron the finite-size effects do not influence the clustering coefficient computation.

Panel C shows the harmonic path length obtained using the iterative method (discussed in the previous section) and the numerical simulations of the corresponding model. As already discussed in the previous section, the simulations always give bigger values of the harmonic path length than the iterative method. The absolute difference between the obtained results increases with the model size, as shown in Panel D. For bigger networks the range of values for the harmonic path length is also bigger, as well as the mismatch between the analytical and simulation-based results. This is particularly visible for the smaller values of the normalized effective radius that give longer path lengths. The cut-off effect, discussed in previous sections, appears for any finite size network, so the mismatch cannot be removed by changing the model size. The discrepancy might be decreased by increasing the number of iterations of numerical simulations (we used only five iterations to compute the presented results).

The small-world coefficients shown in Panels D-G combine the clustering coefficient and the path length, and the discrepancy between analytical and simulation-based results accumulates. In addition, the referent uniform random and locally coupled networks are also simulated for the models of finite size

and finite number of iterations. The dependency between the absolute error and the simulated model size is less evident than for the harmonic path length (as shown in Panels F and H). Even with the illustrated discrepancies, the small-world coefficients obtained from simulations behave similarly as the coefficients computed from the analytical expressions. In other words, the small world coefficients reach their optimal values in the same intervals as those reported in Figure 9, i.e. SW_{ws} becomes maximal for $r_{max} \in [0.7, 1]$ while SW_q becomes close to zero for $r_{max} \in [1, 2]$.

The small-world coefficients from (Watts and Strogatz (1998)) depends more on the model size than on the method used to compute it (i.e. analytical or the simulation-based method). From Panels E-F, the discrepancy induced by the selected method is about ten times smaller than the discrepancy caused by the selection of the model size. The small-world coefficient computed from (Telesford et al. (2011)) is more sensitive to the selected methodology as well as the model size, as illustrated in Panels G-H. When increasing the model size, the value of the coefficient decreases and becomes closer to zero for each value of the effective radius. This coefficient should be around zero for the networks with small-world properties. Therefore, in some cases, the effect of the model size might affect whether we recognize a network as small-world or not (see the analytical solutions for $\mathcal{N} = 1600$ (yellow) and $\mathcal{N} = 4900$ (dark red) in Panel G).

Figure 5 repeats the result from Figure 4 but it also includes small and large values of the effective radius. The case for $r_{max} = 0.1$ is not considered as it gives zero clustering coefficient, very large harmonic path length, and possibly disconnected networks. The analysis of the clustering coefficients shows a significant mismatch between the analytical and the simulation-based result for the largest considered value of the normalized effective radius $r_{max} = 10$. This parameter value imposes large number of connections for each node in the network. For the given model parameters, $D_{ad} = 1$, $l = 0.5$, and $\mathcal{N} \in \{1600, 2500, 3600, 4900\}$, each node is connected to 78%, 50%, 35, and 25% of the network, respectively. The smaller among the considered networks have high connectivity, which increases the average clustering coefficient in the network and causes the mismatch visible in Panels A and B.

As the normalized effective radius decreases the mismatch between the analytically computed path length and the path length obtained from simulations increases. For small values of the normalized effective radius the path length is long and the variance in the simulated data is high. The finite number of simulation iterations significantly affects the obtained path length and causes the mismatch visible in Panels C and D in Figure 5.

The cumulative error of clustering coefficient and path length computations is visible in Panels E-H which illustrate the small-world coefficients (see for example SW_q for $r_{max} = 0.3$ and $r_{max} = 10$). The major discrepancies between the simulated and the analytical results appear for very large or very small values of the normalized effective radius, while the model approaches the small-world networks only in between these extreme values.

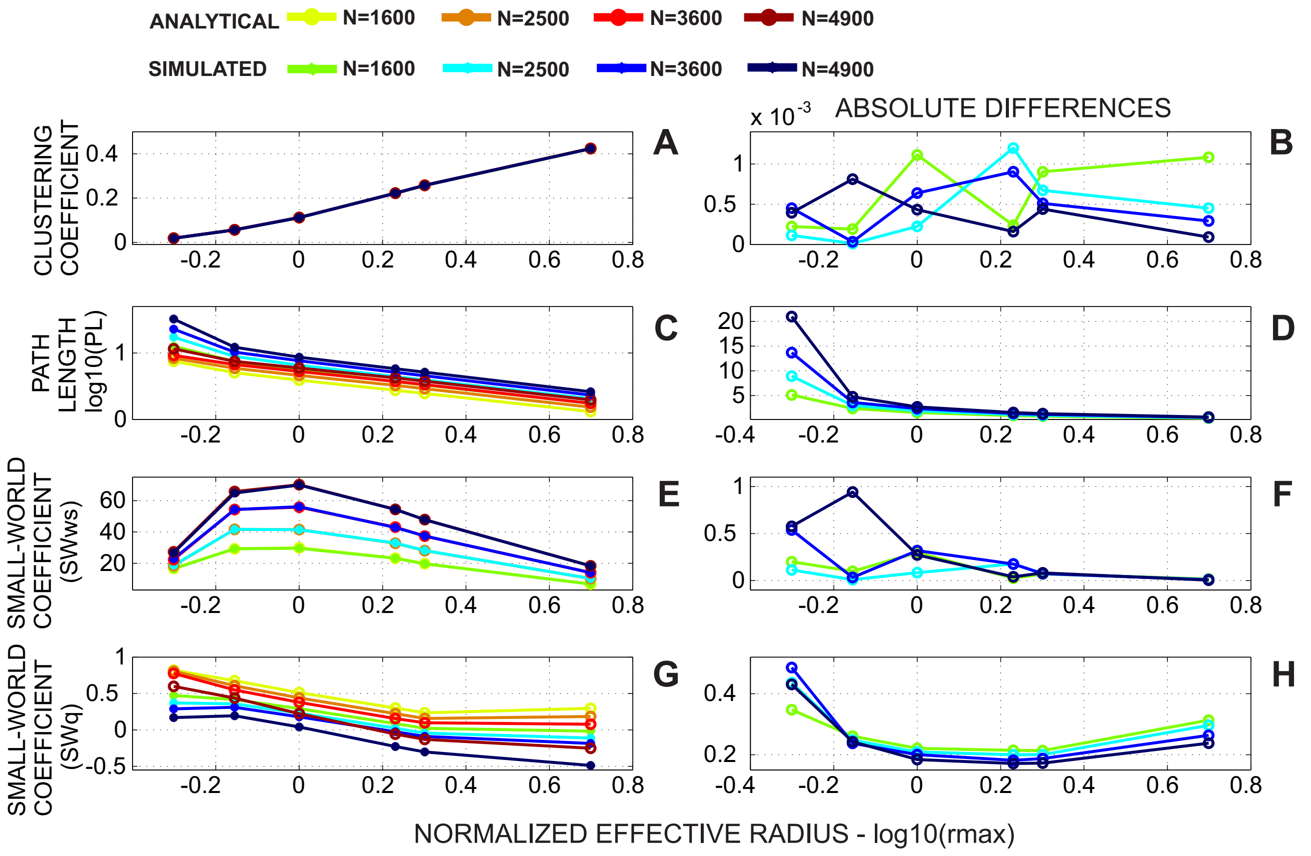

Figure 4. Comparison between the connectivity measures obtained from the analytical solutions and from the numerical simulations of the same model. Figure illustrates the discrepancies caused by the finite size of the simulated model and the finite number of simulation iterations. Panels A-B illustrate the obtained clustering coefficients. Panels C-D compare the harmonic path lengths. Panel C shows the logarithms of the actual path lengths in order to be consistent with Figure 9 in the manuscript and Figure 4 below. These two figures include the path lengths for the small values of r_{max}, i.e. for $r_{max} < 0.5$, which are much longer than the path lengths obtained for $r_{max} \geq 0.5$. Logarithmic scale is necessary to show all these values in the same figure. Panel D uses the standard scale (and not the logarithmic) to compare the path lengths. Panels E-F compare the small-world coefficients computed according to the standard Watts-Strogatz equation. Panels G-H also illustrate the small-world coefficients but computed using the updated definition from (Telesford et al. (2011)). Figure shows the results obtained for the middle range of values for the normalized effective radius, i.e. $r_{max} \in \{0.5, 0.7, 1, 1.7, 2, 5\}$. The logarithm of the normalized effective radius ($\log_{10}(r_{max})$) is shown in the x axis for all the panels in the figure. Different colors correspond to different model sizes, we analyzed networks with $\mathcal{N} \in \{1600, 2500, 3600, 4900\}$ nodes. Other model parameters: $D_{ad} = 1$, $l = 0.5$ (sparse networks). Analytical results are shown by yellow-to-red lines and the simulated results by green-to-blue lines (see the legend above the figure). The right column shows the absolute difference between simulated and analytical results, the color code on these panels is the same as for the simulated results in the left column.

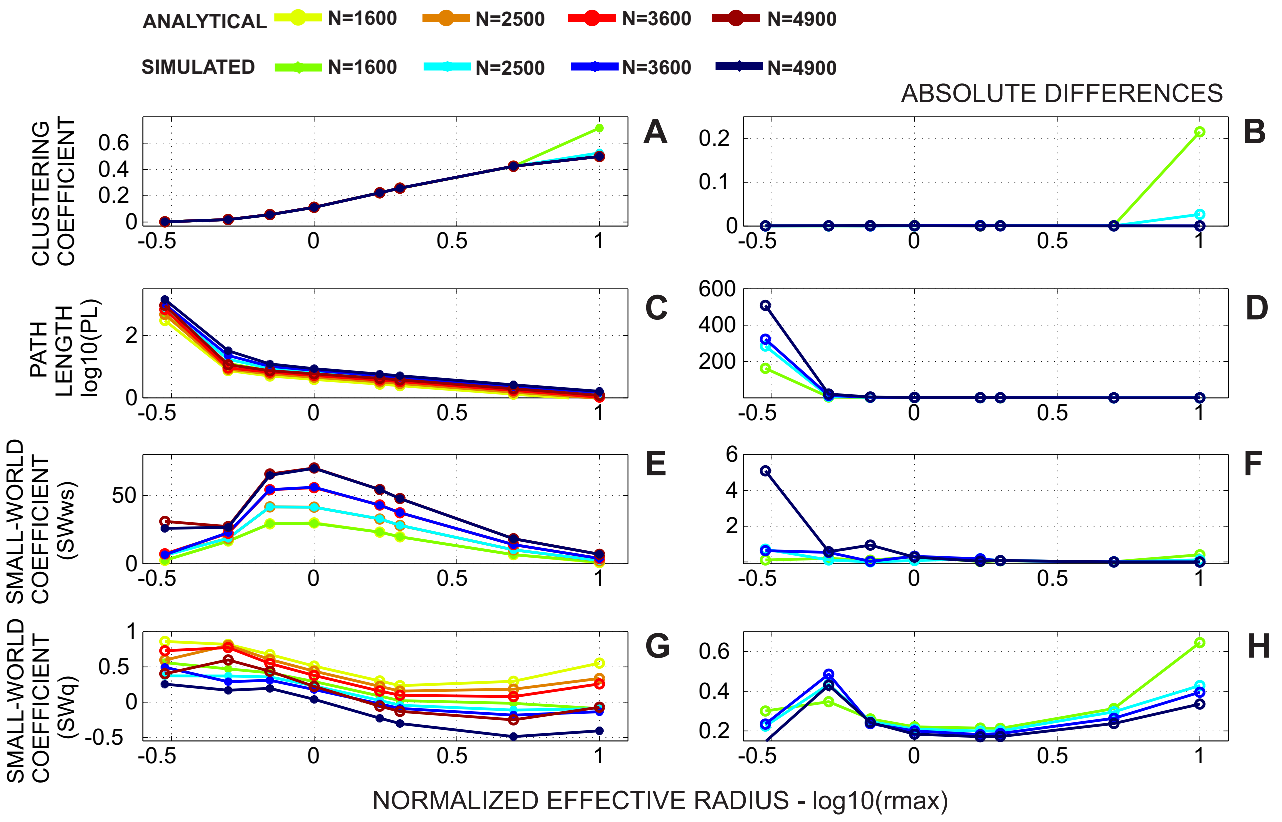

Figure 5. This figure repeats the results shown in Figure 4 but includes small and large values for the normalized effective radius, i.e $r_{max} \in \{0.3, 0.5, 0.7, 1, 1.7, 2, 5, 10\}$ (the network obtained for $r_{max} = 0.1$ is not considered as it results in zero-clustering coefficient and very long path length). Small and large values of the normalized effective radius cause bigger discrepancies between the analytically computed connectivity measures and the corresponding measures obtained from numerical simulations.
